# Supplementary figures and images for: Shared genetic loci between depression and cardiometabolic traits
Source: PLoS Genet. 2022 May 13;18(5):e1010161. doi: 10.1371/journal.pgen.1010161 (PMC9170110; doi:10.1371/journal.pgen.1010161)

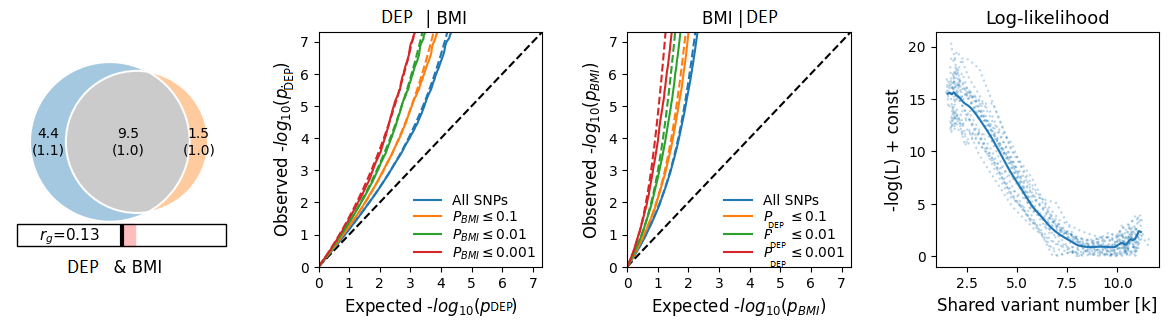

Supplement: S1 Fig — (TIF) [file pgen.1010161.s001.tif]

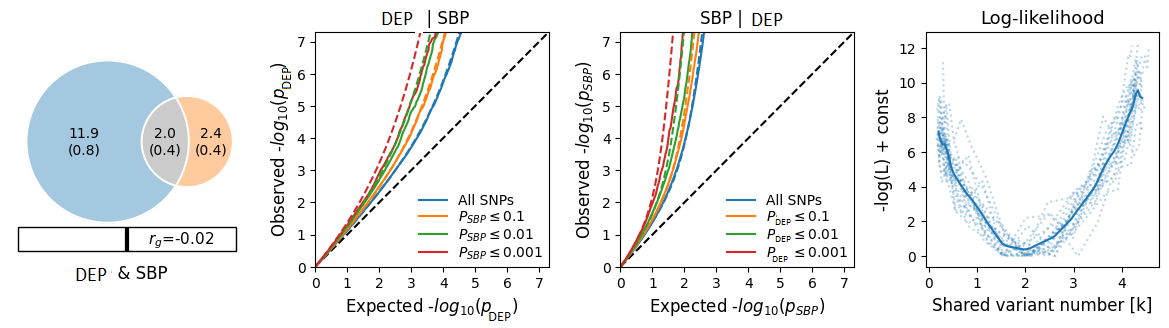

Supplement: S2 Fig — (TIF) [file pgen.1010161.s002.tif]

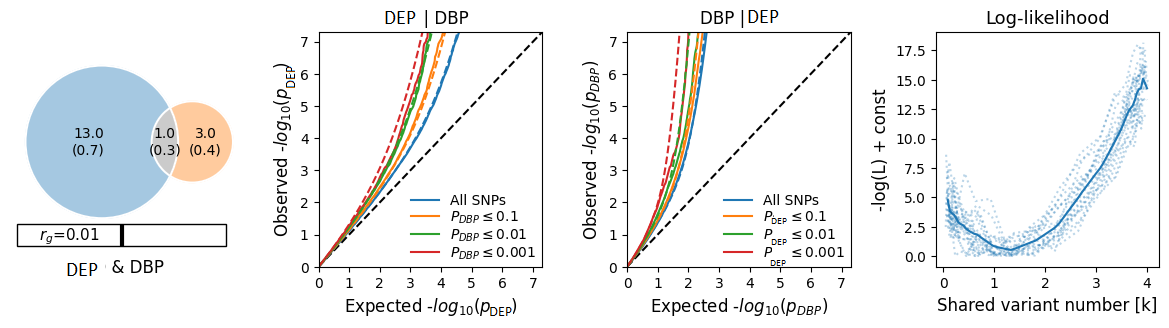

Supplement: S3 Fig — (TIF) [file pgen.1010161.s003.tif]

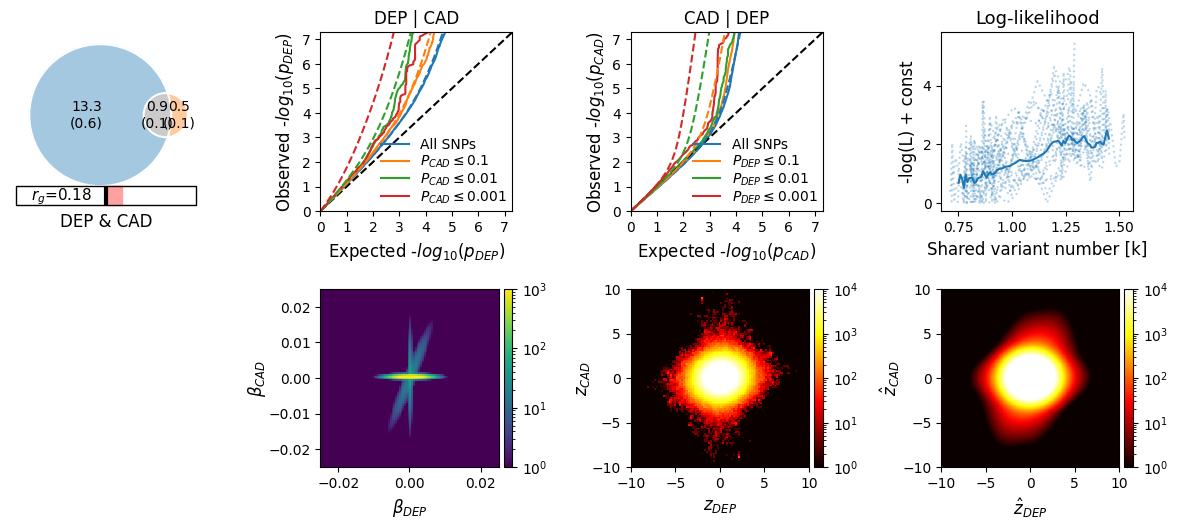

Supplement: S4 Fig — (TIF) [file pgen.1010161.s004.tif]

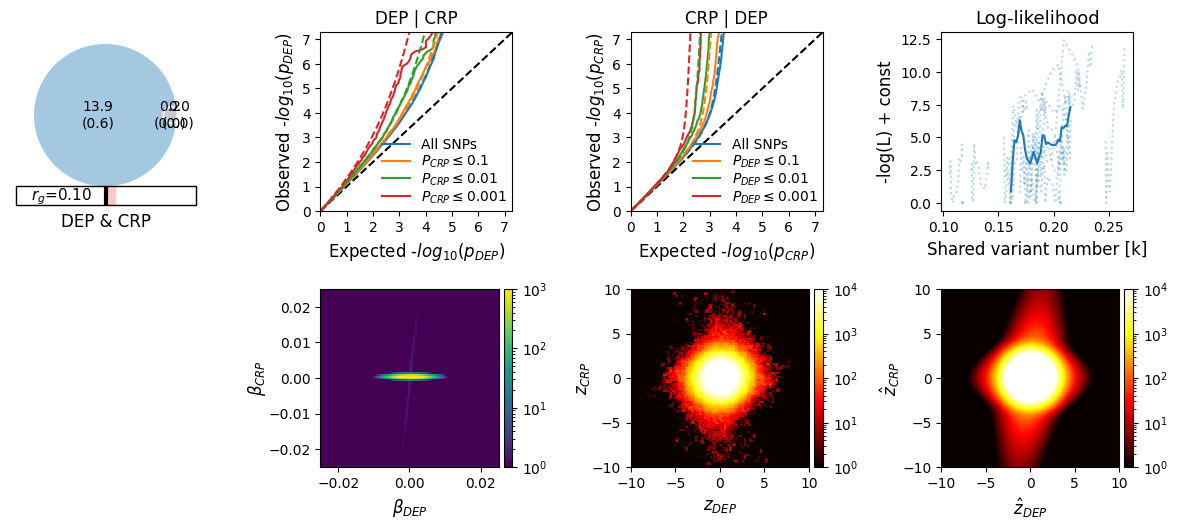

Supplement: S5 Fig — (TIF) [file pgen.1010161.s005.tif]

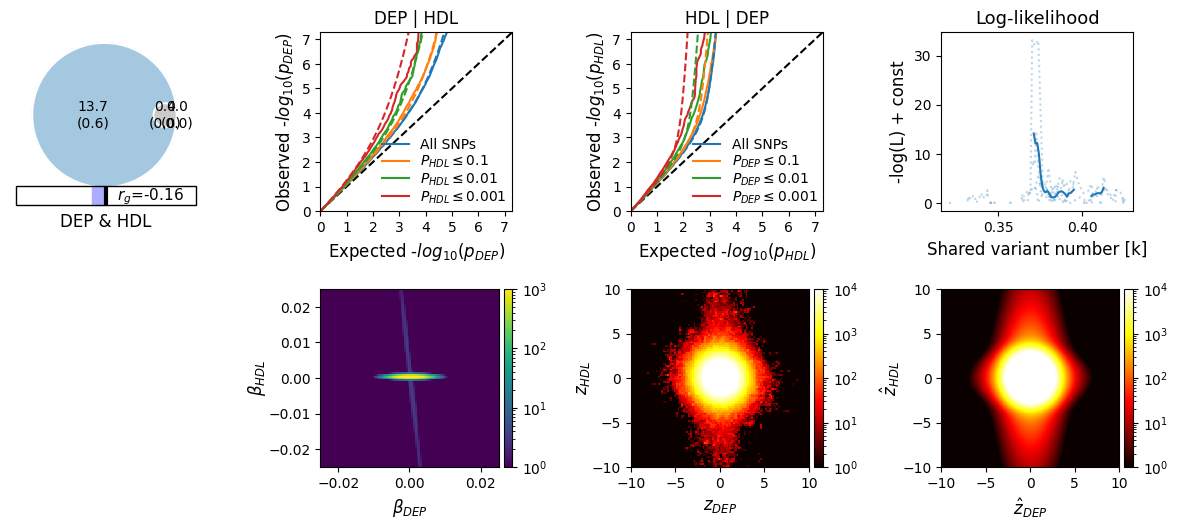

Supplement: S6 Fig — (TIF) [file pgen.1010161.s006.tif]

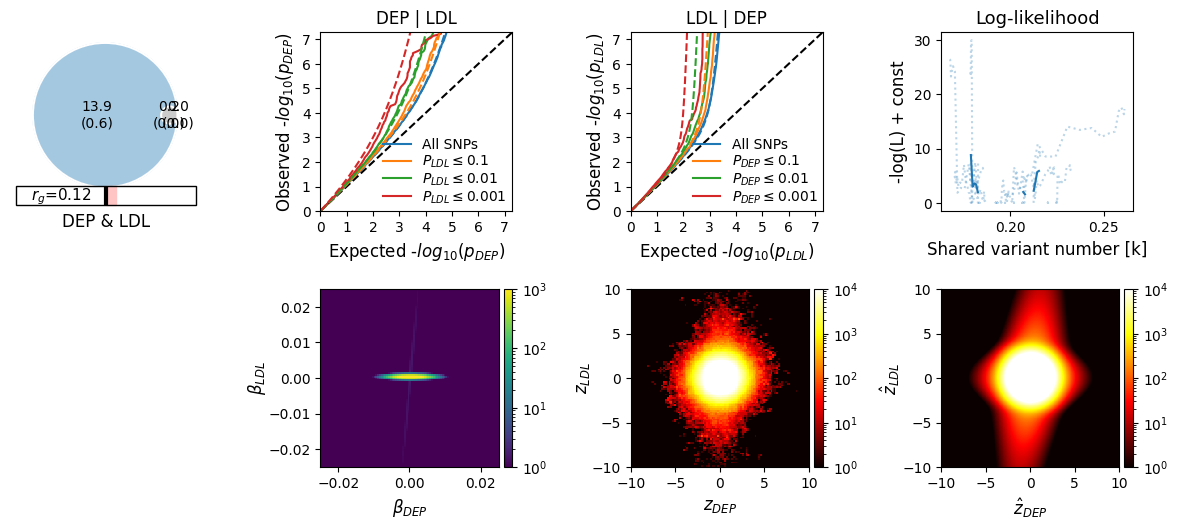

Supplement: S7 Fig — (TIF) [file pgen.1010161.s007.tif]

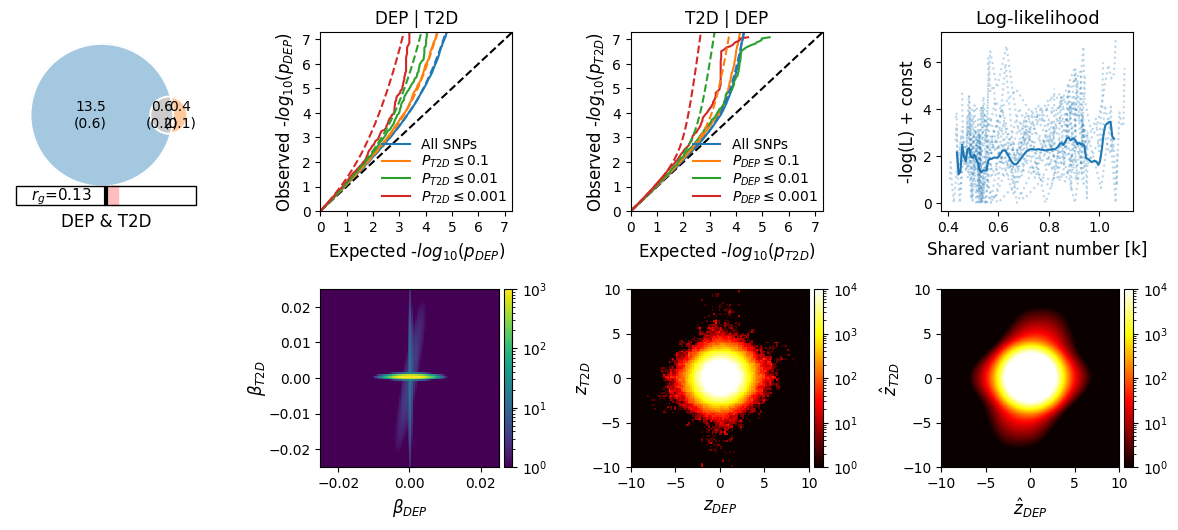

Supplement: S8 Fig — (TIF) [file pgen.1010161.s008.tif]

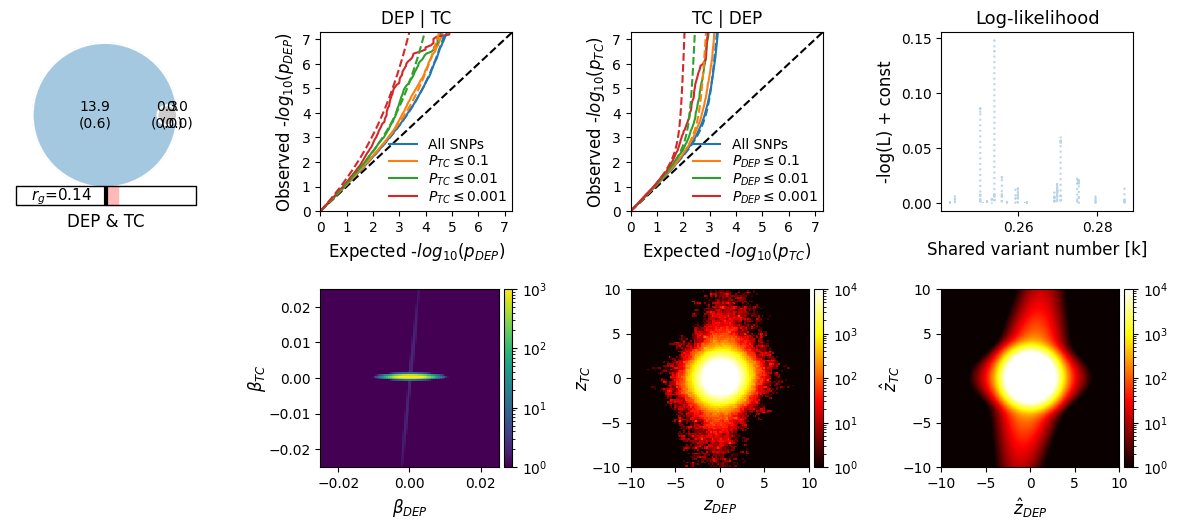

Supplement: S9 Fig — (TIF) [file pgen.1010161.s009.tif]

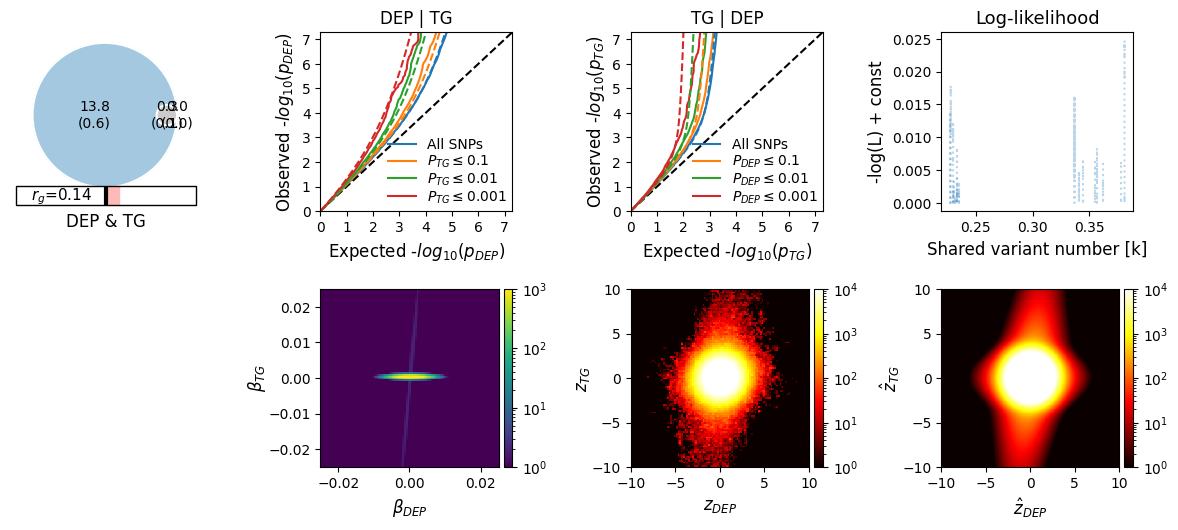

Supplement: S10 Fig — (TIF) [file pgen.1010161.s010.tif]

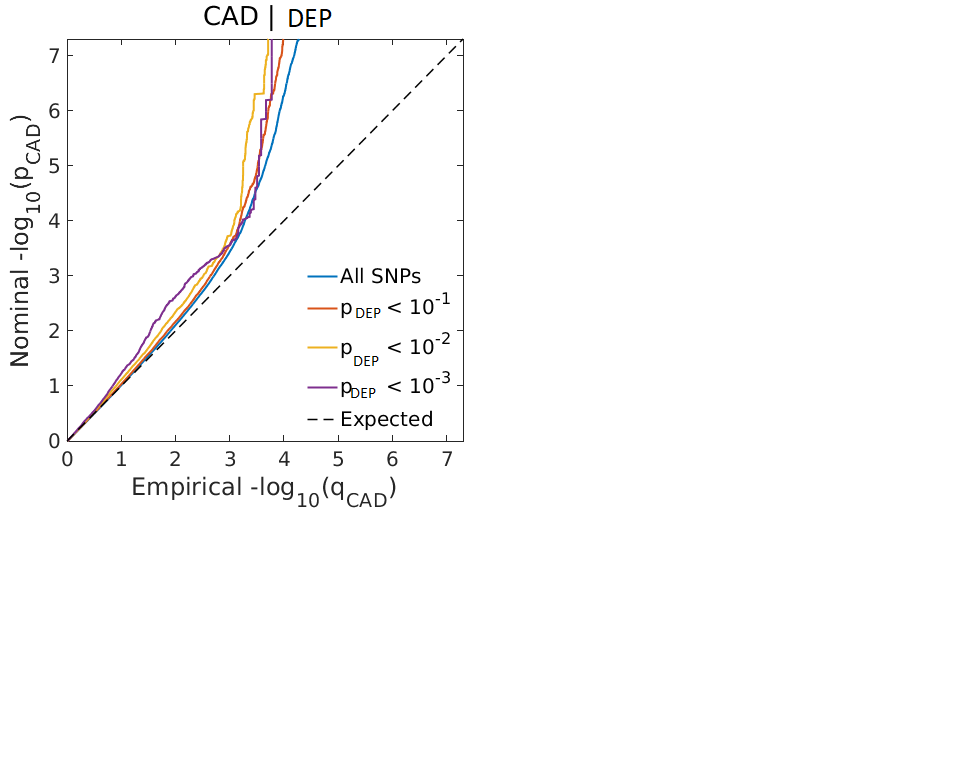

Supplement: S11 Fig — Conditional q-q plot of nominal versus empirical–log 10p values (corrected for inflation) in primary trait (CAD) below the standard GWAS threshold of p < 5 x 10^–8^ as a function of significance of association with secondary trait at the level of p< = 0.1 p < = 0.01 and p < = 0.001, respectively. The blue line indicates all SNPs. The dashed line indicate the null hypothesis. (TIF) [file pgen.1010161.s011.tif]

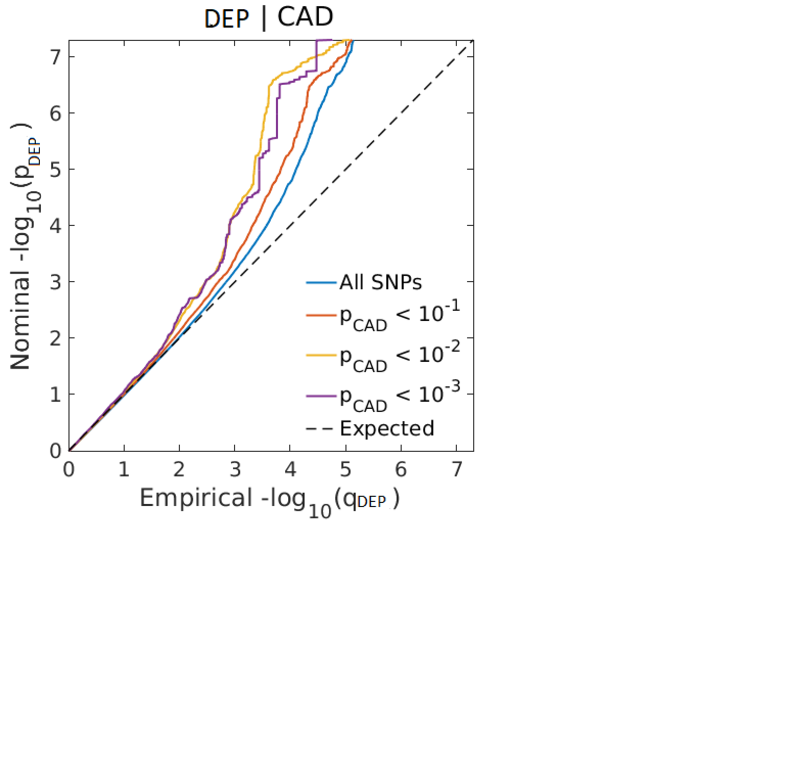

Supplement: S12 Fig — Conditional q-q plot of nominal versus empirical–log 10p values (corrected for inflation) in primary trait (DEP) below the standard GWAS threshold of p < 5 x 10^–8^ as a function of significance of association with secondary trait at the level of p< = 0.1 p < = 0.01 and p < = 0.001, respectively. The blue line indicates all SNPs. The dashed line indicate the null hypothesis. (TIF) [file pgen.1010161.s012.tif]

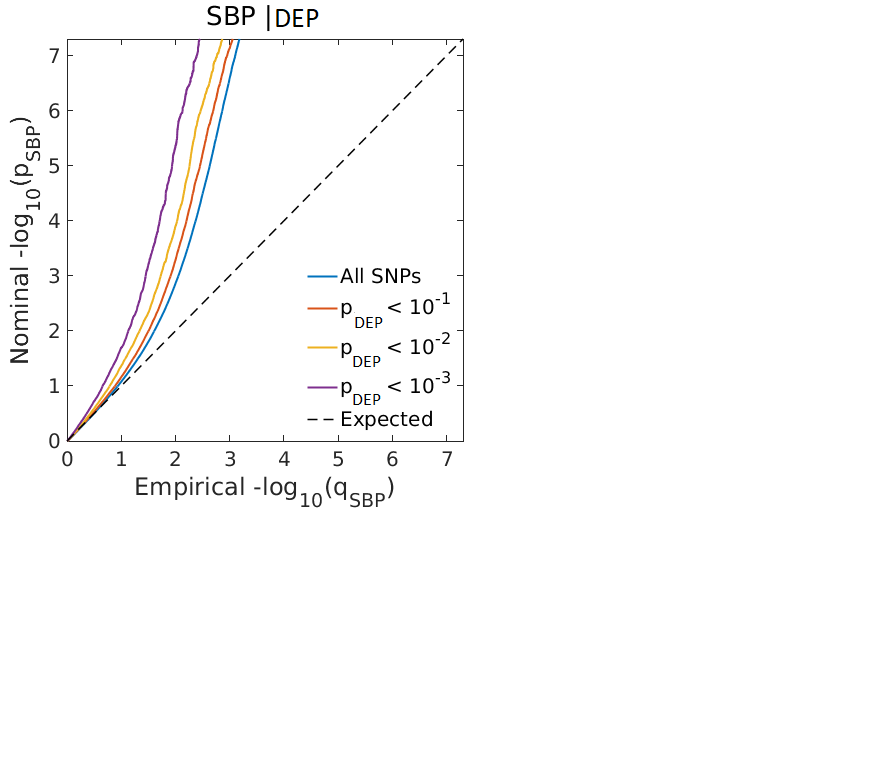

Supplement: S13 Fig — Conditional q-q plot of nominal versus empirical–log 10p values (corrected for inflation) in primary trait (SBP) below the standard GWAS threshold of p < 5 x 10^–8^ as a function of significance of association with secondary trait at the level of p< = 0.1 p < = 0.01 and p < = 0.001, respectively. The blue line indicates all SNPs. The dashed line indicate the null hypothesis. (TIF) [file pgen.1010161.s013.tif]

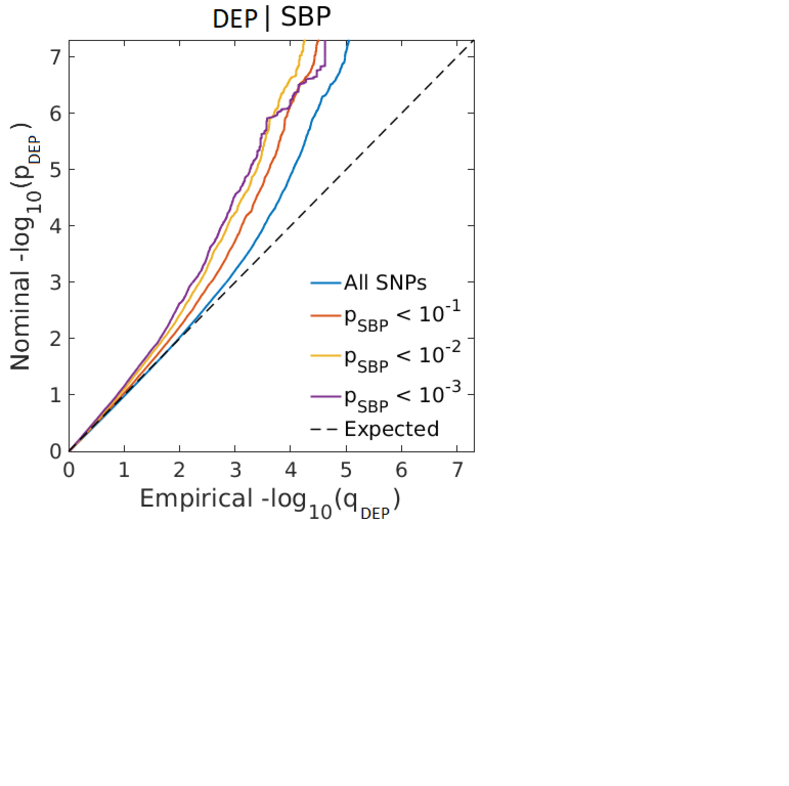

Supplement: S14 Fig — Conditional q-q plot of nominal versus empirical–log 10p values (corrected for inflation) in primary trait (DEP) below the standard GWAS threshold of p < 5 x 10^–8^ as a function of significance of association with secondary trait at the level of p< = 0.1 p < = 0.01 and p < = 0.001, respectively. The blue line indicates all SNPs. The dashed line indicate the null hypothesis. (TIF) [file pgen.1010161.s014.tif]

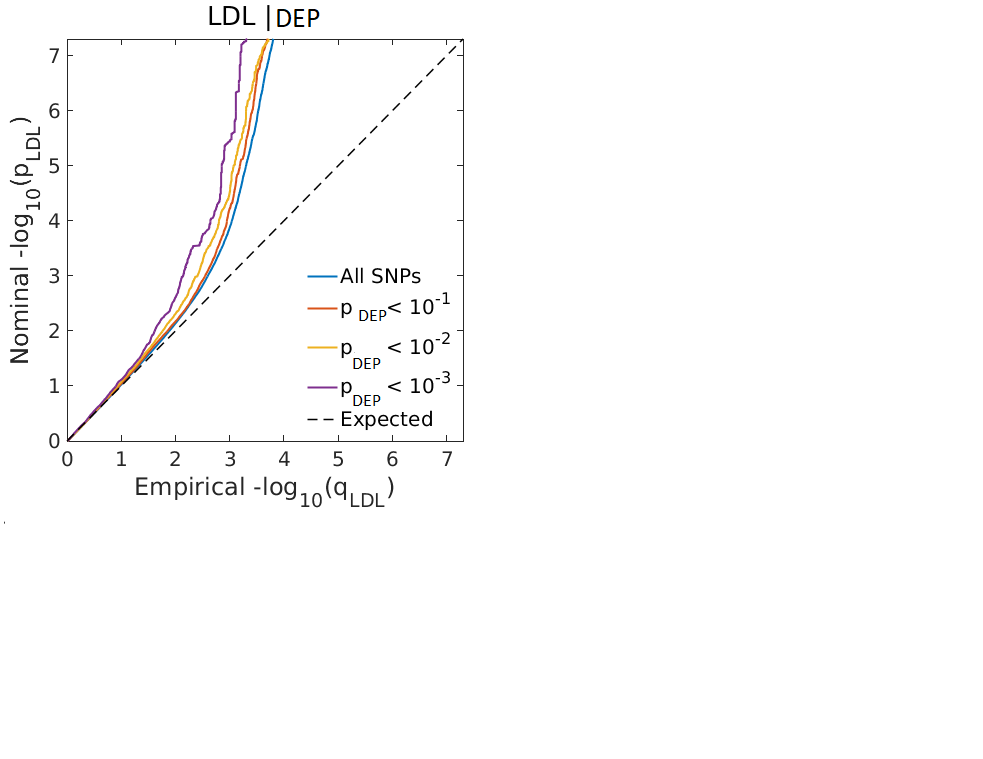

Supplement: S15 Fig — Conditional q-q plot of nominal versus empirical–log 10p values (corrected for inflation) in primary trait (LDL) below the standard GWAS threshold of p < 5 x 10^–8^ as a function of significance of association with secondary trait at the level of p< = 0.1 p < = 0.01 and p < = 0.001, respectively. The blue line indicates all SNPs. The dashed line indicate the null hypothesis. (TIF) [file pgen.1010161.s015.tif]

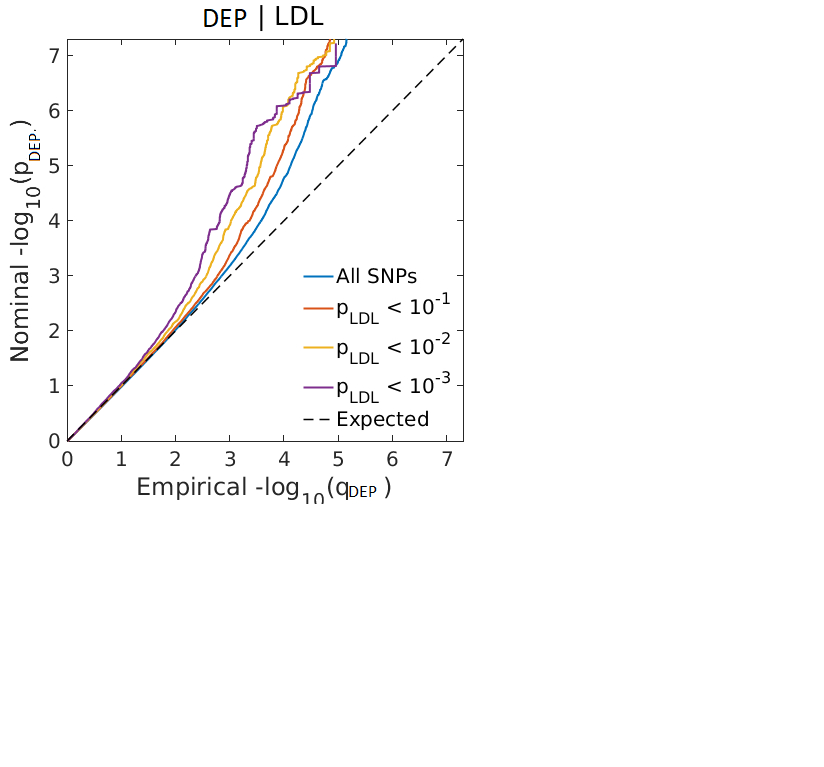

Supplement: S16 Fig — Conditional q-q plot of nominal versus empirical–log 10p values (corrected for inflation) in primary trait (DEP) below the standard GWAS threshold of p < 5 x 10^–8^ as a function of significance of association with secondary trait at the level of p< = 0.1 p < = 0.01 and p < = 0.001, respectively. The blue line indicates all SNPs. The dashed line indicate the null hypothesis. (TIF) [file pgen.1010161.s016.tif]

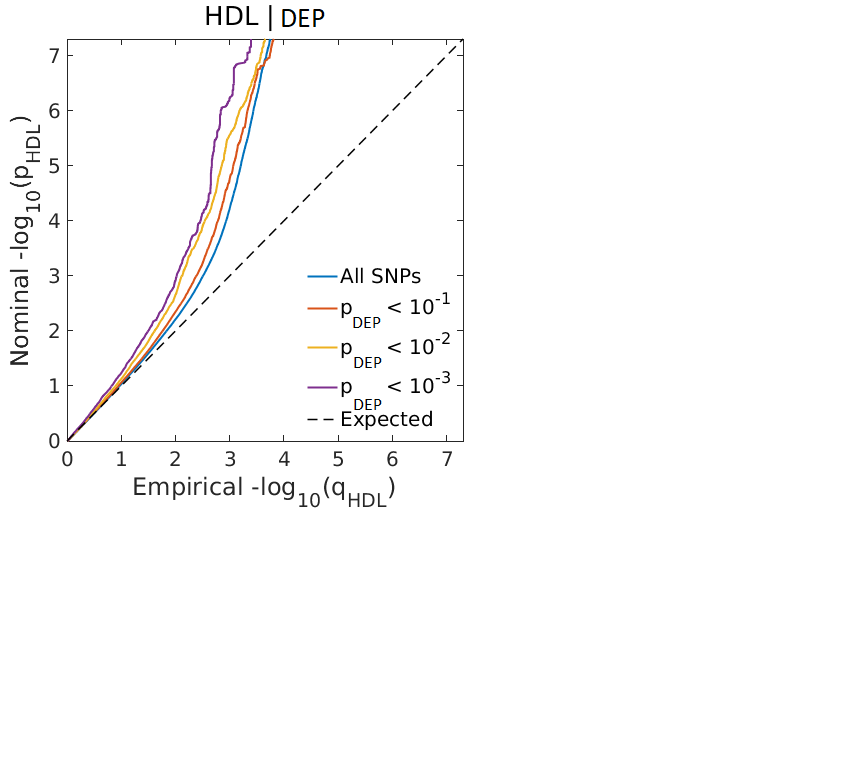

Supplement: S17 Fig — Conditional q-q plot of nominal versus empirical–log 10p values (corrected for inflation) in primary trait (HDL) below the standard GWAS threshold of p < 5 x 10^–8^ as a function of significance of association with secondary trait at the level of p< = 0.1 p < = 0.01 and p < = 0.001, respectively. The blue line indicates all SNPs. The dashed line indicate the null hypothesis. (TIF) [file pgen.1010161.s017.tif]

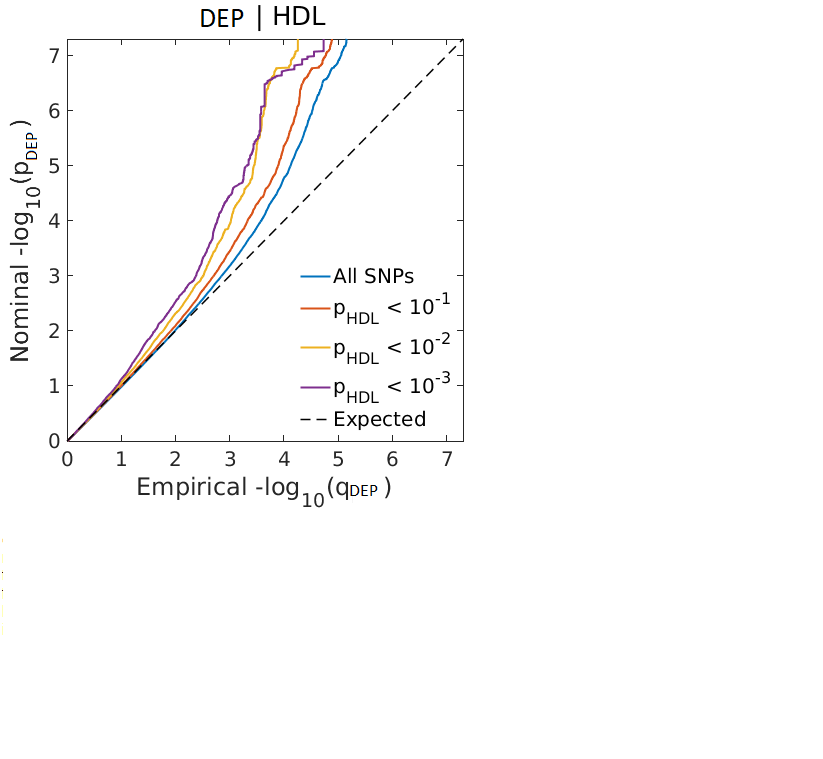

Supplement: S18 Fig — Conditional q-q plot of nominal versus empirical–log 10p values (corrected for inflation) in primary trait (DEP) below the standard GWAS threshold of p < 5 x 10^–8^ as a function of significance of association with secondary trait at the level of p< = 0.1 p < = 0.01 and p < = 0.001, respectively. The blue line indicates all SNPs. The dashed line indicate the null hypothesis. (TIF) [file pgen.1010161.s018.tif]

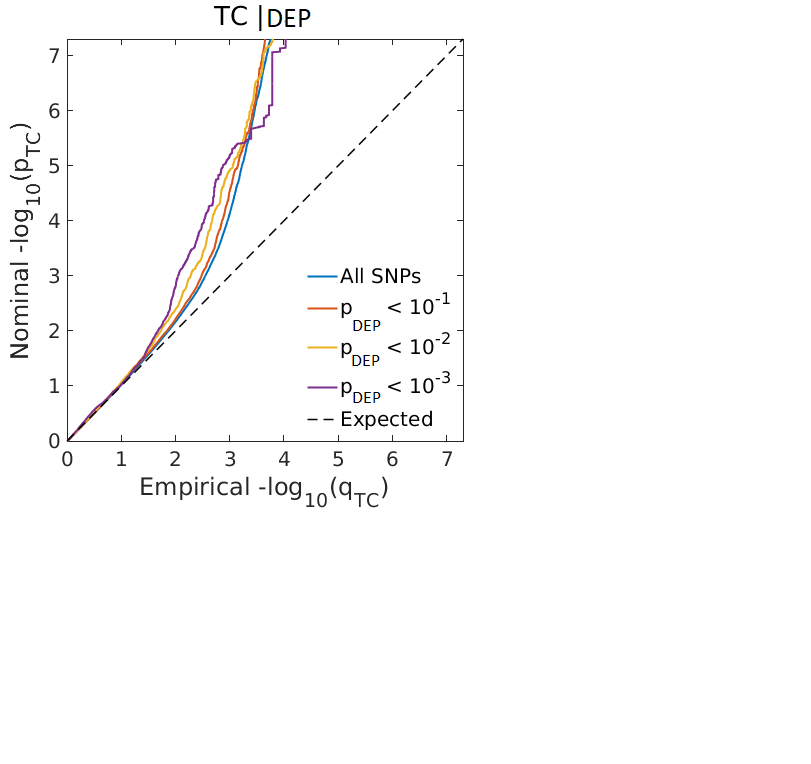

Supplement: S19 Fig — Conditional q-q plot of nominal versus empirical–log 10p values (corrected for inflation) in primary trait (TC) below the standard GWAS threshold of p < 5 x 10^–8^ as a function of significance of association with secondary trait at the level of p< = 0.1 p < = 0.01 and p < = 0.001, respectively. The blue line indicates all SNPs. The dashed line indicate the null hypothesis. (TIF) [file pgen.1010161.s019.tif]

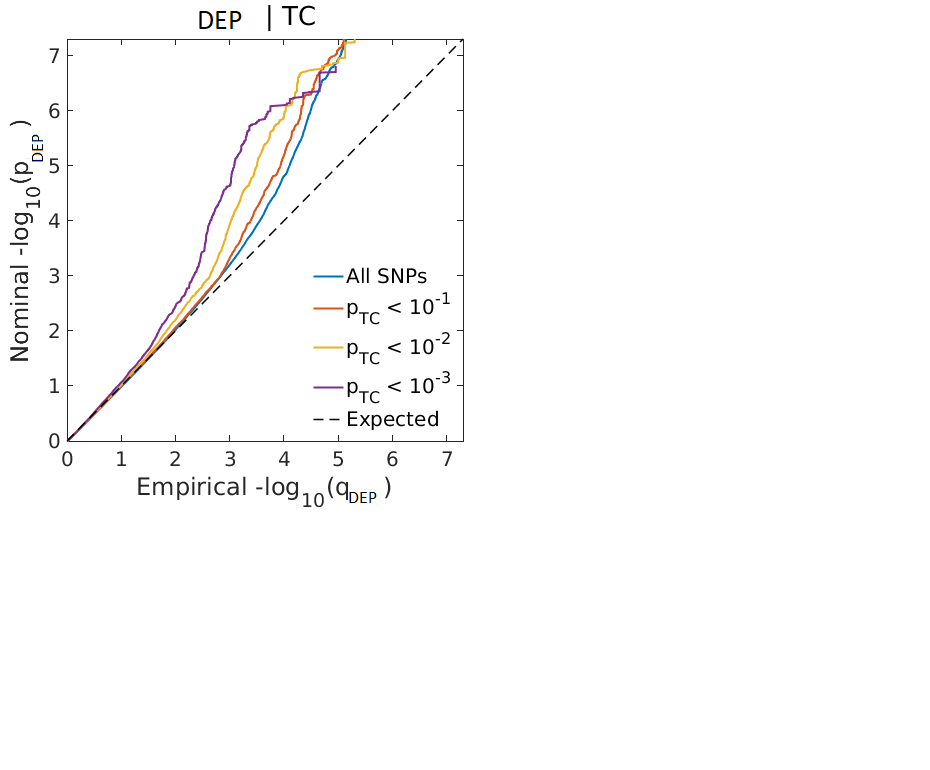

Supplement: S20 Fig — Conditional q-q plot of nominal versus empirical–log 10p values (corrected for inflation) in primary trait (DEP) below the standard GWAS threshold of p < 5 x 10^–8^ as a function of significance of association with secondary trait at the level of p< = 0.1 p < = 0.01 and p < = 0.001, respectively. The blue line indicates all SNPs. The dashed line indicate the null hypothesis. (TIF) [file pgen.1010161.s020.tif]

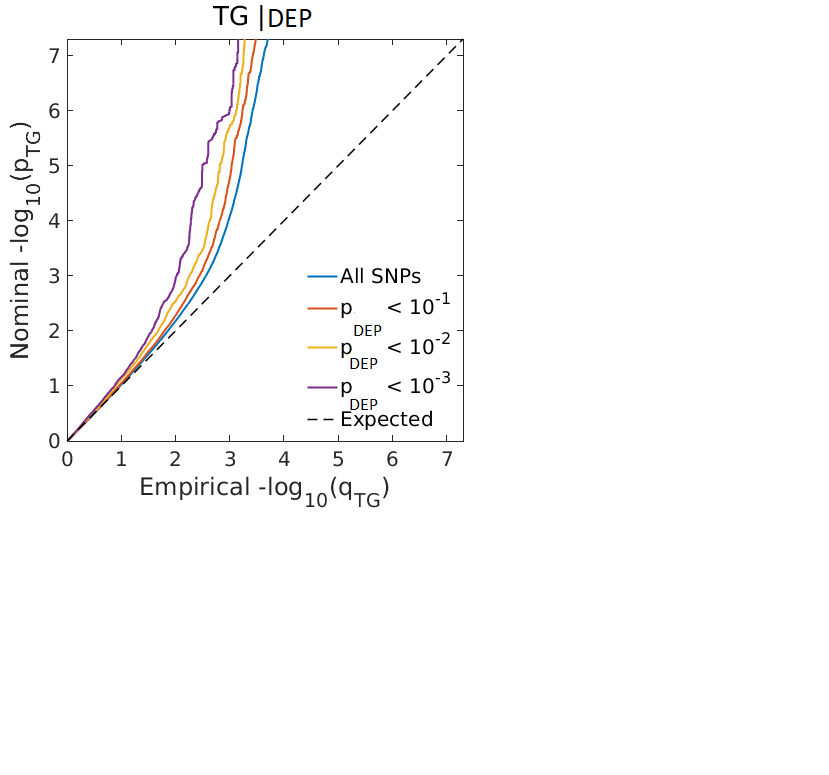

Supplement: S21 Fig — Conditional q-q plot of nominal versus empirical–log 10p values (corrected for inflation) in primary trait (TG) below the standard GWAS threshold of p < 5 x 10^–8^ as a function of significance of association with secondary trait at the level of p< = 0.1 p < = 0.01 and p < = 0.001, respectively. The blue line indicates all SNPs. The dashed line indicate the null hypothesis. (TIF) [file pgen.1010161.s021.tif]

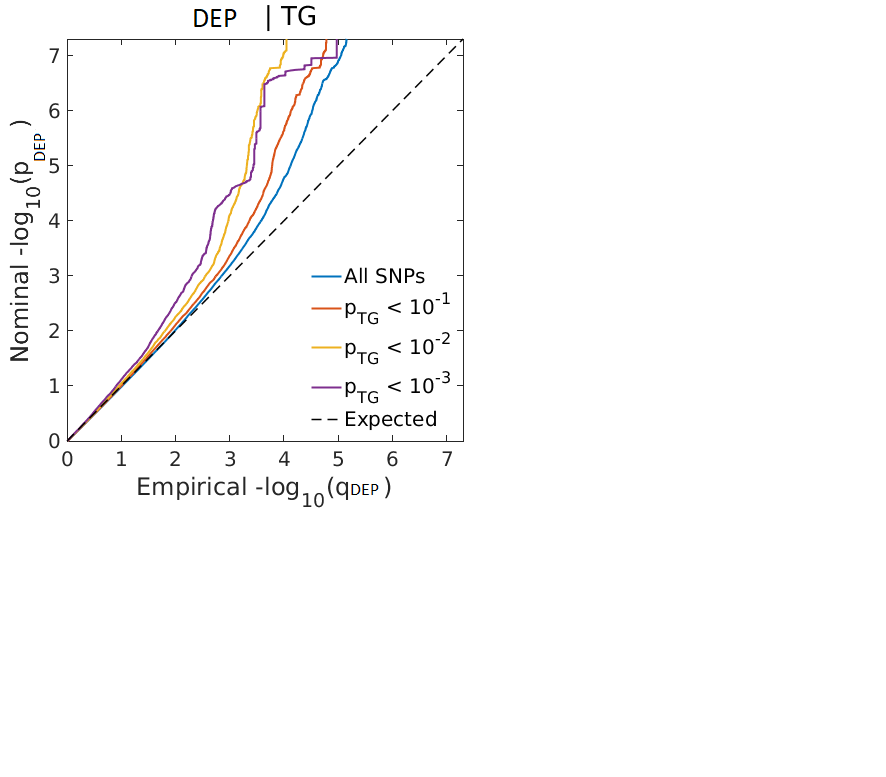

Supplement: S22 Fig — Conditional q-q plot of nominal versus empirical–log 10p values (corrected for inflation) in primary trait (DEP) below the standard GWAS threshold of p < 5 x 10^–8^ as a function of significance of association with secondary trait at the level of p< = 0.1 p < = 0.01 and p < = 0.001, respectively. The blue line indicates all SNPs. The dashed line indicate the null hypothesis. (TIF) [file pgen.1010161.s022.tif]

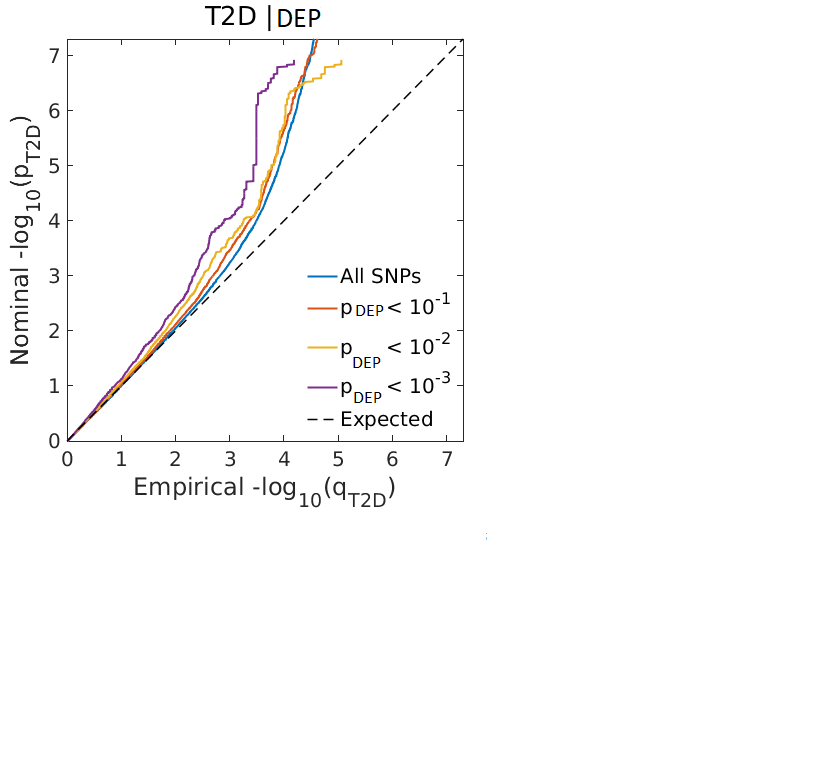

Supplement: S23 Fig — Conditional q-q plot of nominal versus empirical–log 10p values (corrected for inflation) in primary trait (DBP) below the standard GWAS threshold of p < 5 x 10^–8^ as a function of significance of association with secondary trait at the level of p< = 0.1 p < = 0.01 and p < = 0.001, respectively. The blue line indicates all SNPs. The dashed line indicate the null hypothesis. (TIF) [file pgen.1010161.s023.tif]

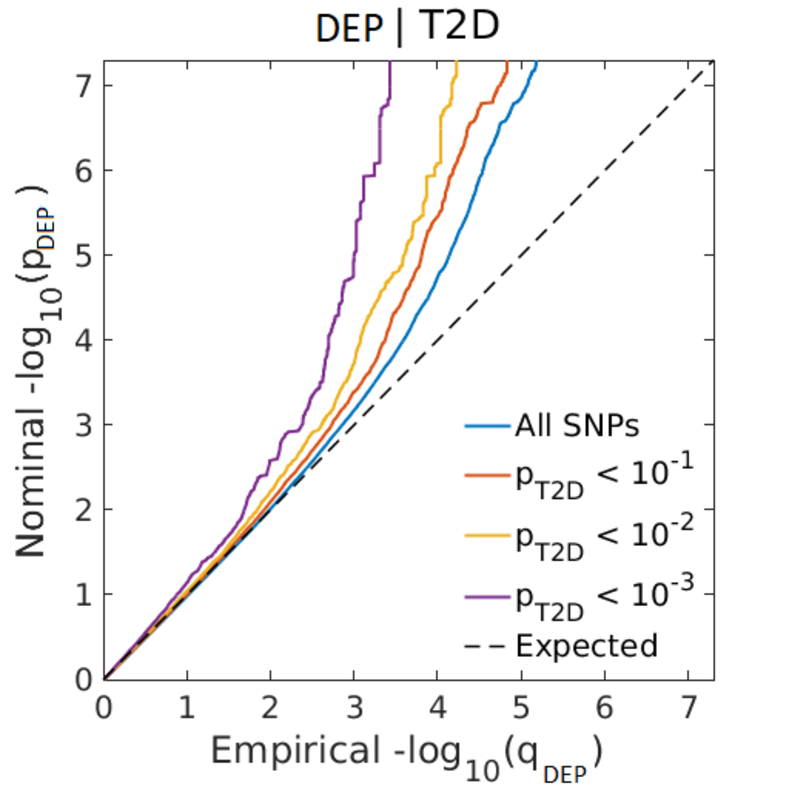

Supplement: S24 Fig — Conditional q-q plot of nominal versus empirical–log 10p values (corrected for inflation) in primary trait (DEP) below the standard GWAS threshold of p < 5 x 10^–8^ as a function of significance of association with secondary trait at the level of p< = 0.1 p < = 0.01 and p < = 0.001, respectively. The blue line indicates all SNPs. The dashed line indicate the null hypothesis. (TIF) [file pgen.1010161.s024.tif]

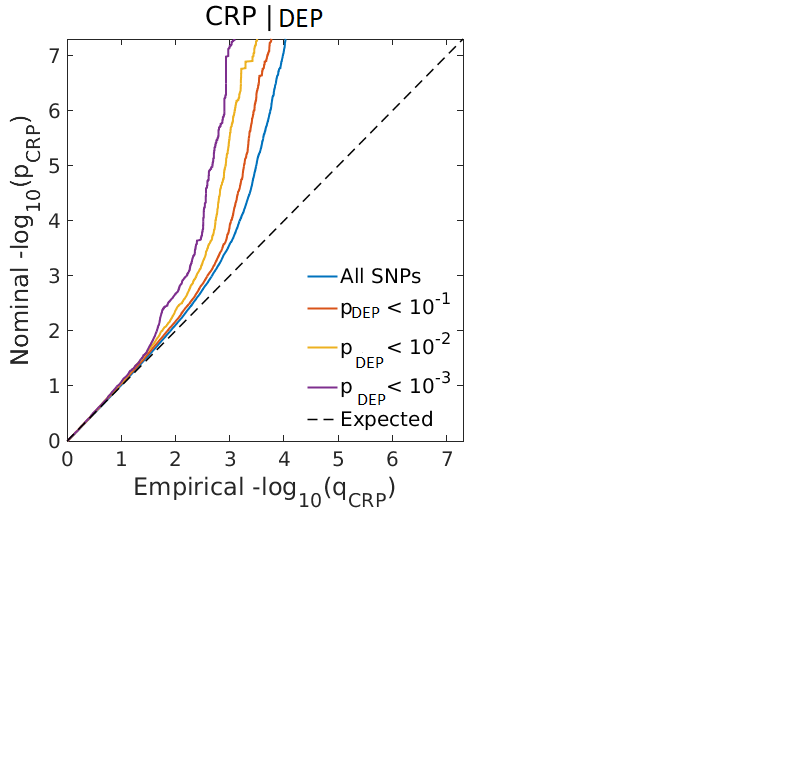

Supplement: S25 Fig — Conditional q-q plot of nominal versus empirical–log 10p values (corrected for inflation) in primary trait (CRP) below the standard GWAS threshold of p < 5 x 10^–8^ as a function of significance of association with secondary trait at the level of p< = 0.1 p < = 0.01 and p < = 0.001, respectively. The blue line indicates all SNPs. The dashed line indicate the null hypothesis. (TIF) [file pgen.1010161.s025.tif]

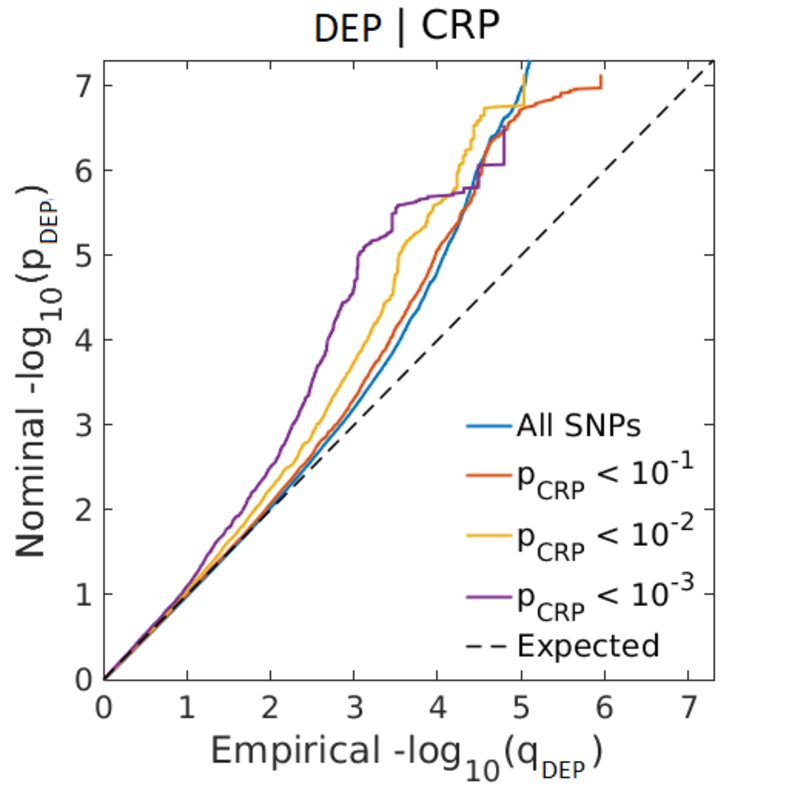

Supplement: S26 Fig — Conditional q-q plot of nominal versus empirical–log 10p values (corrected for inflation) in primary trait (DEP) below the standard GWAS threshold of p < 5 x 10^–8^ as a function of significance of association with secondary trait at the level of p< = 0.1 p < = 0.01 and p < = 0.001, respectively. The blue line indicates all SNPs. The dashed line indicate the null hypothesis. (TIF) [file pgen.1010161.s026.tif]

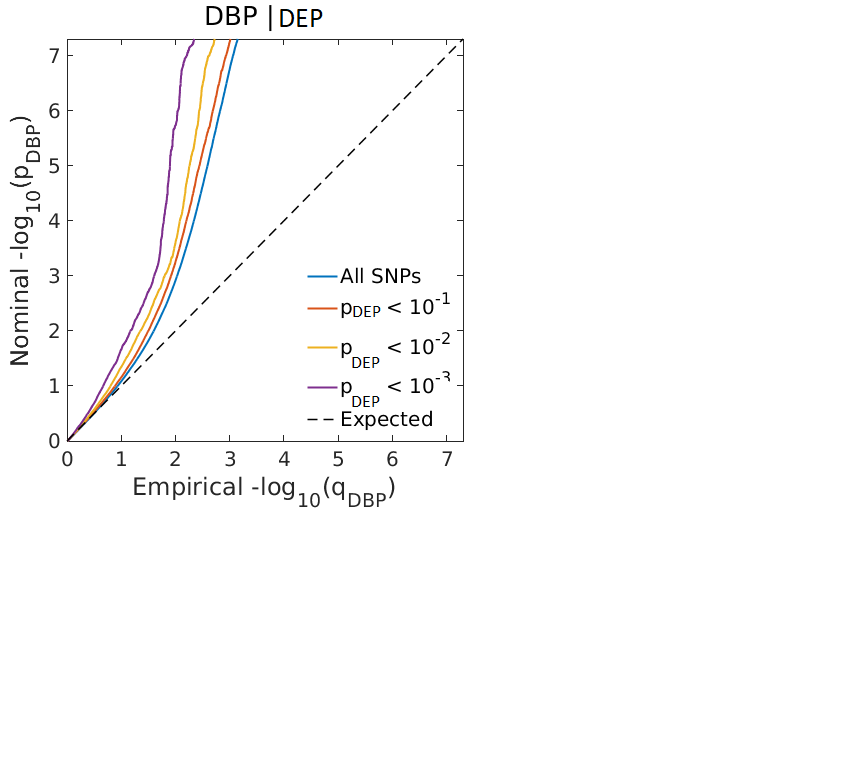

Supplement: S27 Fig — Conditional q-q plot of nominal versus empirical–log 10p values (corrected for inflation) in primary trait (DBP) below the standard GWAS threshold of p < 5 x 10^–8^ as a function of significance of association with secondary trait at the level of p< = 0.1 p < = 0.01 and p < = 0.001, respectively. The blue line indicates all SNPs. The dashed line indicate the null hypothesis. (TIF) [file pgen.1010161.s027.tif]

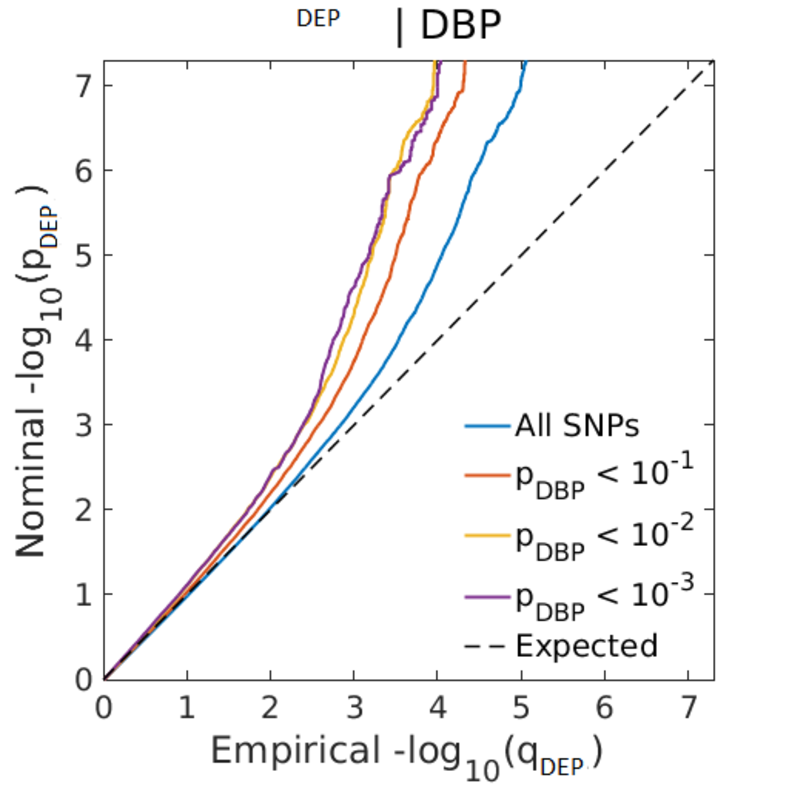

Supplement: S28 Fig — Conditional q-q plot of nominal versus empirical–log 10p values (corrected for inflation) in primary trait (DEP) below the standard GWAS threshold of p < 5 x 10^–8^ as a function of significance of association with secondary trait at the level of p< = 0.1 p < = 0.01 and p < = 0.001, respectively. The blue line indicates all SNPs. The dashed line indicate the null hypothesis. (TIF) [file pgen.1010161.s028.tif]

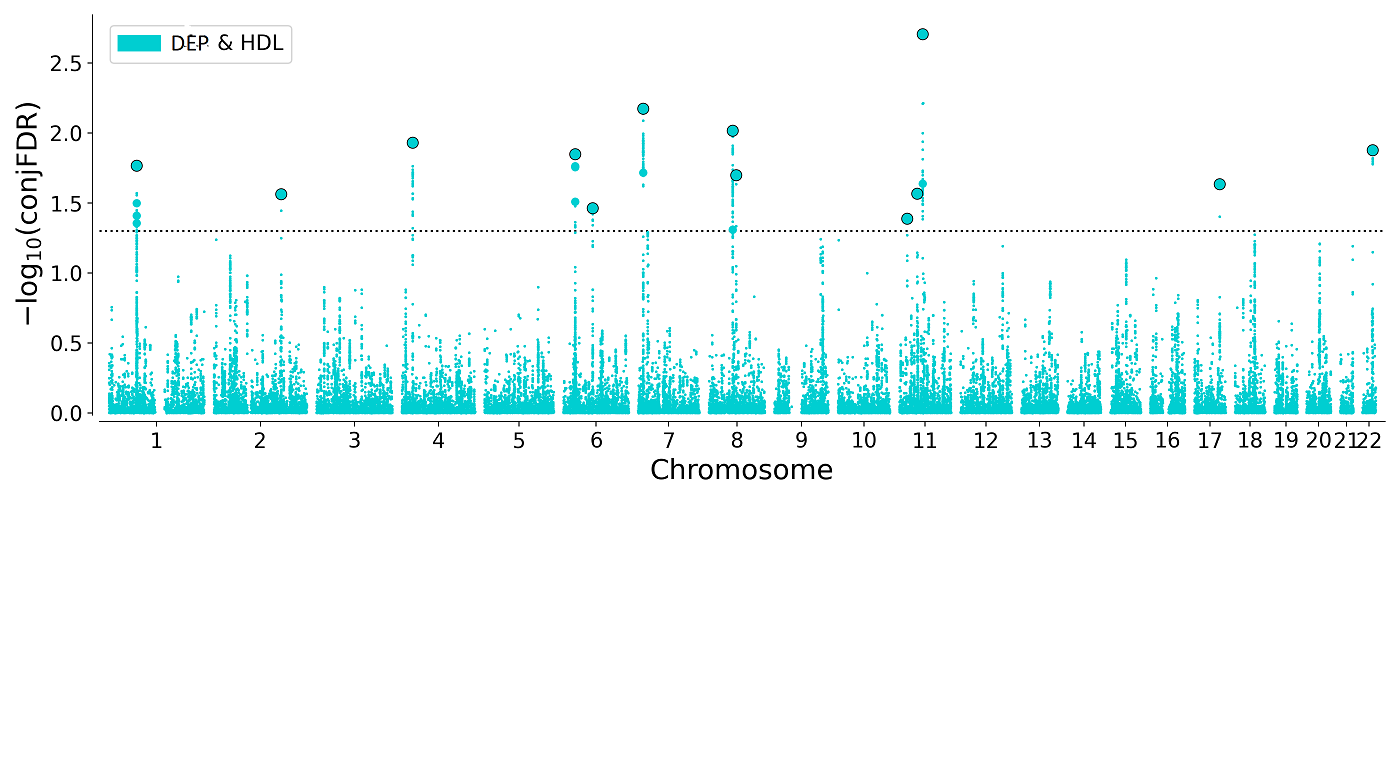

Supplement: S29 Fig — Common genetic variants both associated with depression (DEP) and high-density lipoprotein (HDL) at conjunctional false discovery rate (conjFDR) < 0.05. Manhattan plot showing the–log10 transformed conjFDR values for each SNP on the y axis and the chromosomal positions along the x axis. The dotted horizontal line represents the threshold for significant shared associations (conjFDR < 0.05, ie., -log10(conjFDR) > 2.0). Independent lead SNPs are encircled in black. The significant shared signal in the major histocompatibility complex region (chr6:25119106–33854733) is represented by one independent lead SNP. (TIF) [file pgen.1010161.s029.tif]

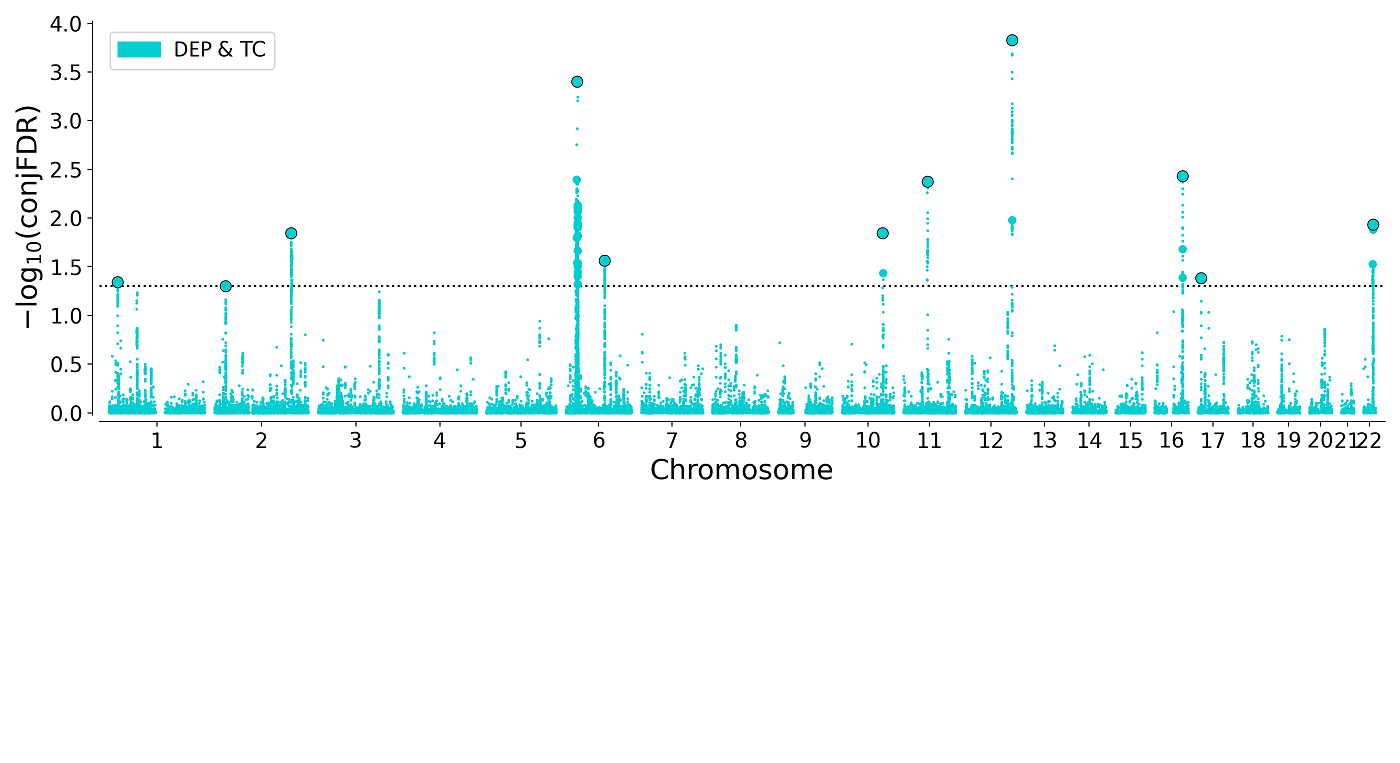

Supplement: S30 Fig — Common genetic variants both associated with depression (DEP) and high-density lipoprotein (HDL) at conjunctional false discovery rate (conjFDR) < 0.05. Manhattan plot showing the–log10 transformed conjFDR values for each SNP on the y axis and the chromosomal positions along the x axis. The dotted horizontal line represents the threshold for significant shared associations (conjFDR < 0.05, ie., -log10(conjFDR) > 2.0). Independent lead SNPs are encircled in black. The significant shared signal in the major histocompatibility complex region (chr6:25119106–33854733) is represented by one independent lead SNP. (TIF) [file pgen.1010161.s030.tif]

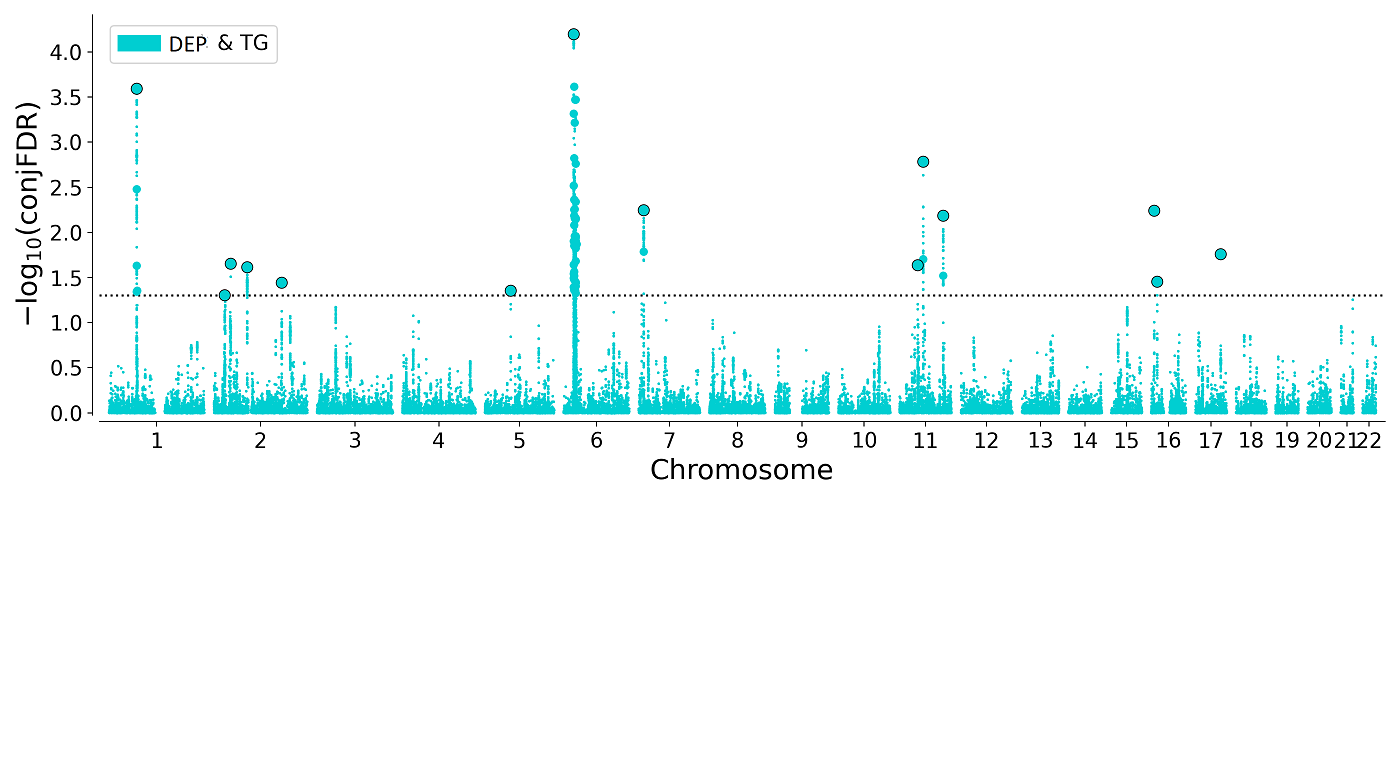

Supplement: S31 Fig — Common genetic variants both associated with depression (DEP) and high-density lipoprotein (HDL) at conjunctional false discovery rate (conjFDR) < 0.05. Manhattan plot showing the–log10 transformed conjFDR values for each SNP on the y axis and the chromosomal positions along the x axis. The dotted horizontal line represents the threshold for significant shared associations (conjFDR < 0.05, ie., -log10(conjFDR) > 2.0). Independent lead SNPs are encircled in black. The significant shared signal in the major histocompatibility complex region (chr6:25119106–33854733) is represented by one independent lead SNP. (TIF) [file pgen.1010161.s031.tif]

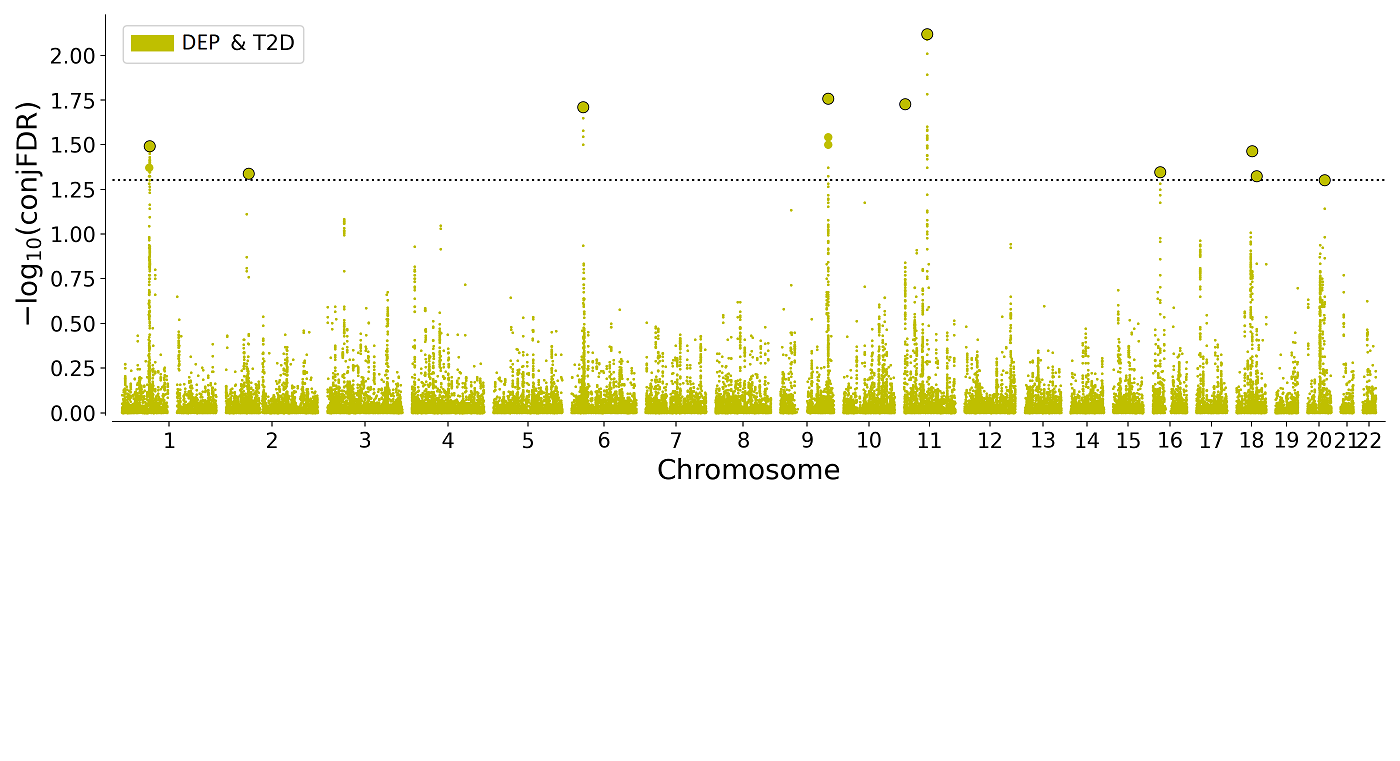

Supplement: S32 Fig — Common genetic variants both associated with depression (DEP) and high-density lipoprotein (HDL) at conjunctional false discovery rate (conjFDR) < 0.05. Manhattan plot showing the–log10 transformed conjFDR values for each SNP on the y axis and the chromosomal positions along the x axis. The dotted horizontal line represents the threshold for significant shared associations (conjFDR < 0.05, ie., -log10(conjFDR) > 2.0). Independent lead SNPs are encircled in black. The significant shared signal in the major histocompatibility complex region (chr6:25119106–33854733) is represented by one independent lead SNP. (TIF) [file pgen.1010161.s032.tif]

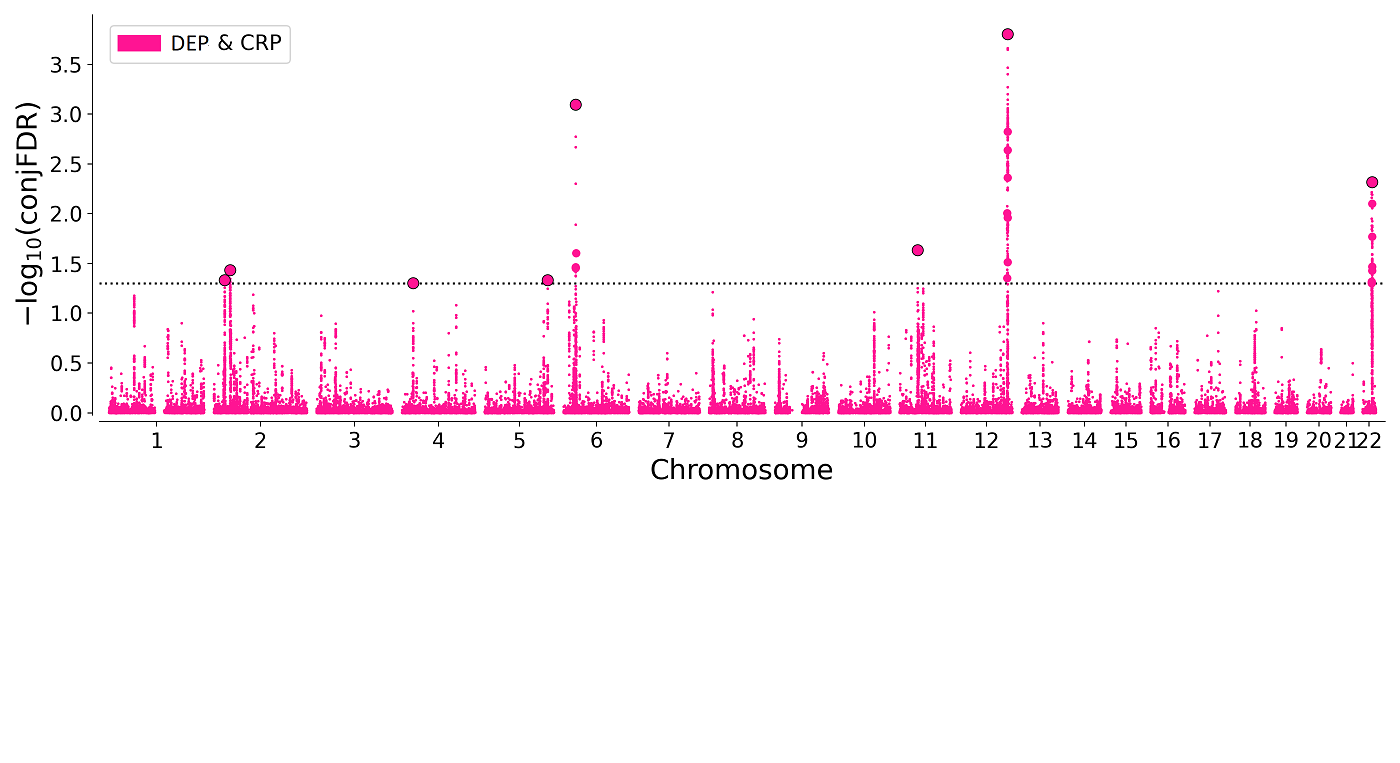

Supplement: S33 Fig — Common genetic variants both associated with depression (DEP) and high-density lipoprotein (HDL) at conjunctional false discovery rate (conjFDR) < 0.05. Manhattan plot showing the–log10 transformed conjFDR values for each SNP on the y axis and the chromosomal positions along the x axis. The dotted horizontal line represents the threshold for significant shared associations (conjFDR < 0.05, ie., -log10(conjFDR) > 2.0). Independent lead SNPs are encircled in black. The significant shared signal in the major histocompatibility complex region (chr6:25119106–33854733) is represented by one independent lead SNP. (TIF) [file pgen.1010161.s033.tif]

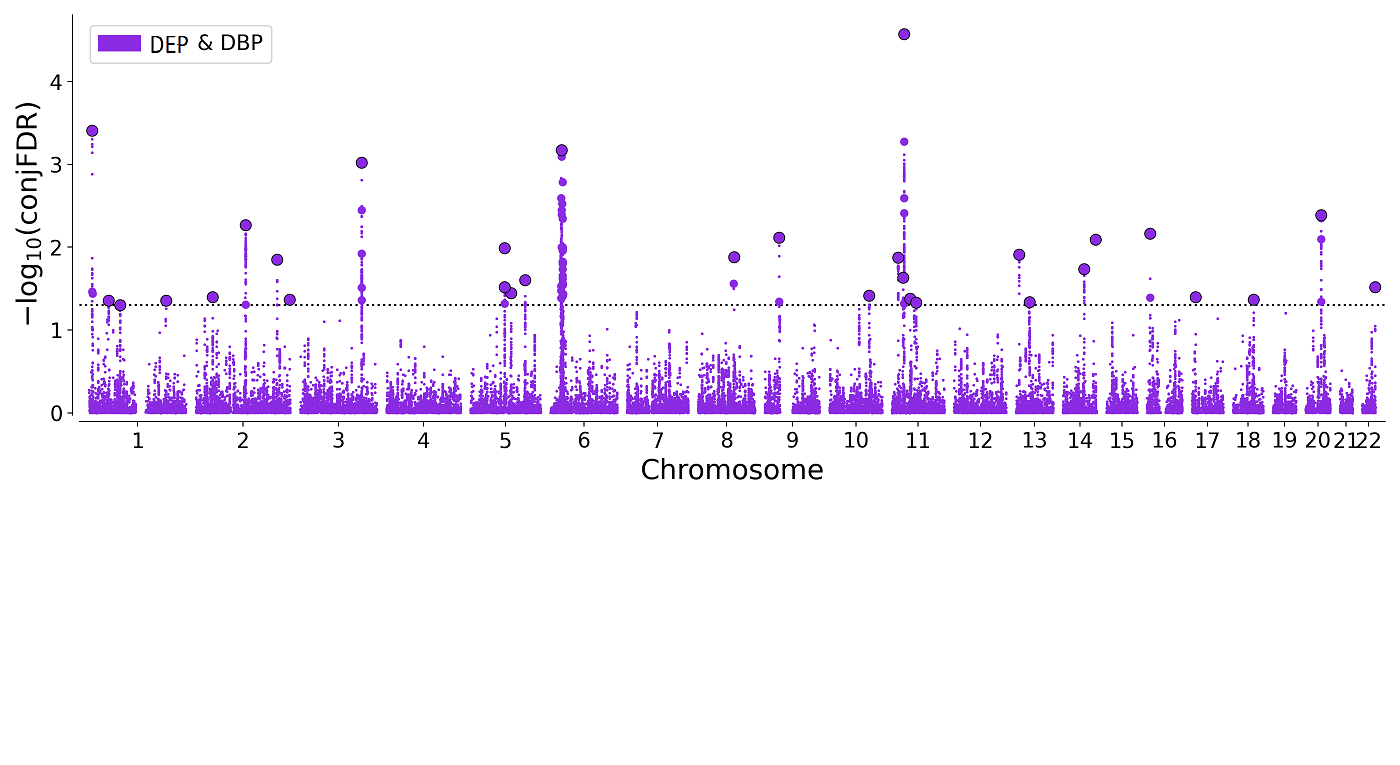

Supplement: S34 Fig — Common genetic variants both associated with depression (DEP) and high-density lipoprotein (HDL) at conjunctional false discovery rate (conjFDR) < 0.05. Manhattan plot showing the–log10 transformed conjFDR values for each SNP on the y axis and the chromosomal positions along the x axis. The dotted horizontal line represents the threshold for significant shared associations (conjFDR < 0.05, ie., -log10(conjFDR) > 2.0). Independent lead SNPs are encircled in black. The significant shared signal in the major histocompatibility complex region (chr6:25119106–33854733) is represented by one independent lead SNP. (TIF) [file pgen.1010161.s034.tif]

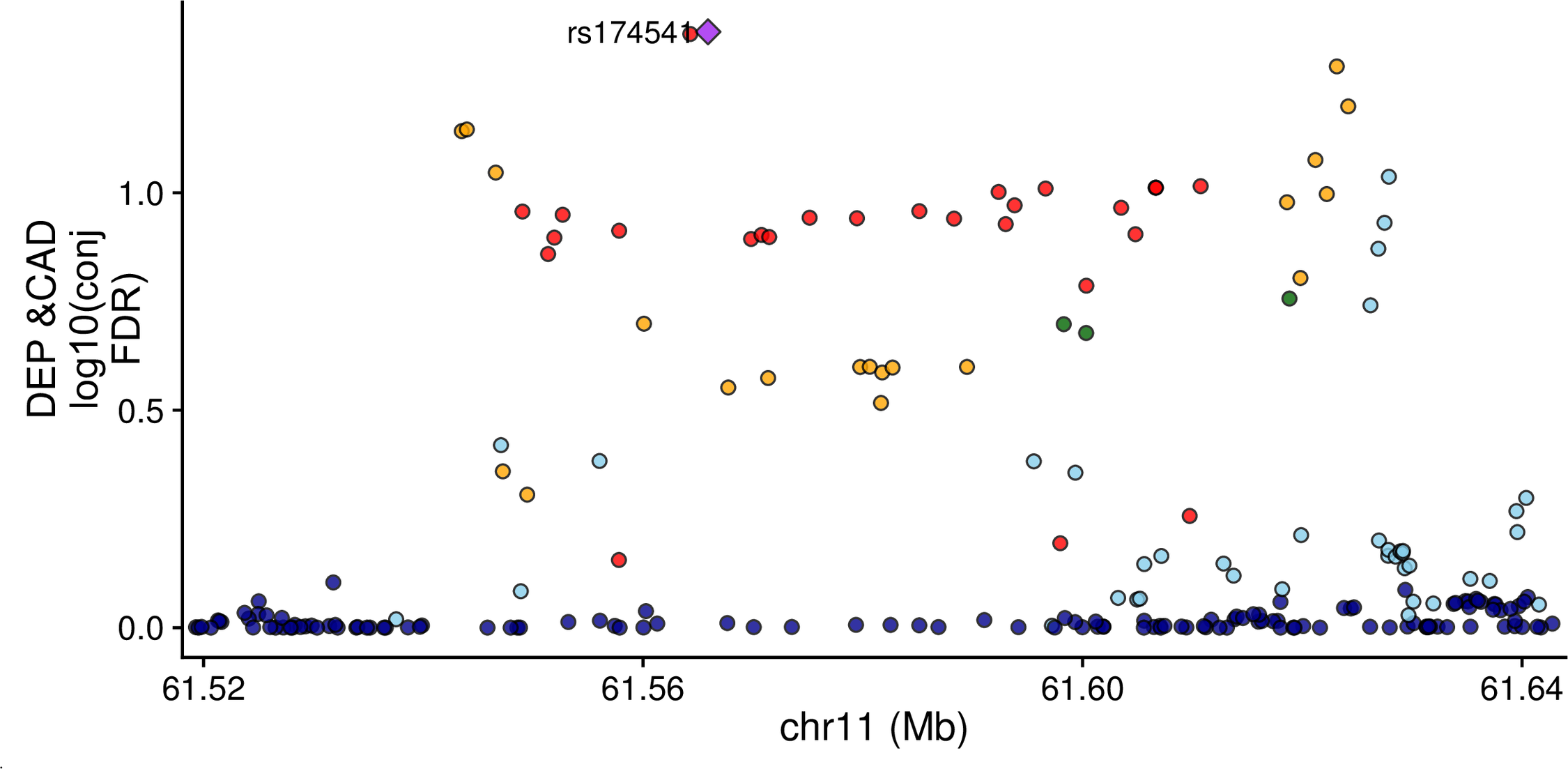

Supplement: S35 Fig — (TIF) [file pgen.1010161.s035.tif]

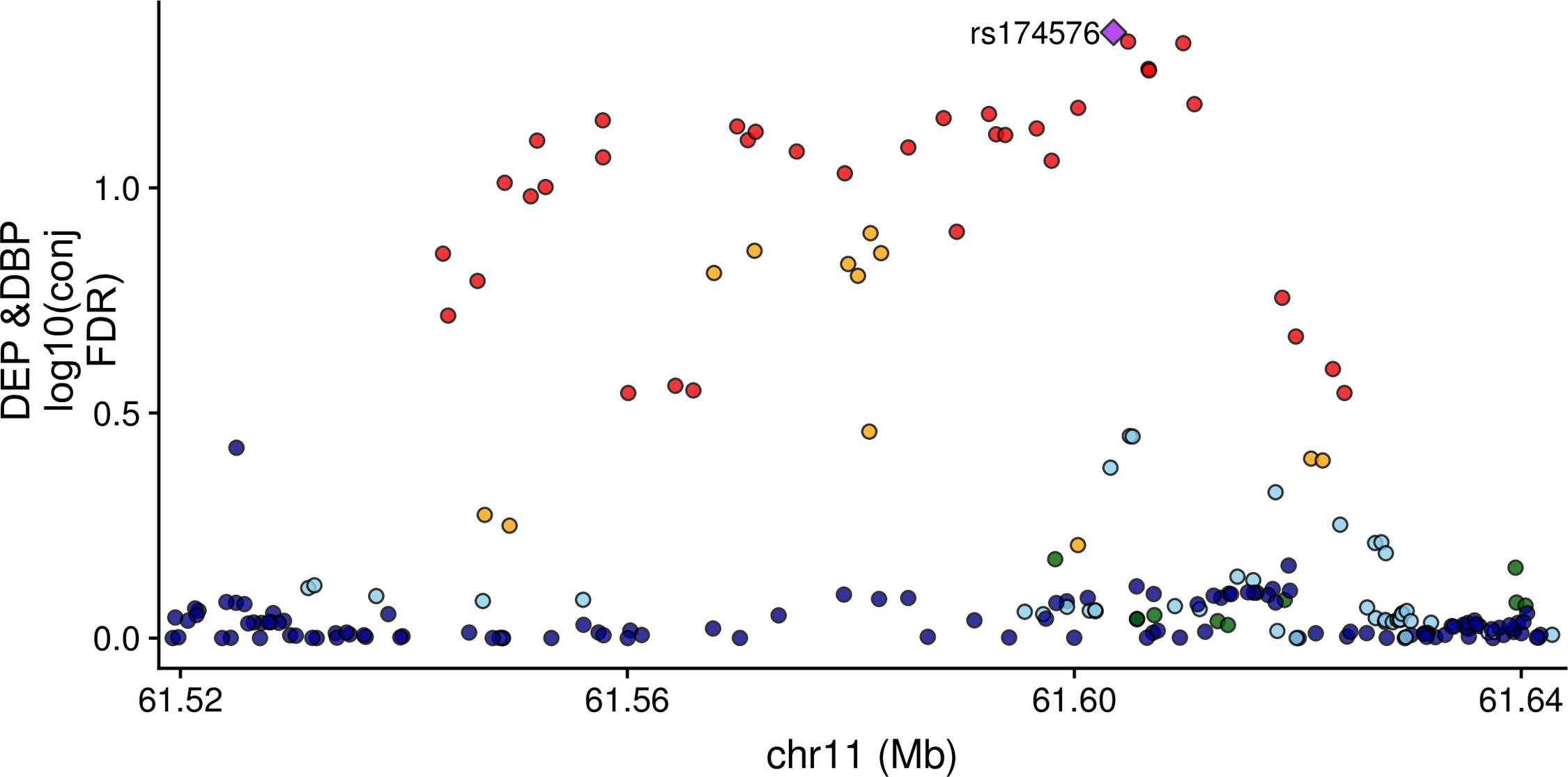

Supplement: S36 Fig — (TIF) [file pgen.1010161.s036.tif]

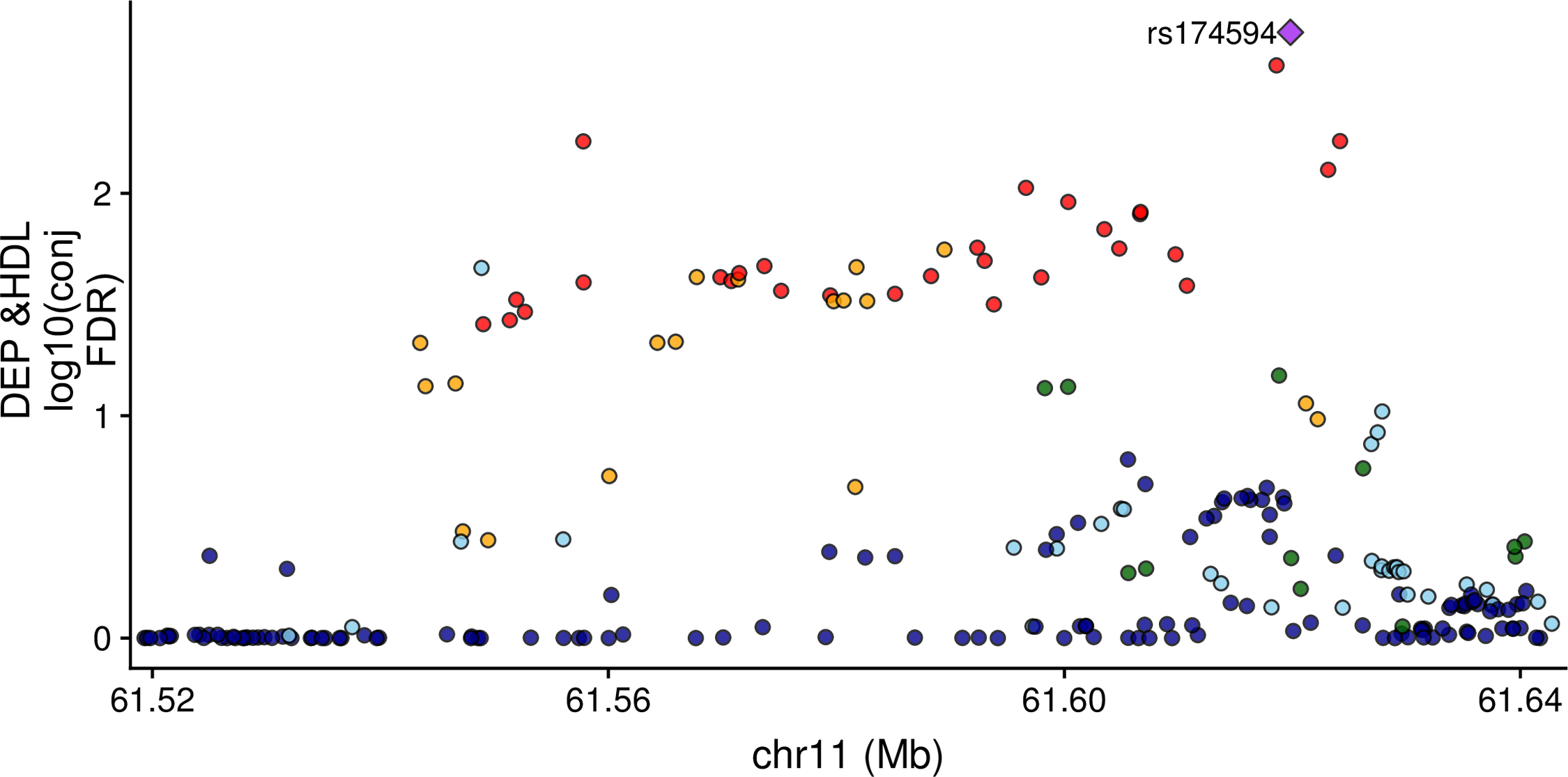

Supplement: S37 Fig — This leadSNP was in common for depression, HDL, LDL, TC and TG. (TIF) [file pgen.1010161.s037.tif]

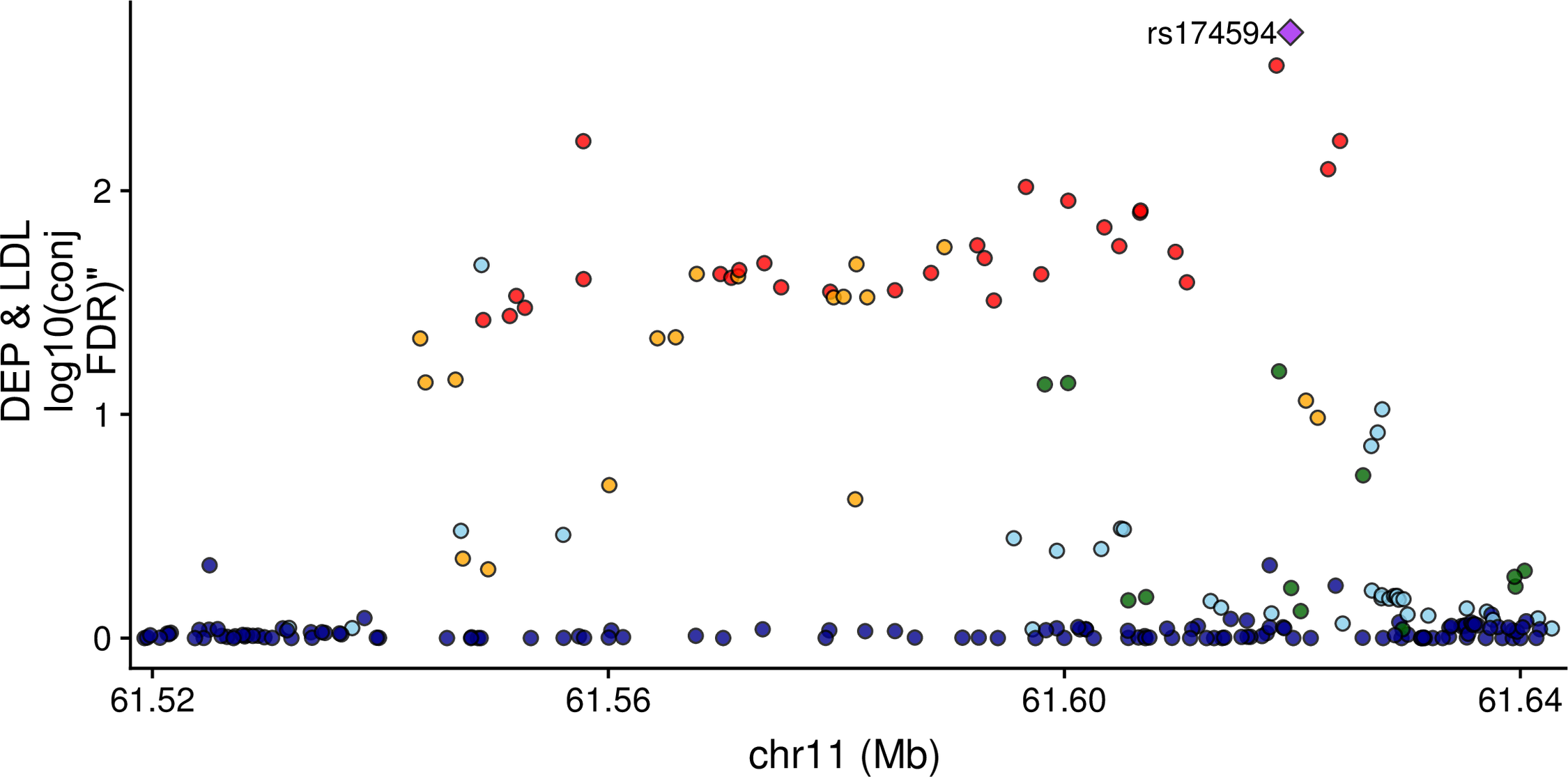

Supplement: S38 Fig — This leadSNP was in common for depression, HDL, LDL, TC and TG. (TIF) [file pgen.1010161.s038.tif]

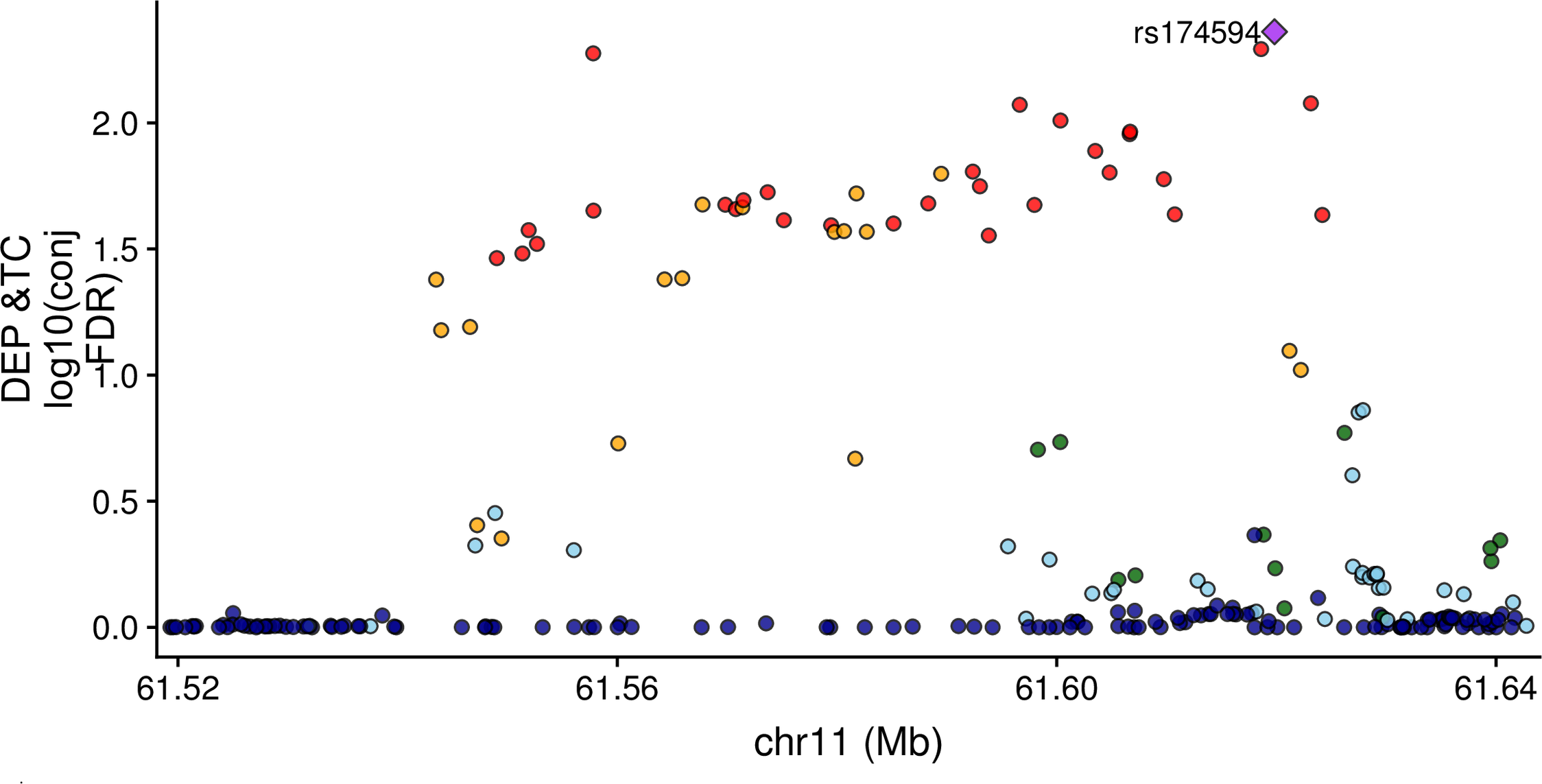

Supplement: S39 Fig — This leadSNP was in common for depression, HDL, LDL, TC and TG. (TIF) [file pgen.1010161.s039.tif]

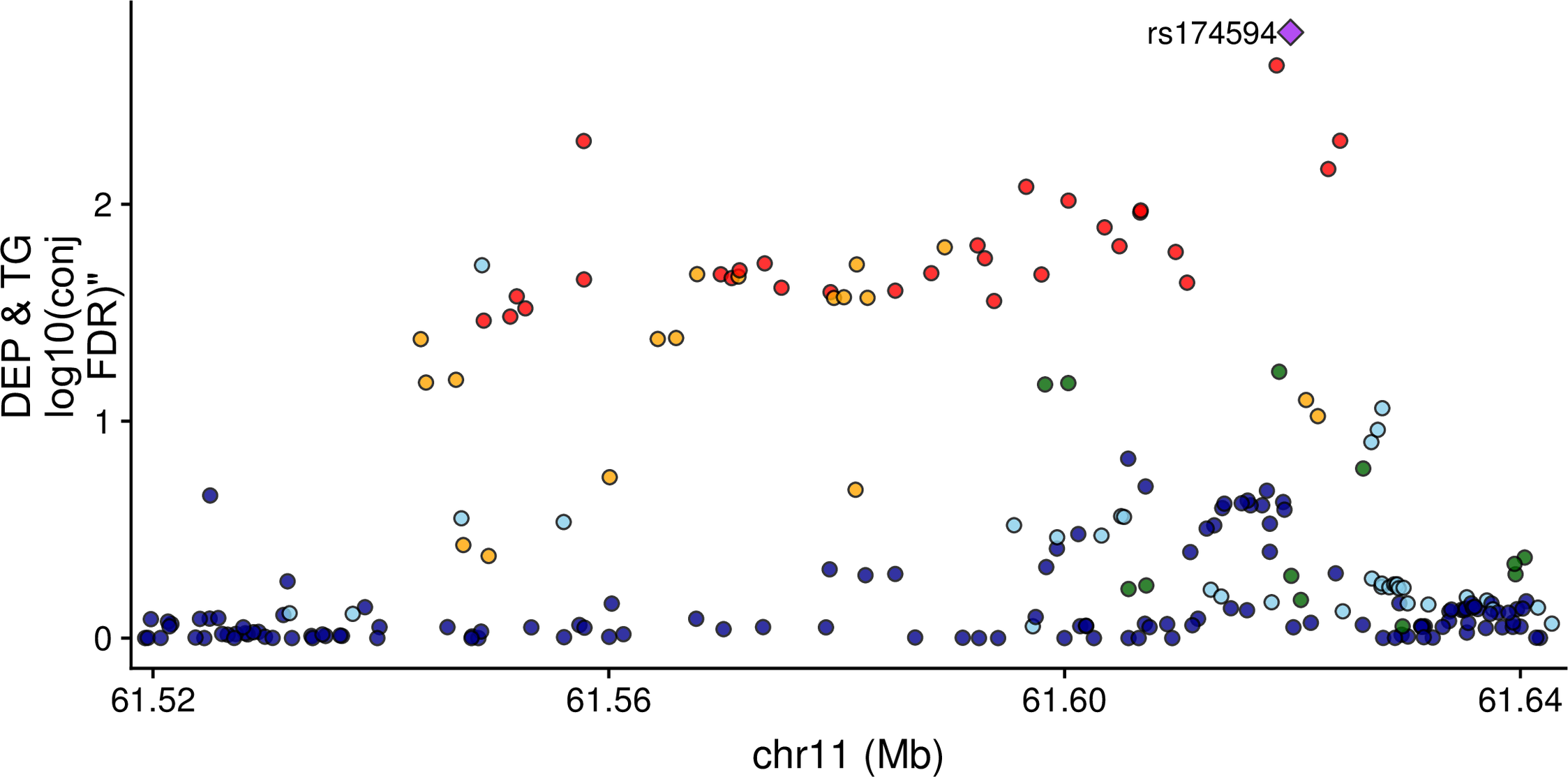

Supplement: S40 Fig — This leadSNP was in common for depression, HDL, LDL, TC and TG. (TIF) [file pgen.1010161.s040.tif]

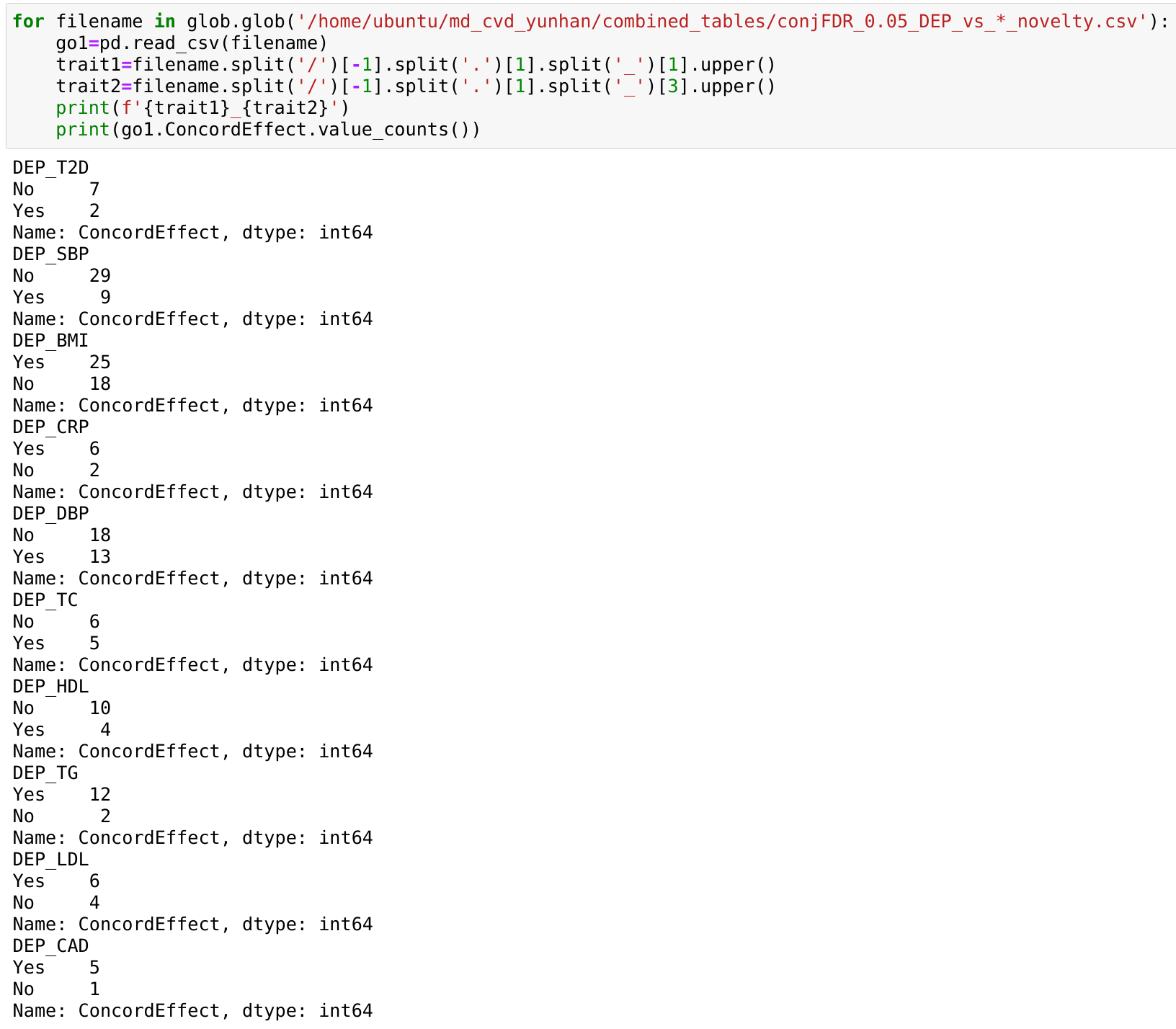

Supplement: S9 Table — (TIF) [file pgen.1010161.s049.tif]

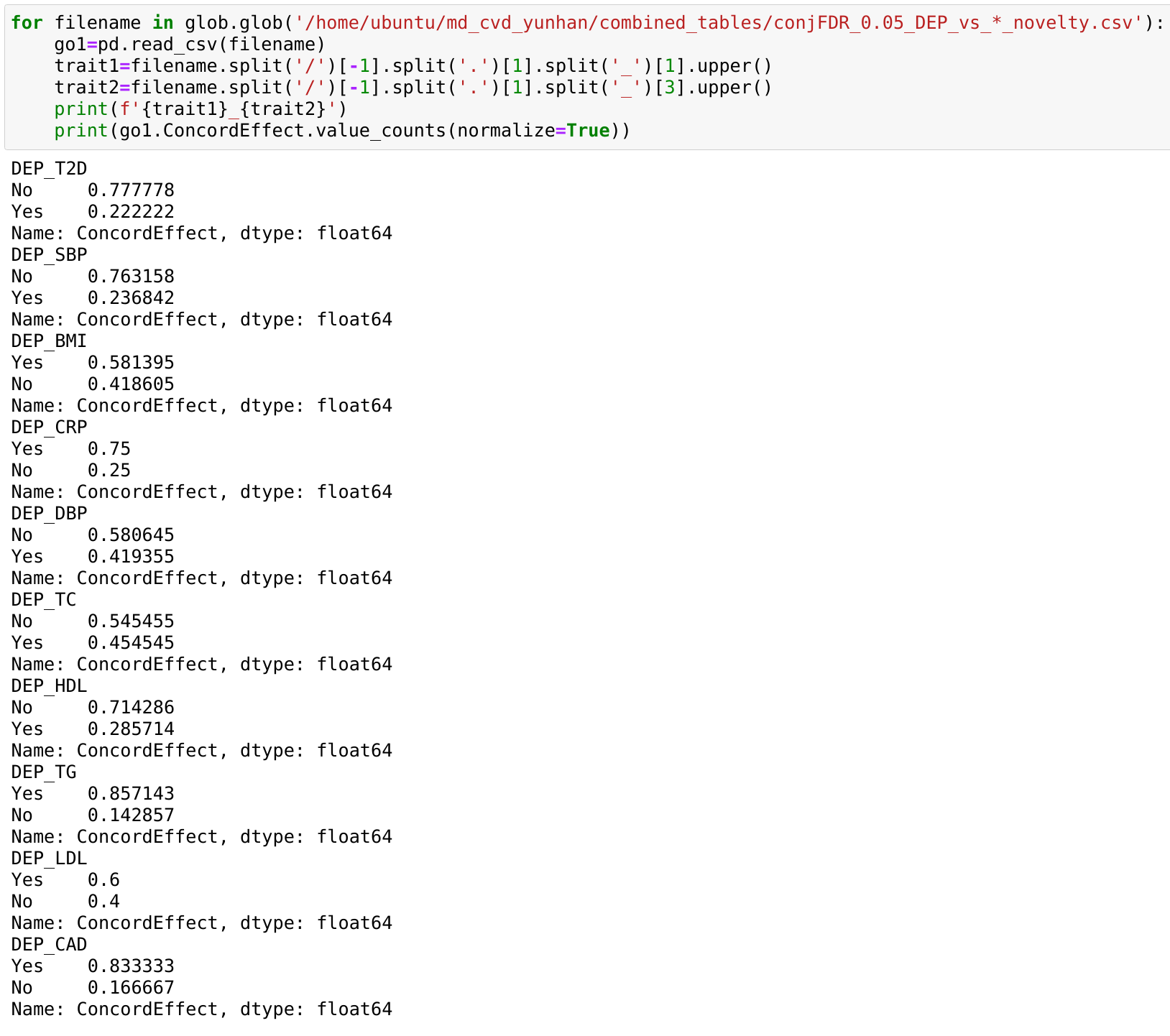

Supplement: S10 Table — (TIF) [file pgen.1010161.s050.tif]

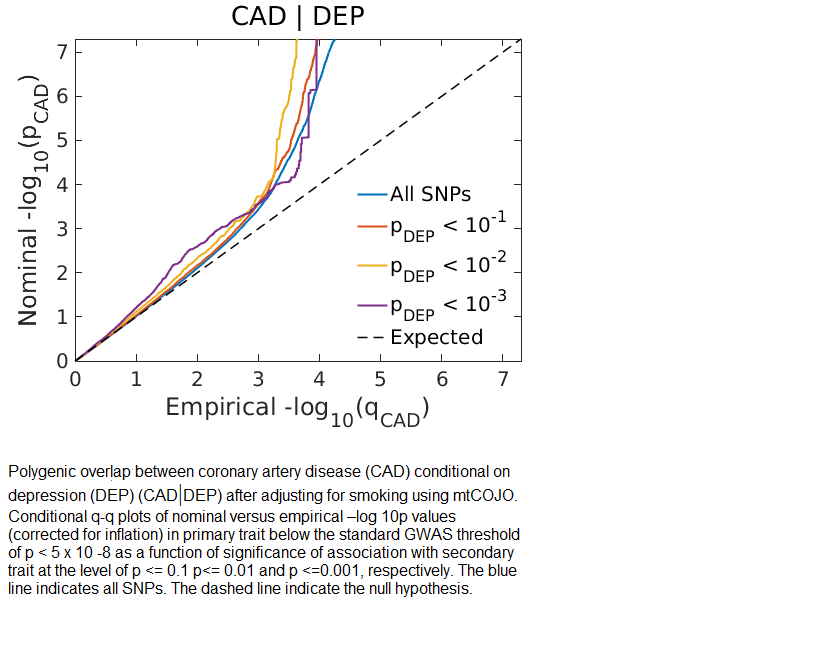

Supplement: S1 Data — (ZIP) [file pgen.1010161.s054.zip › mtCOJO/mtCOJO_CAD_vs_DEP_qq.tif]

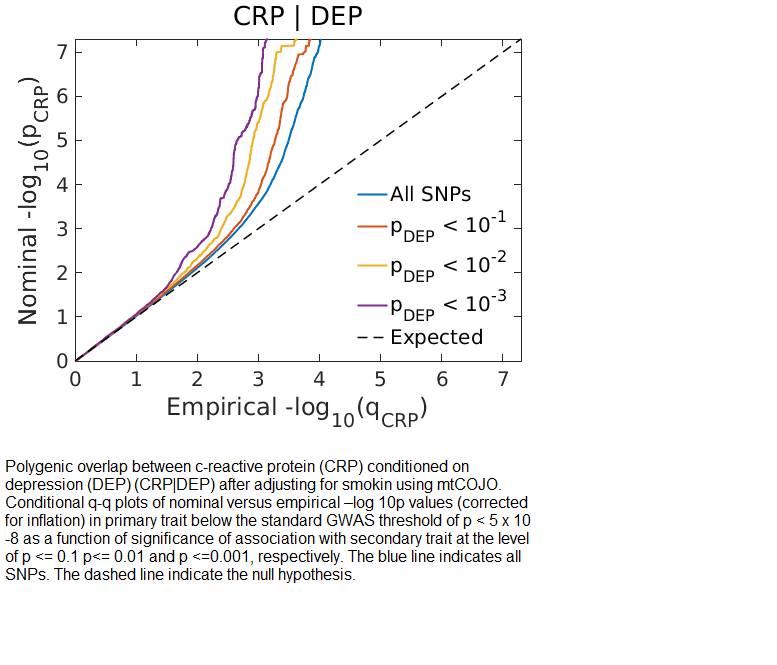

Supplement: S1 Data — (ZIP) [file pgen.1010161.s054.zip › mtCOJO/mtCOJO_CRP_vs_DEP_qq.tif]

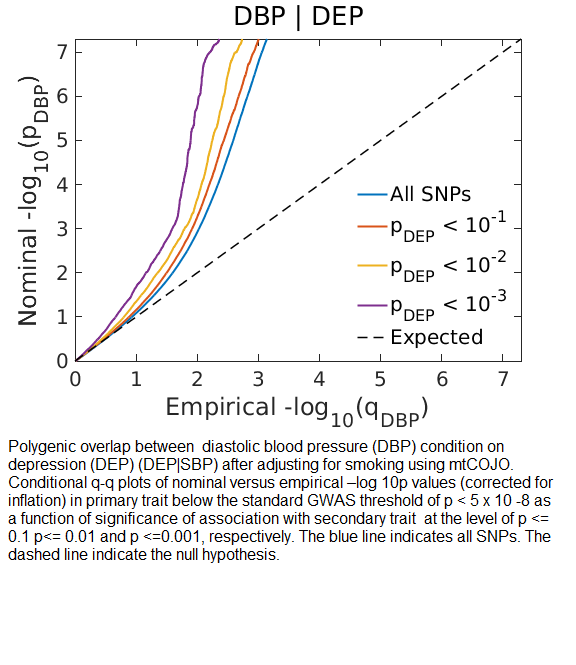

Supplement: S1 Data — (ZIP) [file pgen.1010161.s054.zip › mtCOJO/mtCOJO_DBP_vs_DEP_qq.png]

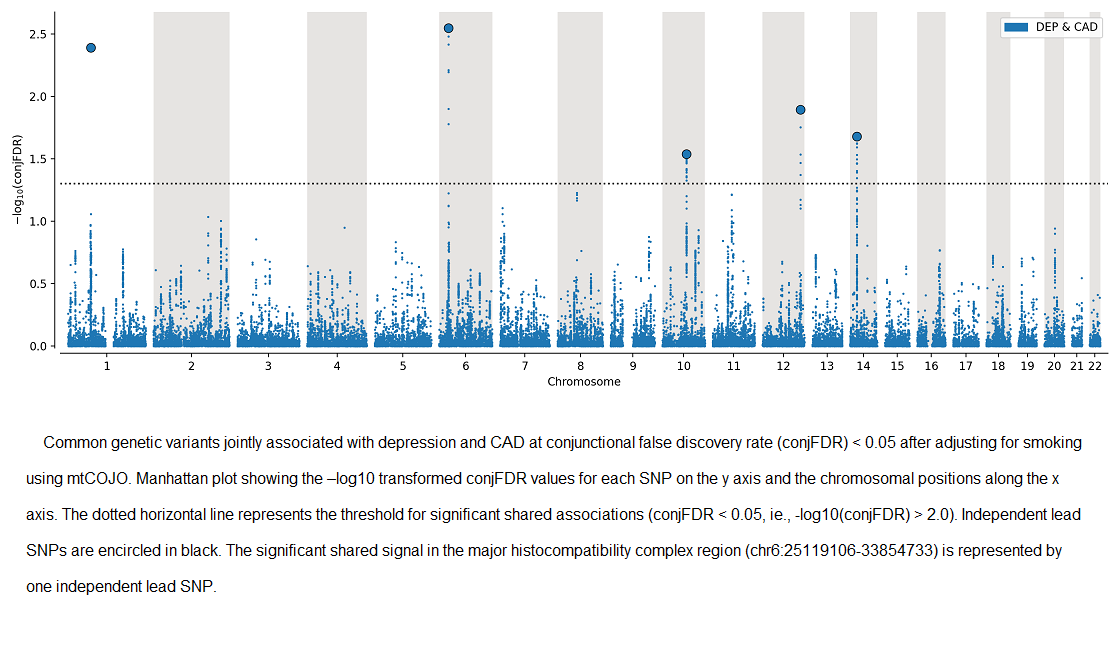

Supplement: S1 Data — (ZIP) [file pgen.1010161.s054.zip › mtCOJO/mtCOJO_DEP_CAD_conj_manhattan.tif]

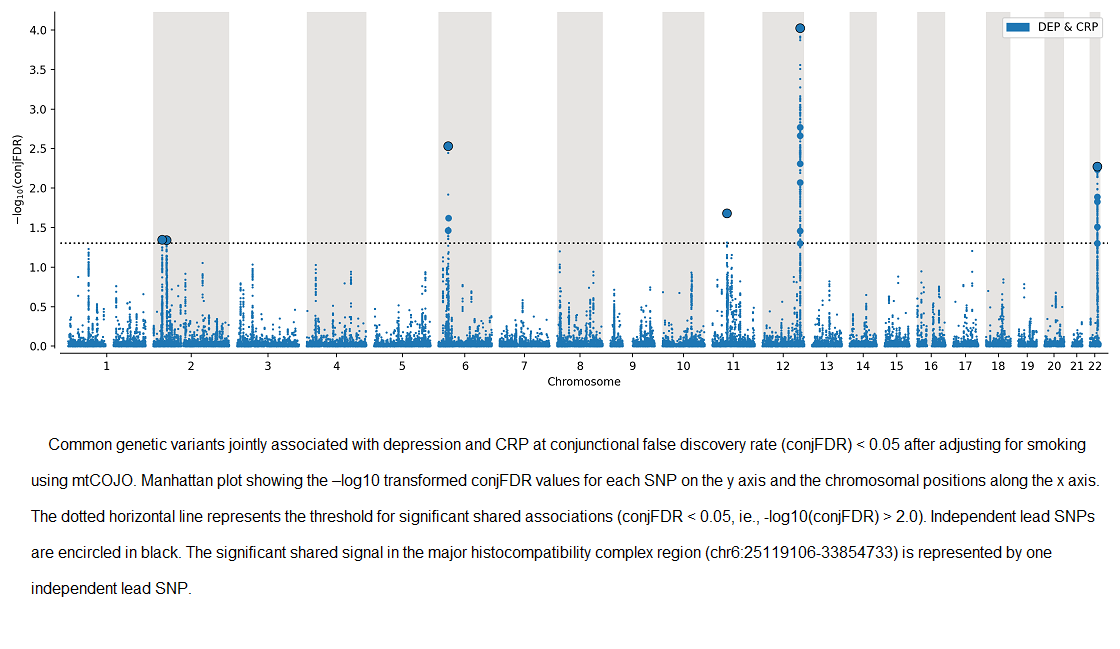

Supplement: S1 Data — (ZIP) [file pgen.1010161.s054.zip › mtCOJO/mtCOJO_DEP_CRP_conj_manhattan.tif]

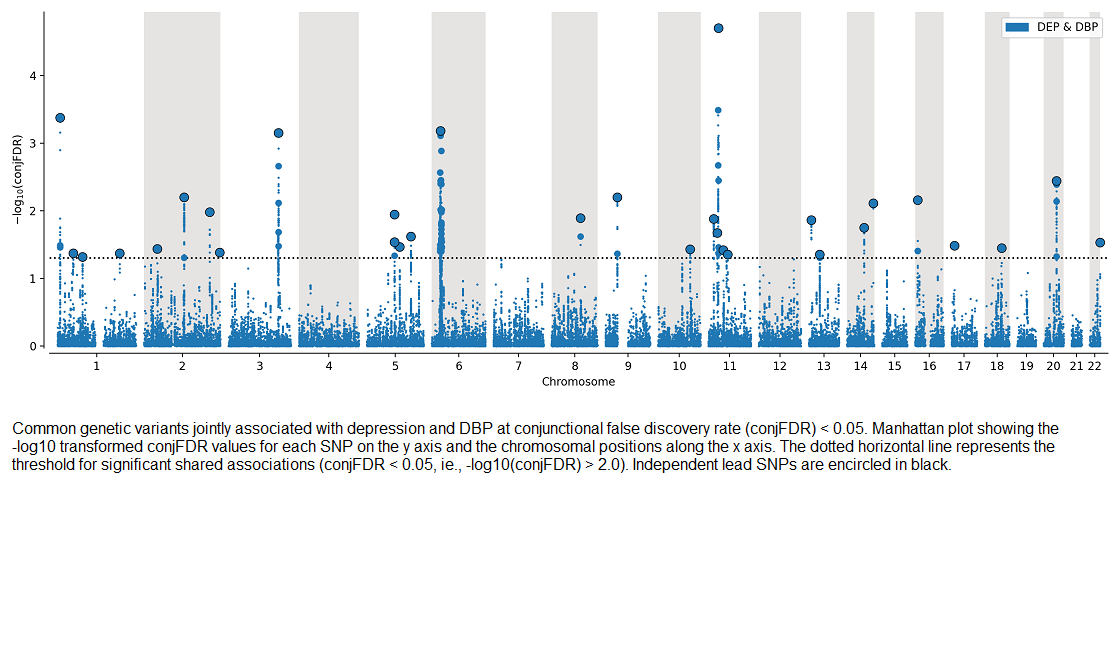

Supplement: S1 Data — (ZIP) [file pgen.1010161.s054.zip › mtCOJO/mtCOJO_DEP_DBP_conj_clump_manhattan.tif]

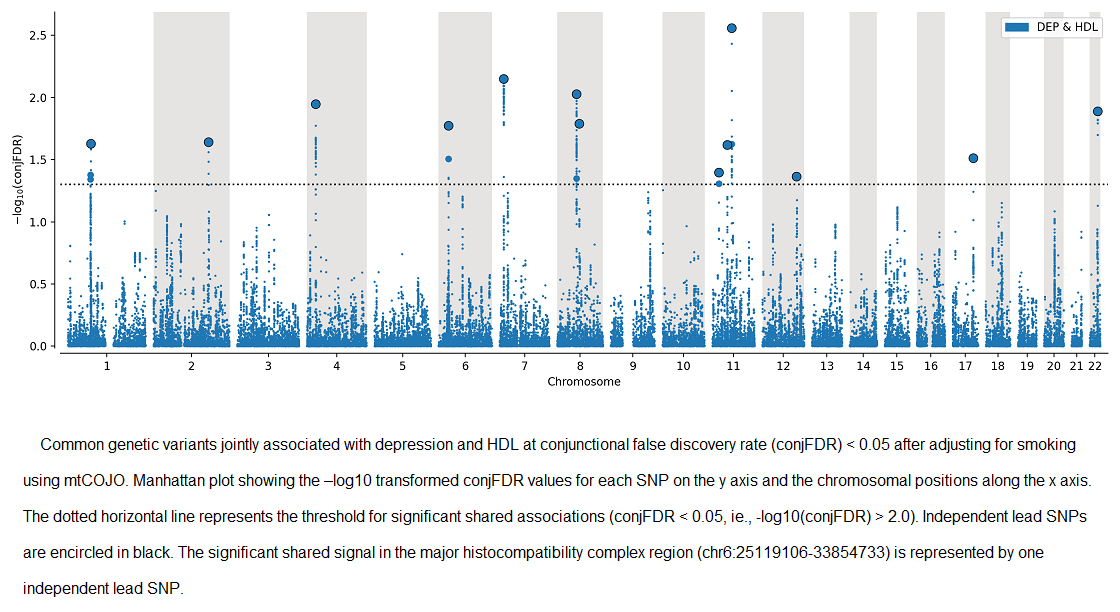

Supplement: S1 Data — (ZIP) [file pgen.1010161.s054.zip › mtCOJO/mtCOJO_DEP_HDL_conj_manhattan.tif]

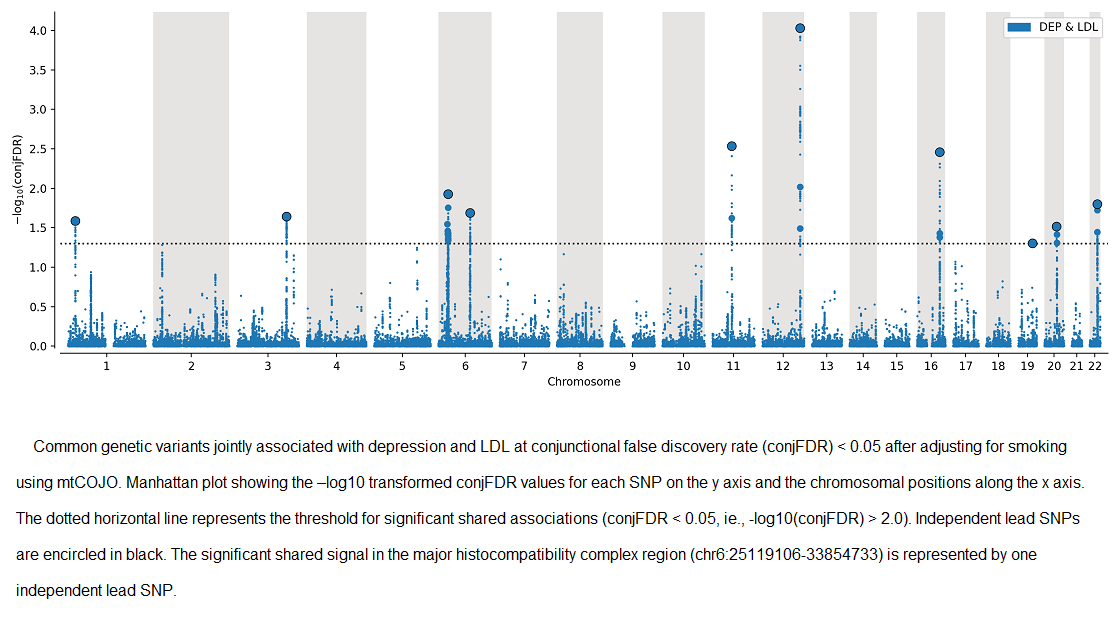

Supplement: S1 Data — (ZIP) [file pgen.1010161.s054.zip › mtCOJO/mtCOJO_DEP_LDL_conj_manhattan.tif]

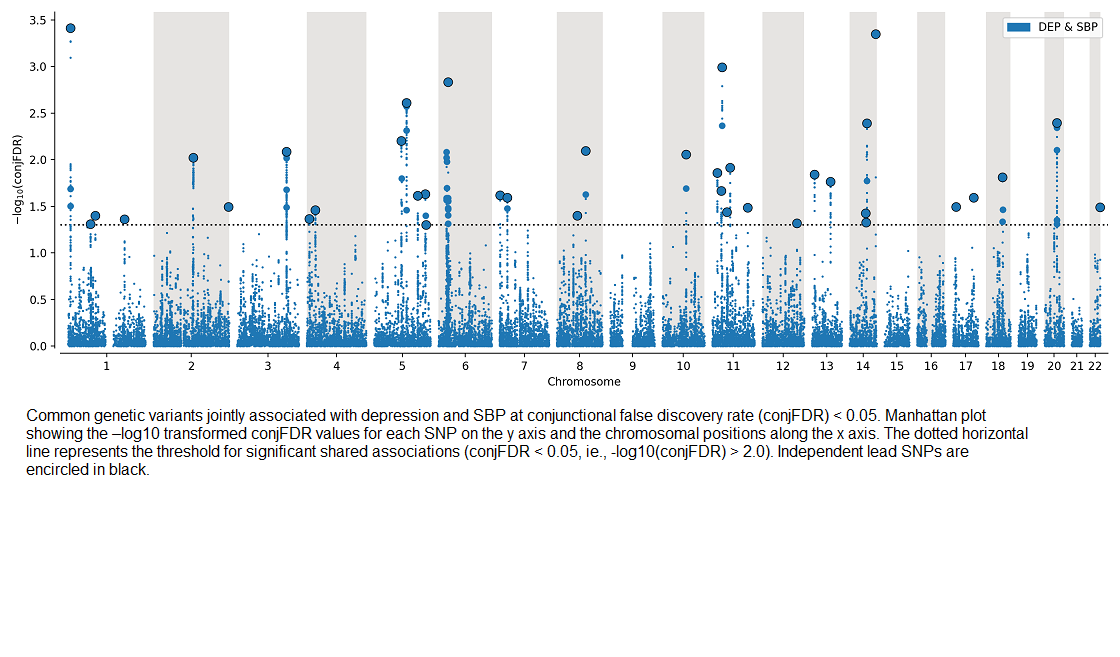

Supplement: S1 Data — (ZIP) [file pgen.1010161.s054.zip › mtCOJO/mtCOJO_DEP_SBP_conj_clump_manhattan.tif]

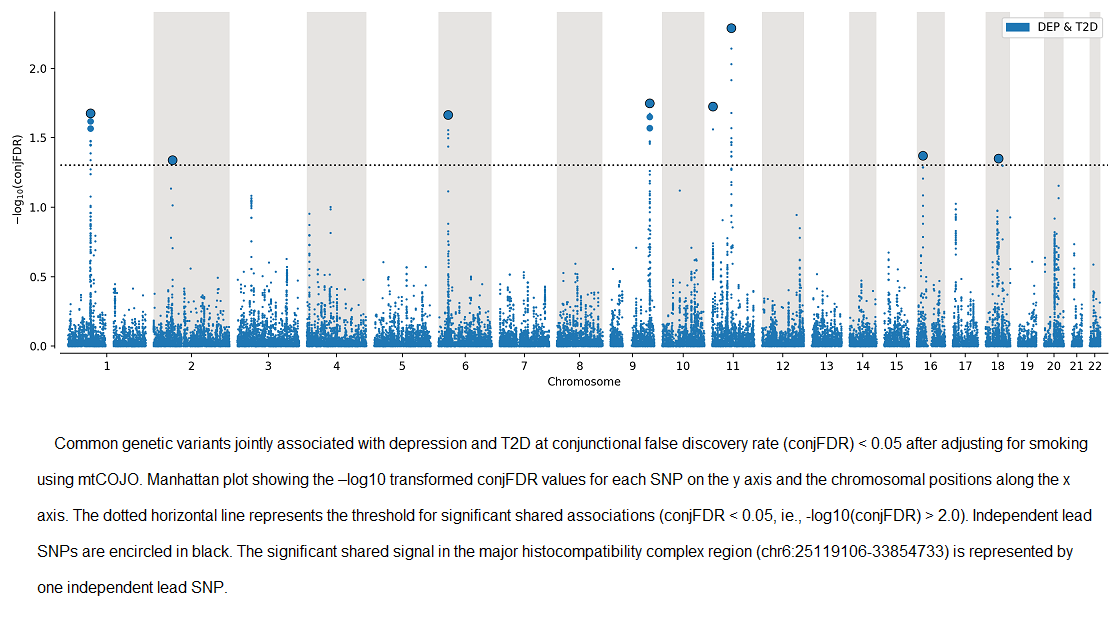

Supplement: S1 Data — (ZIP) [file pgen.1010161.s054.zip › mtCOJO/mtCOJO_dep_t2d_conj_manhattan.tif]

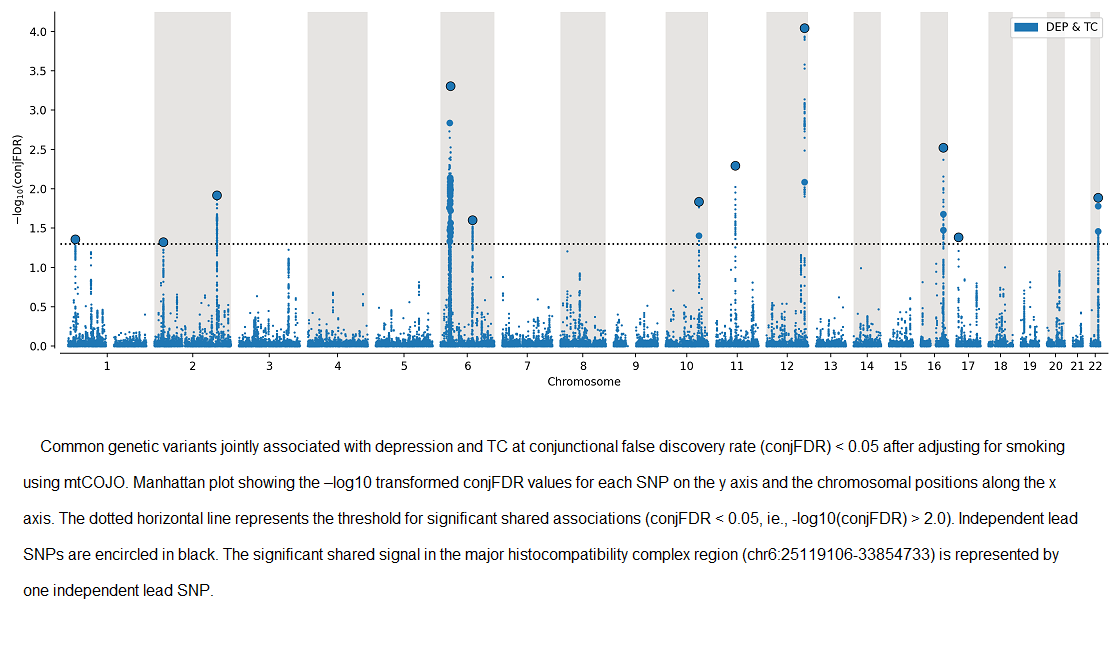

Supplement: S1 Data — (ZIP) [file pgen.1010161.s054.zip › mtCOJO/mtCOJO_dep_tc_conj_manhattan.tif]

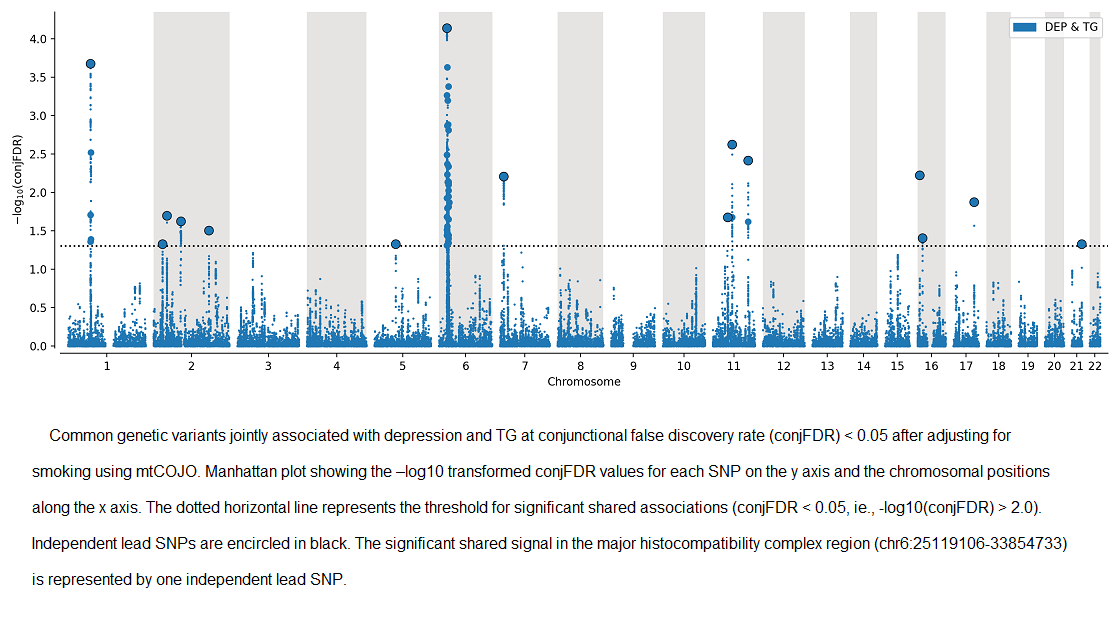

Supplement: S1 Data — (ZIP) [file pgen.1010161.s054.zip › mtCOJO/mtCOJO_dep_tg_conj_manhattan.tif]

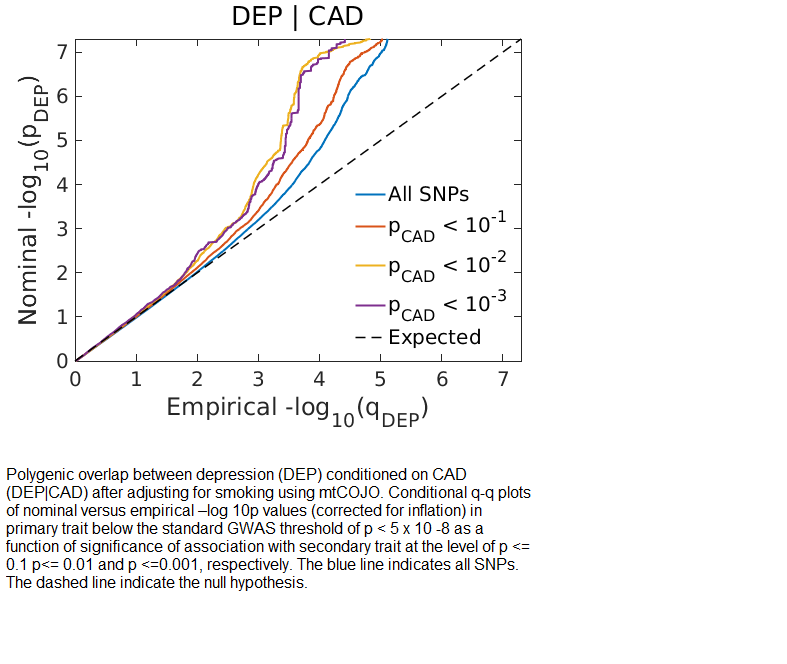

Supplement: S1 Data — (ZIP) [file pgen.1010161.s054.zip › mtCOJO/mtCOJO_DEP_vs_CAD_qq.tif]

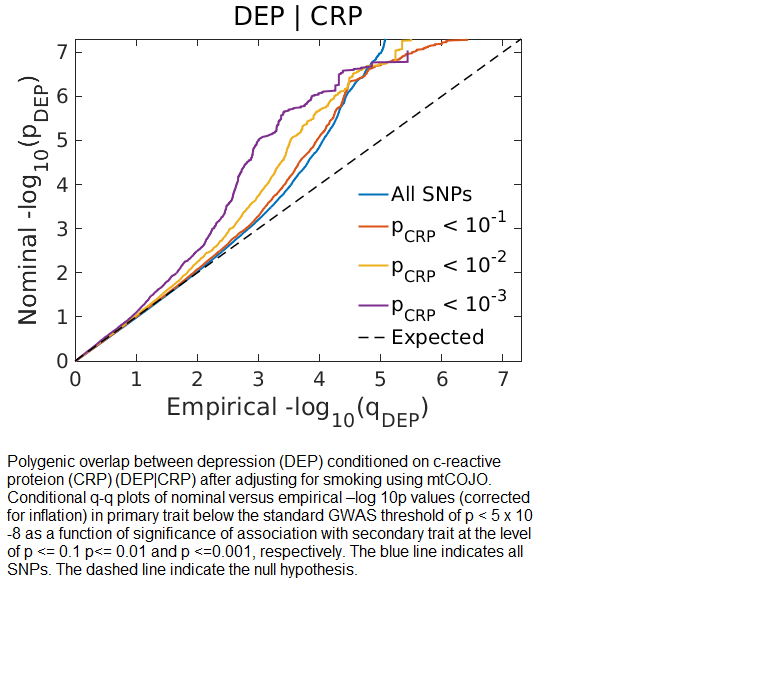

Supplement: S1 Data — (ZIP) [file pgen.1010161.s054.zip › mtCOJO/mtCOJO_DEP_vs_CRP_qq.tif]

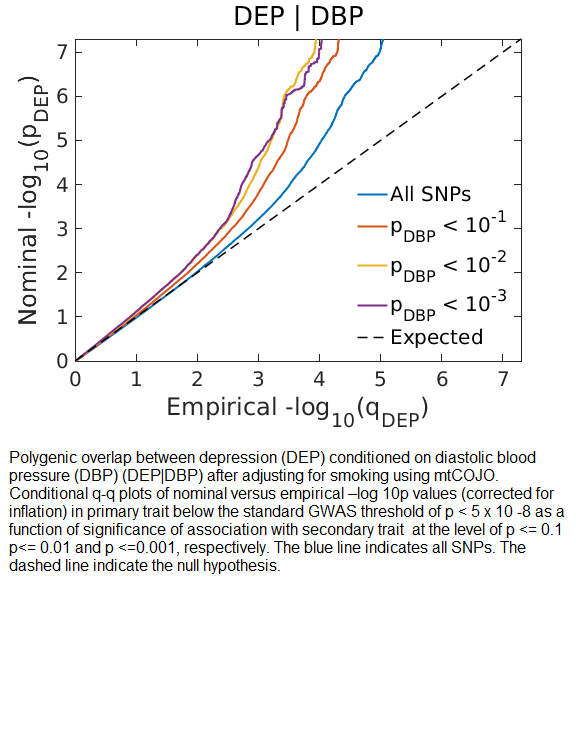

Supplement: S1 Data — (ZIP) [file pgen.1010161.s054.zip › mtCOJO/mtCOJO_DEP_vs_DBP_qq.png]

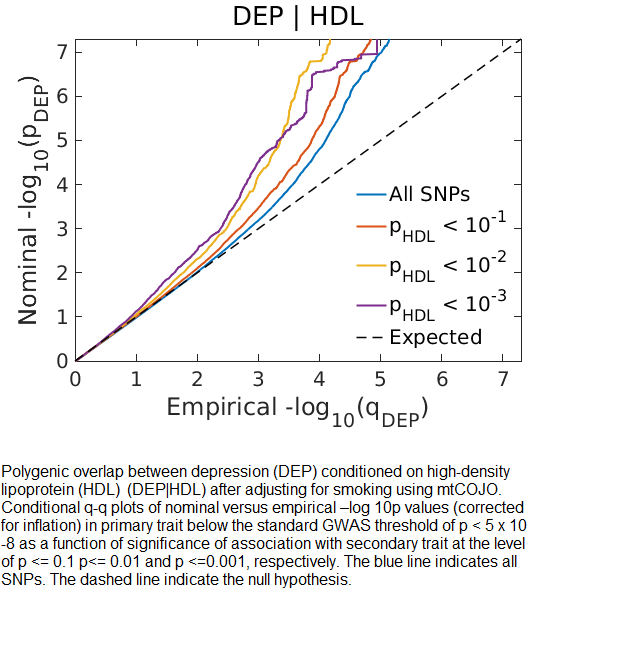

Supplement: S1 Data — (ZIP) [file pgen.1010161.s054.zip › mtCOJO/mtCOJO_DEP_vs_HDL_qq.tif]

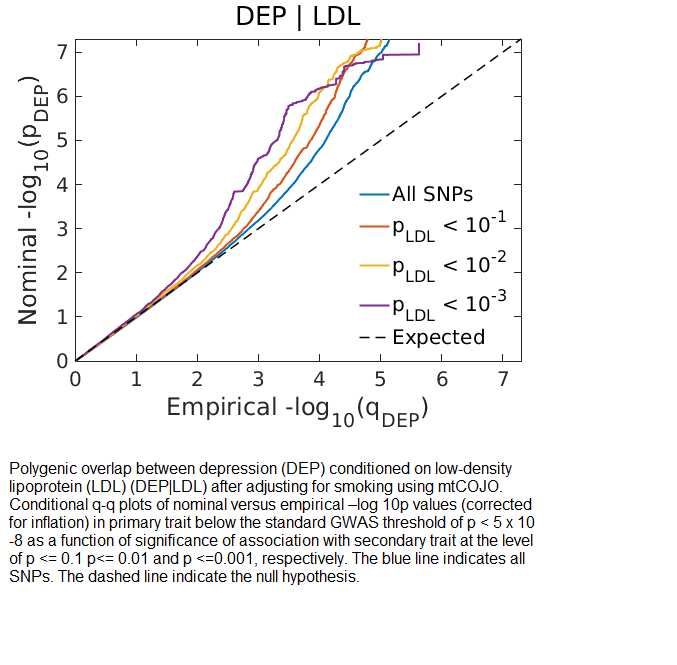

Supplement: S1 Data — (ZIP) [file pgen.1010161.s054.zip › mtCOJO/mtCOJO_DEP_vs_LDL_qq.tif]

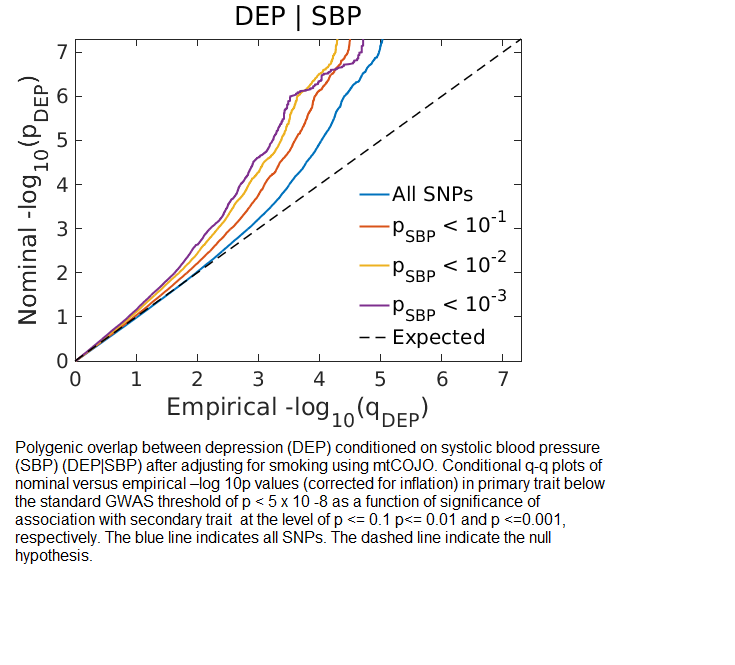

Supplement: S1 Data — (ZIP) [file pgen.1010161.s054.zip › mtCOJO/mtCOJO_DEP_vs_SBP_qq.png]

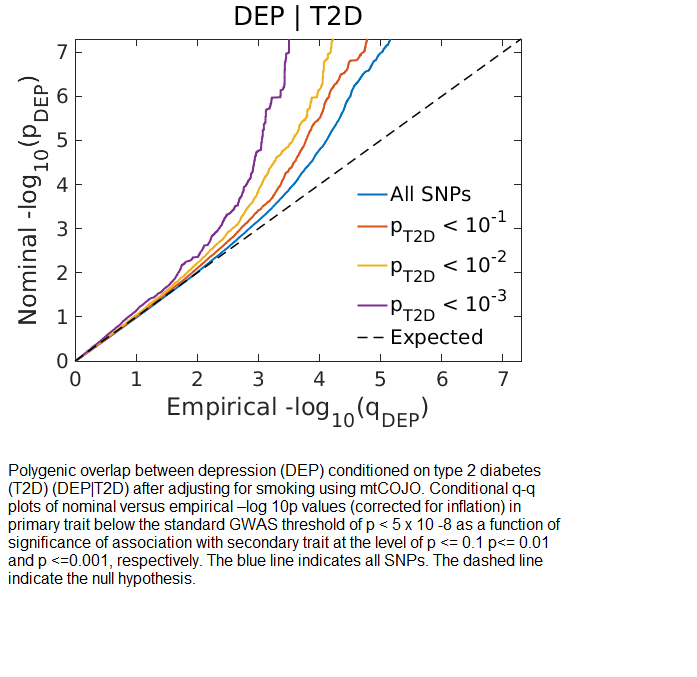

Supplement: S1 Data — (ZIP) [file pgen.1010161.s054.zip › mtCOJO/mtCOJO_DEP_vs_T2D_qq.tif]

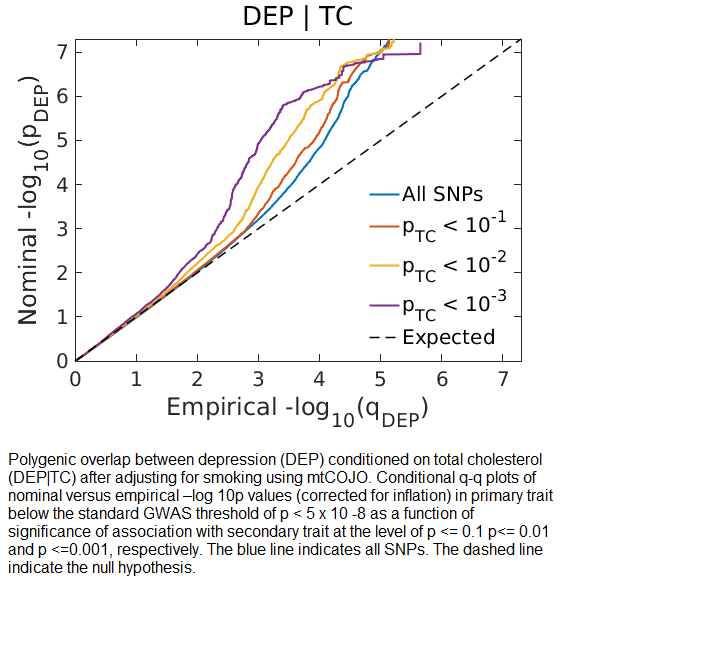

Supplement: S1 Data — (ZIP) [file pgen.1010161.s054.zip › mtCOJO/mtCOJO_DEP_vs_TC_qq.tif]

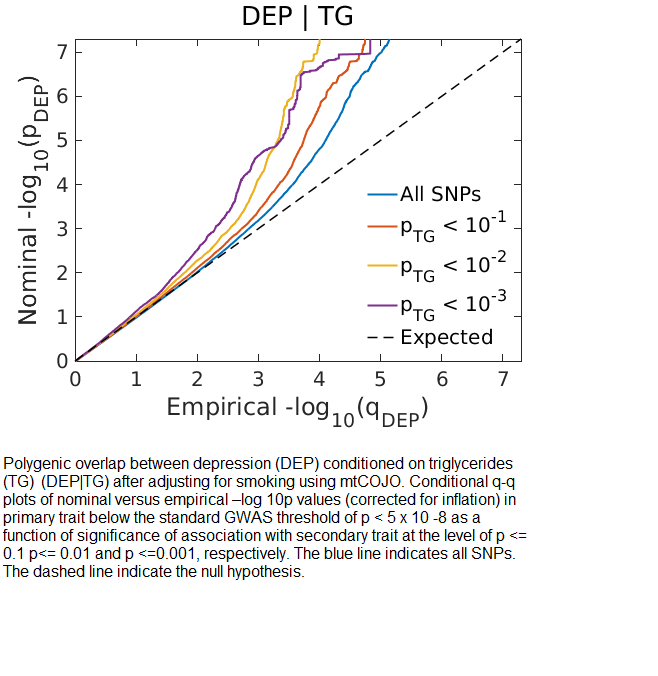

Supplement: S1 Data — (ZIP) [file pgen.1010161.s054.zip › mtCOJO/mtCOJO_DEP_vs_TG_qq.tif]

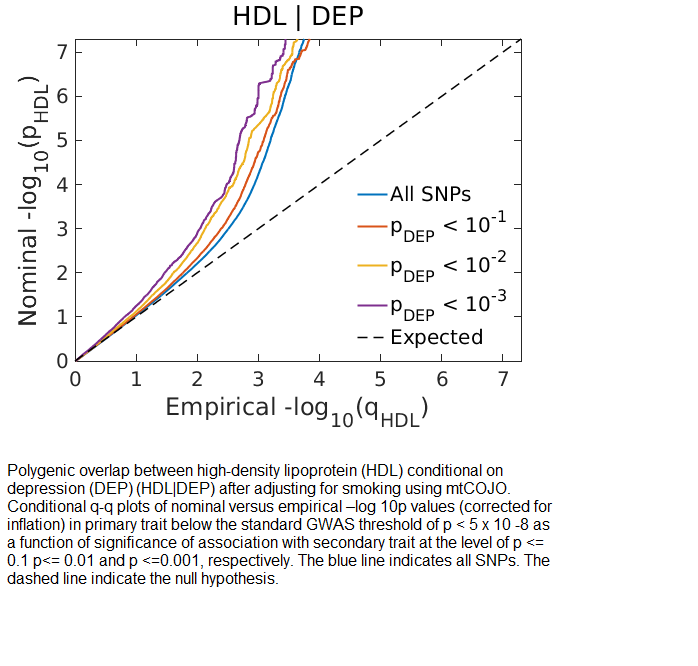

Supplement: S1 Data — (ZIP) [file pgen.1010161.s054.zip › mtCOJO/mtCOJO_HDL_vs_DEP_qq.tif]

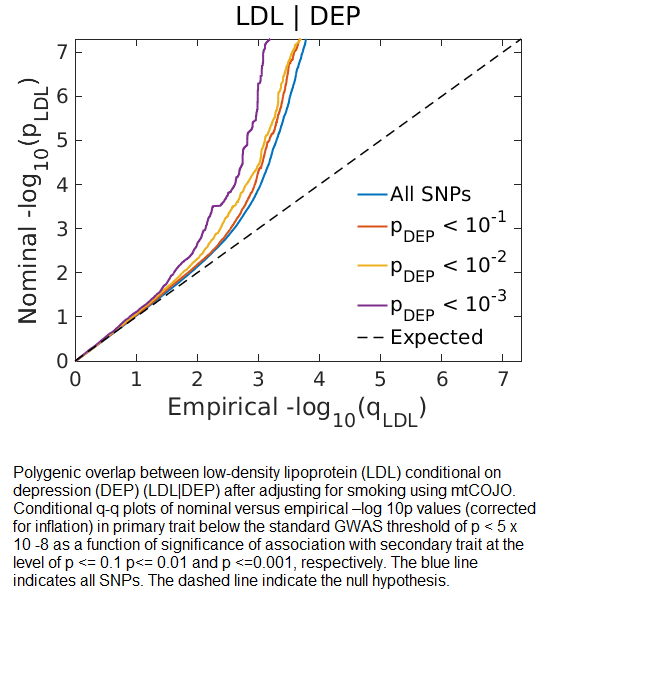

Supplement: S1 Data — (ZIP) [file pgen.1010161.s054.zip › mtCOJO/mtCOJO_LDL_vs_DEP_qq.tif]

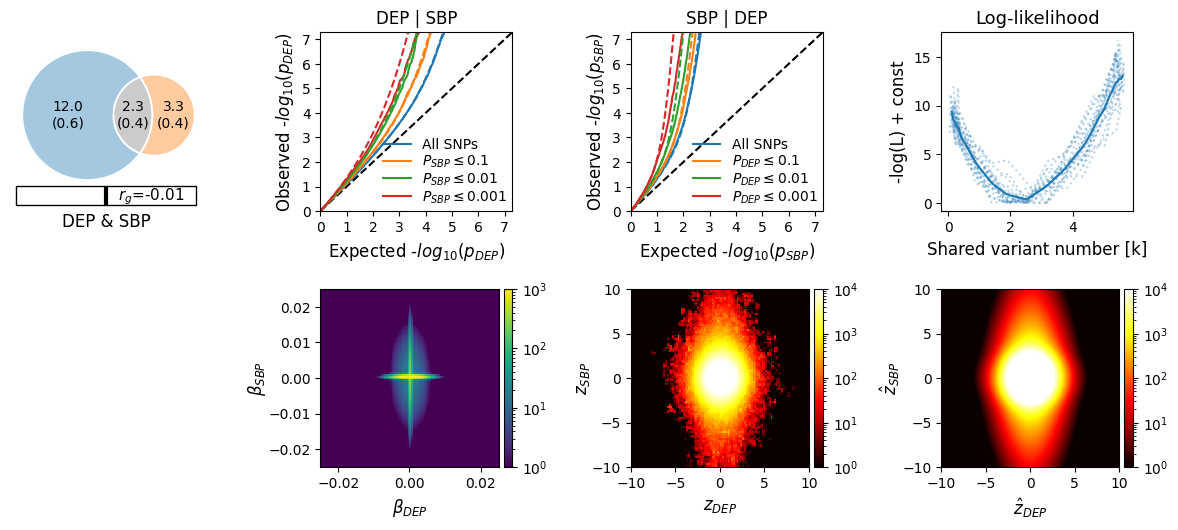

Supplement: S1 Data — (ZIP) [file pgen.1010161.s054.zip › mtCOJO/mtCOJO_MiXeR_ DEP_vs_SBP.combined.png]

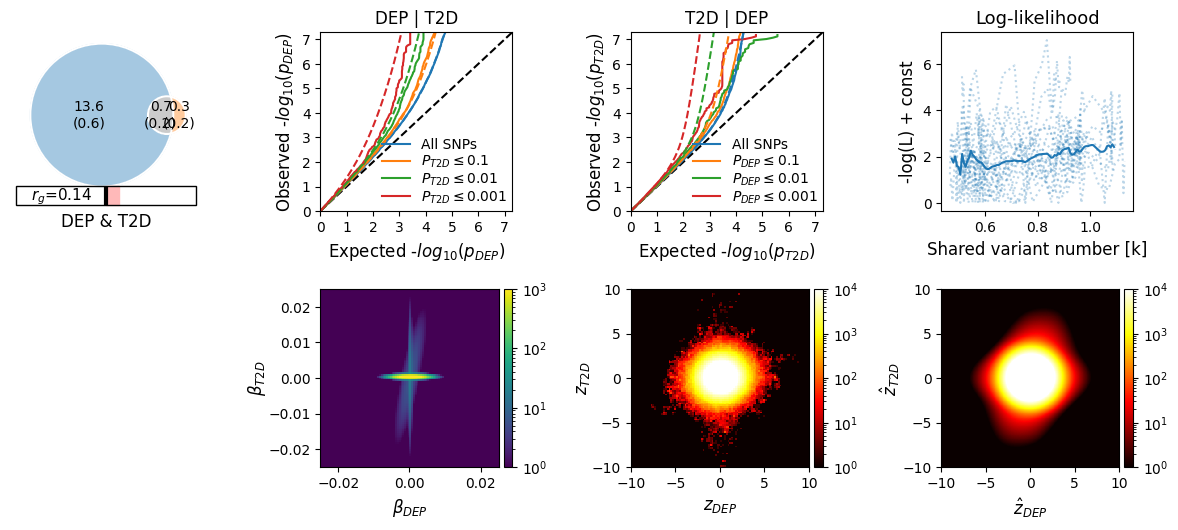

Supplement: S1 Data — (ZIP) [file pgen.1010161.s054.zip › mtCOJO/mtCOJO_MiXeR_ DEP_vs_T2D.combined.png]

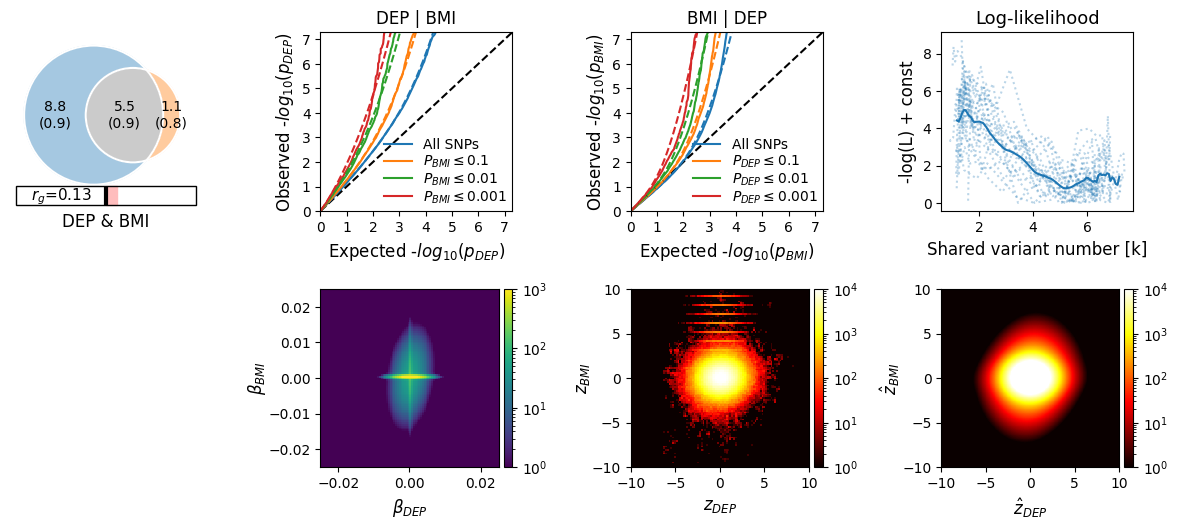

Supplement: S1 Data — (ZIP) [file pgen.1010161.s054.zip › mtCOJO/mtCOJO_MiXeR_DEP_vs_BMI.combined.png]

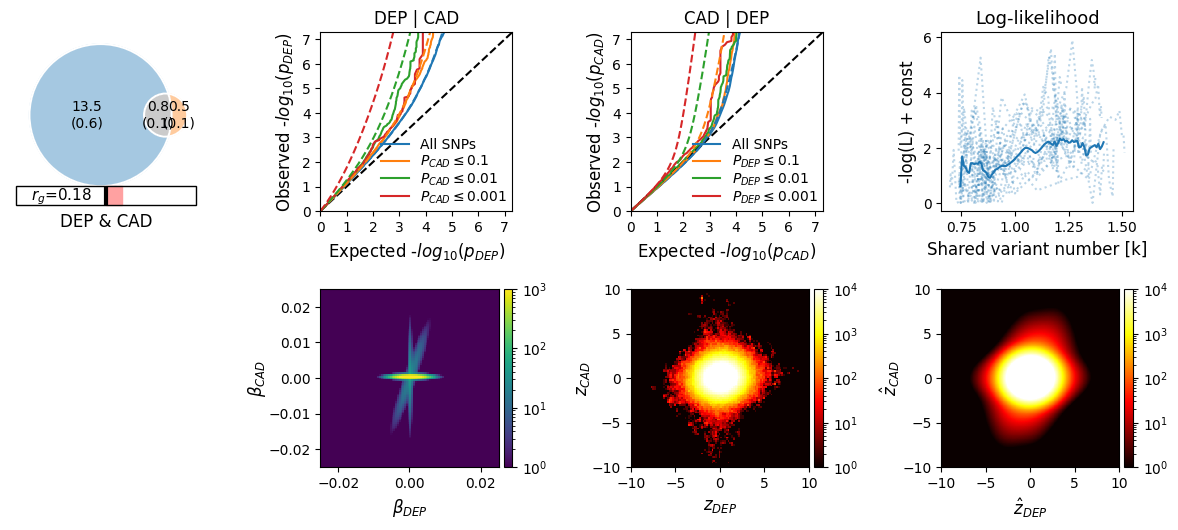

Supplement: S1 Data — (ZIP) [file pgen.1010161.s054.zip › mtCOJO/mtCOJO_MiXeR_DEP_vs_CAD.combined.png]

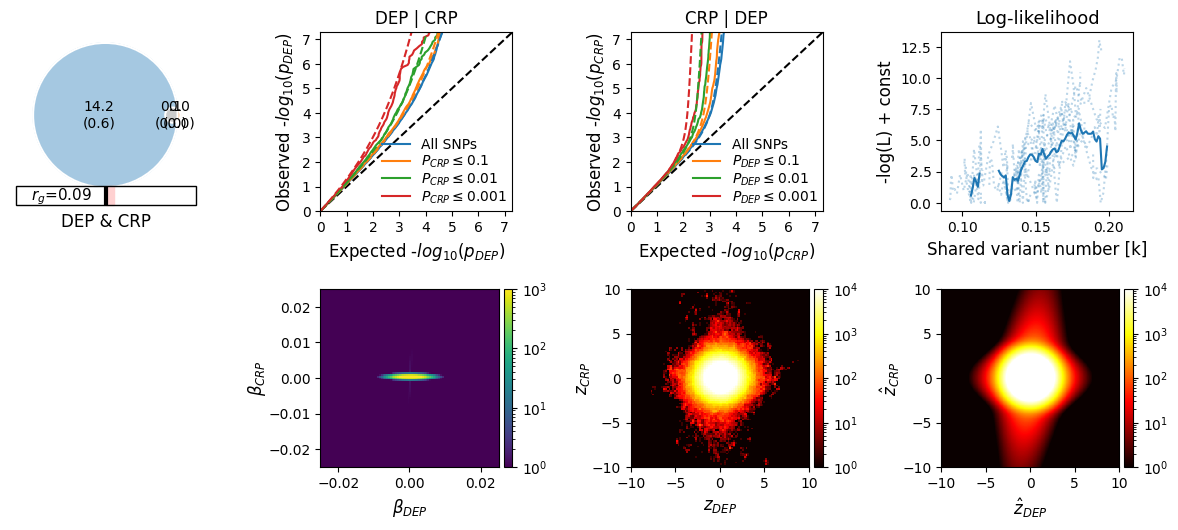

Supplement: S1 Data — (ZIP) [file pgen.1010161.s054.zip › mtCOJO/mtCOJO_MiXeR_DEP_vs_CRP.combined.png]

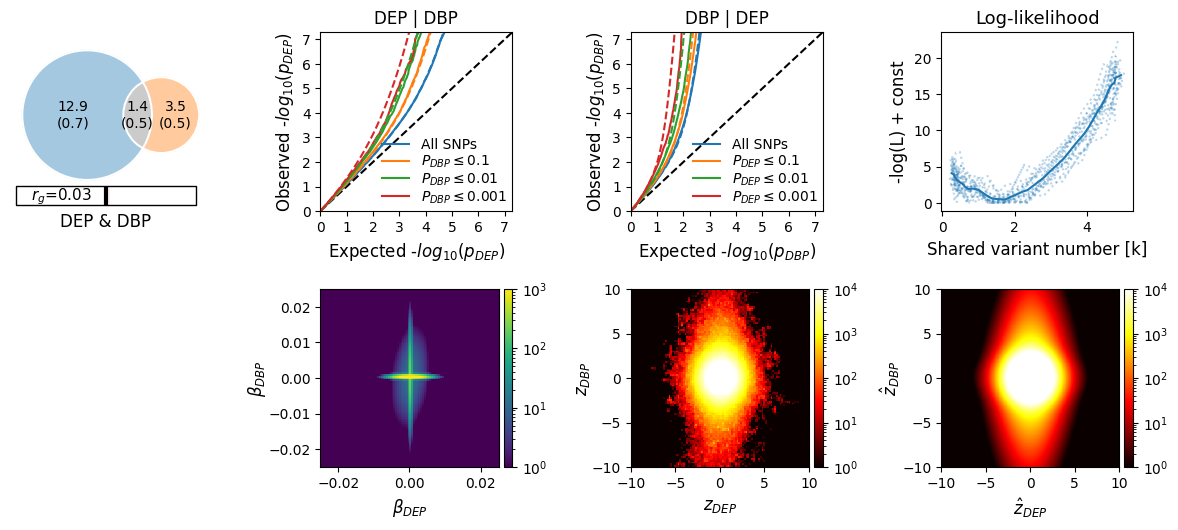

Supplement: S1 Data — (ZIP) [file pgen.1010161.s054.zip › mtCOJO/mtCOJO_MiXeR_DEP_vs_DBP.combined.png]

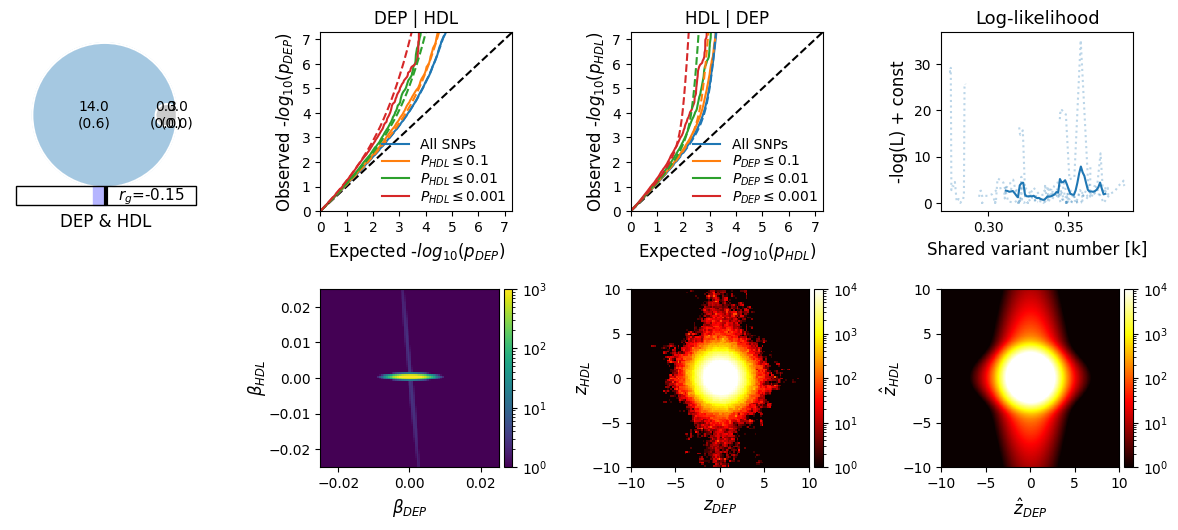

Supplement: S1 Data — (ZIP) [file pgen.1010161.s054.zip › mtCOJO/mtCOJO_MiXeR_DEP_vs_HDL.combined.png]

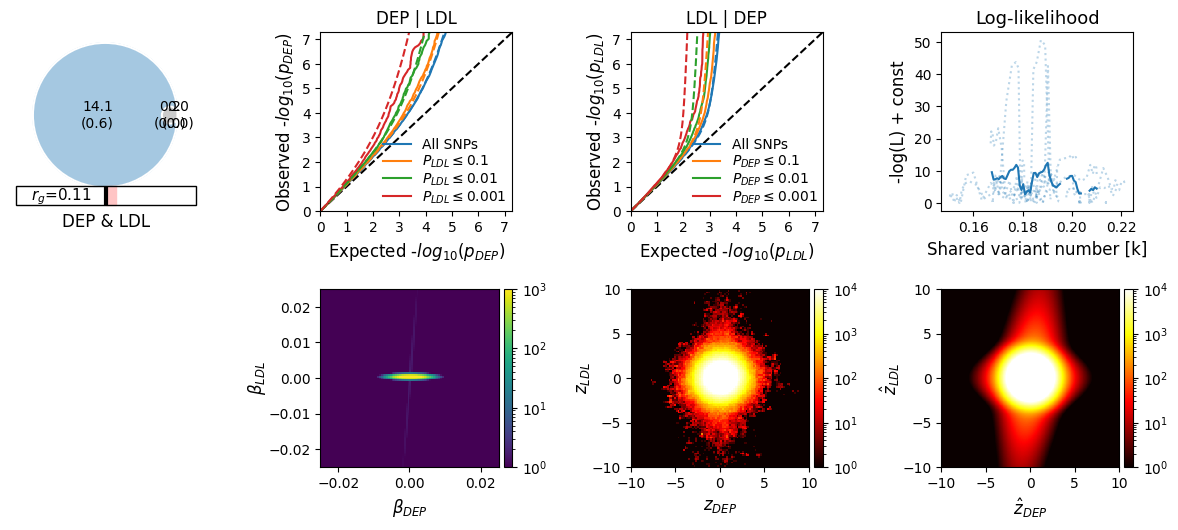

Supplement: S1 Data — (ZIP) [file pgen.1010161.s054.zip › mtCOJO/mtCOJO_MiXeR_DEP_vs_LDL.combined.png]

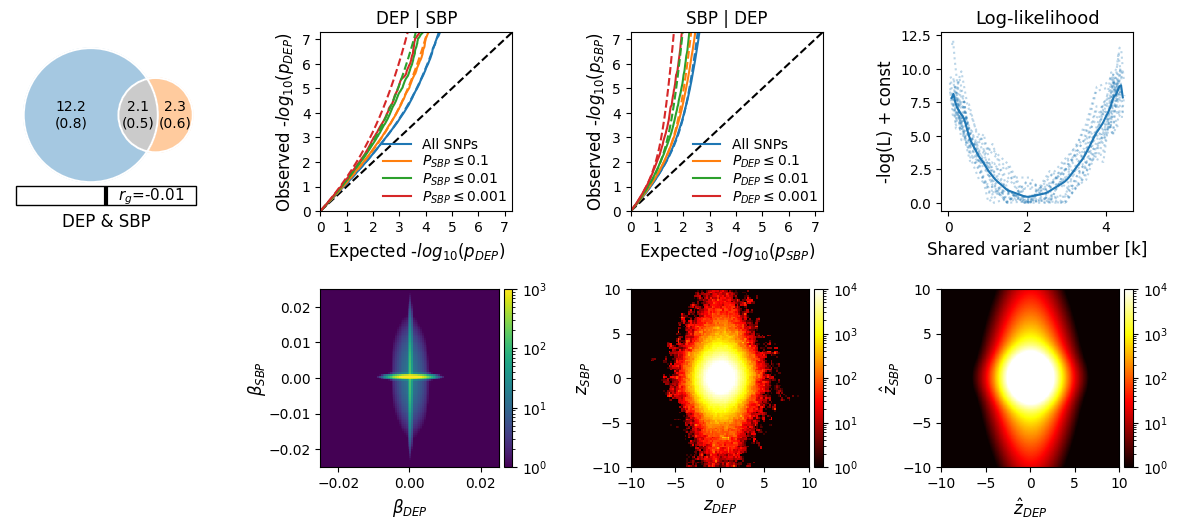

Supplement: S1 Data — (ZIP) [file pgen.1010161.s054.zip › mtCOJO/mtCOJO_MiXeR_DEP_vs_SBP.combined.png]

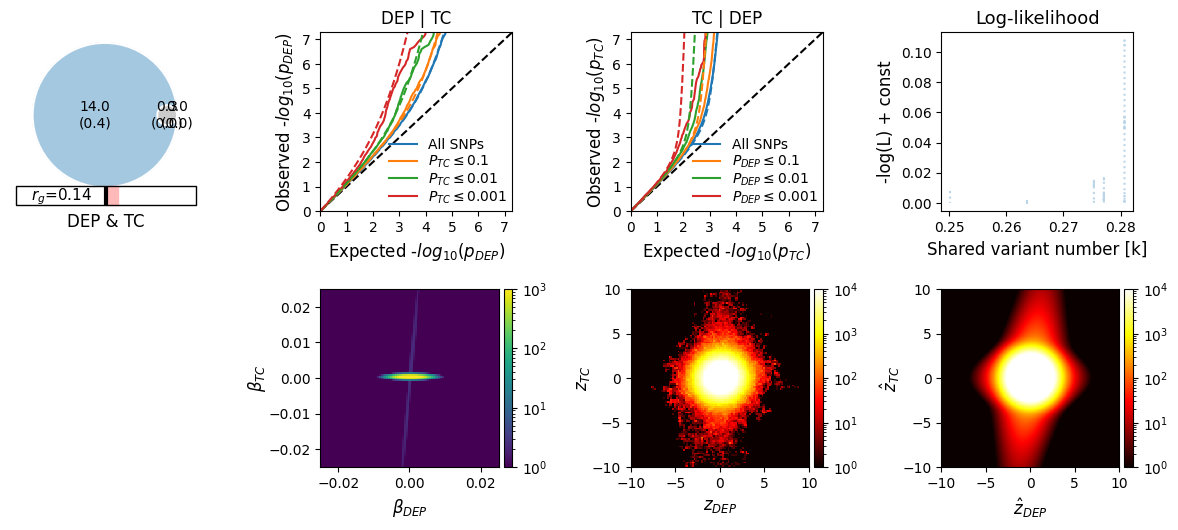

Supplement: S1 Data — (ZIP) [file pgen.1010161.s054.zip › mtCOJO/mtCOJO_MiXeR_DEP_vs_TC.combined.png]

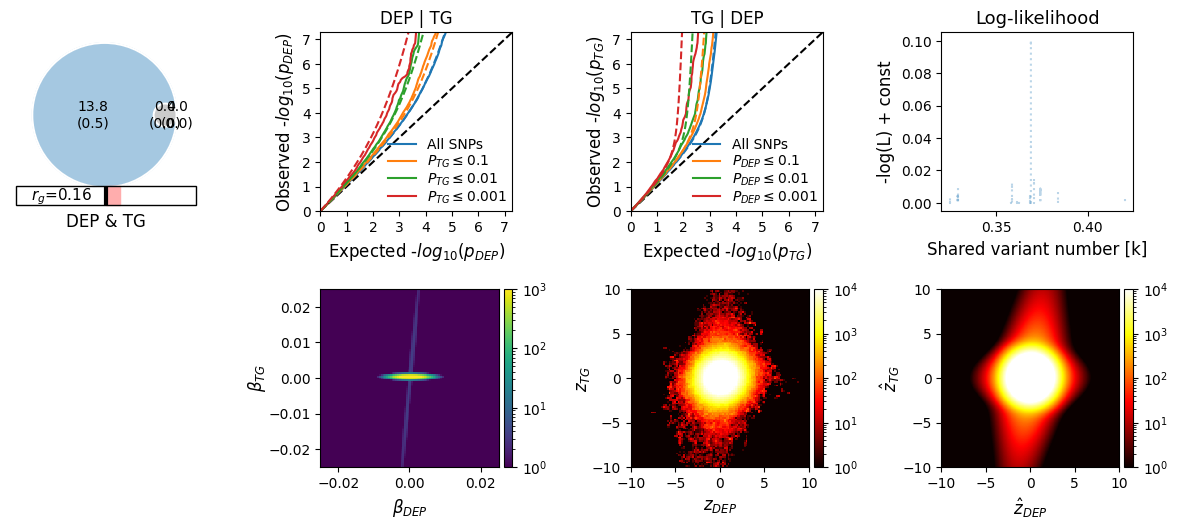

Supplement: S1 Data — (ZIP) [file pgen.1010161.s054.zip › mtCOJO/mtCOJO_MiXeR_DEP_vs_TG.combined.png]

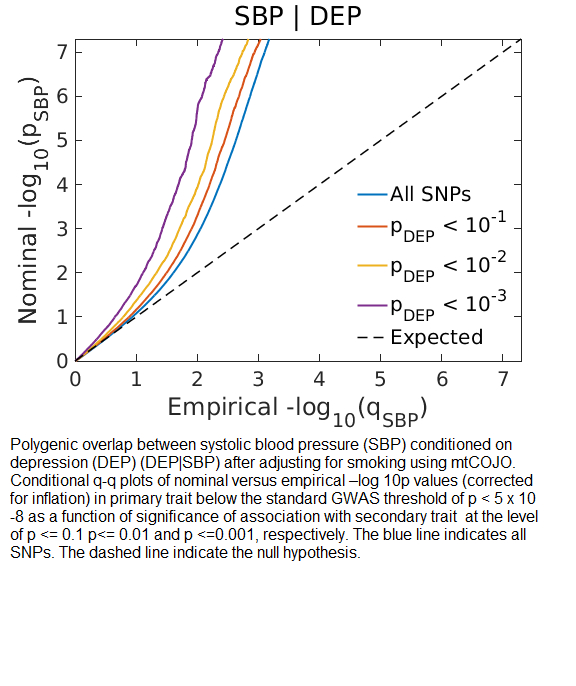

Supplement: S1 Data — (ZIP) [file pgen.1010161.s054.zip › mtCOJO/mtCOJO_SBP_vs_DEP_qq.png]

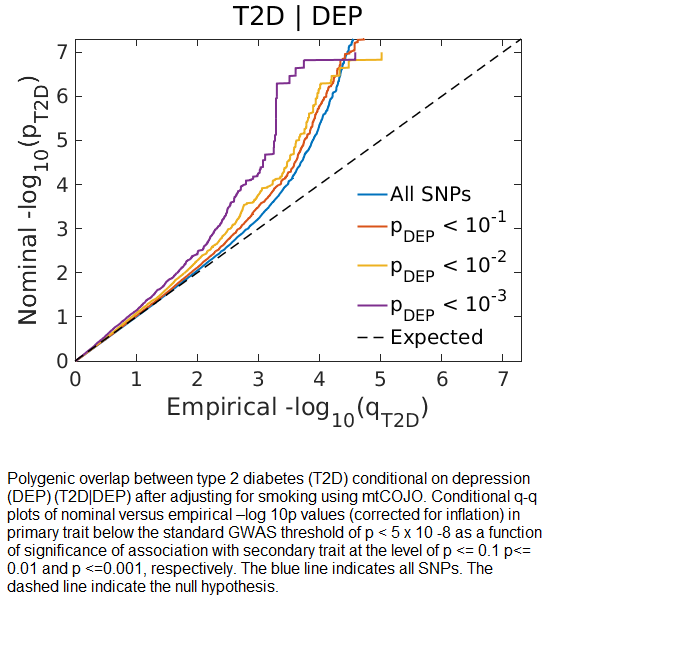

Supplement: S1 Data — (ZIP) [file pgen.1010161.s054.zip › mtCOJO/mtCOJO_T2D_vs_DEP_qq.tif]

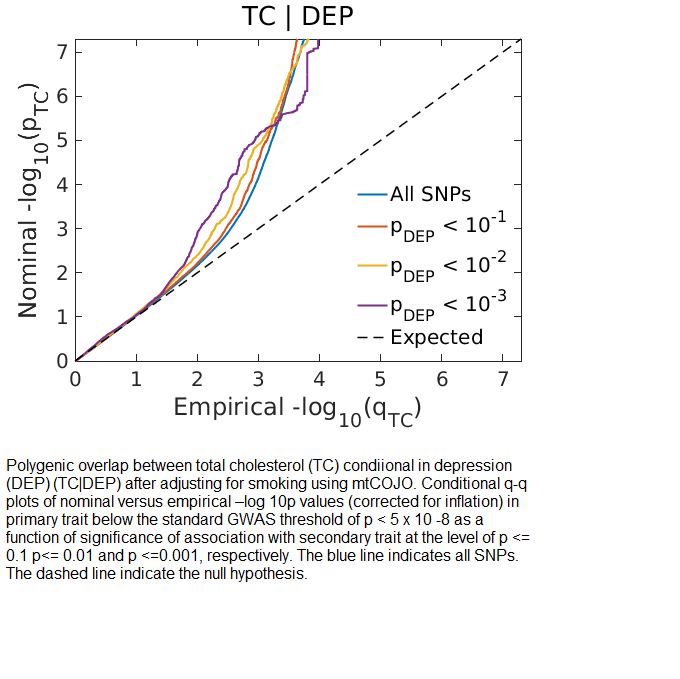

Supplement: S1 Data — (ZIP) [file pgen.1010161.s054.zip › mtCOJO/mtCOJO_TC_vs_DEP_qq.tif]

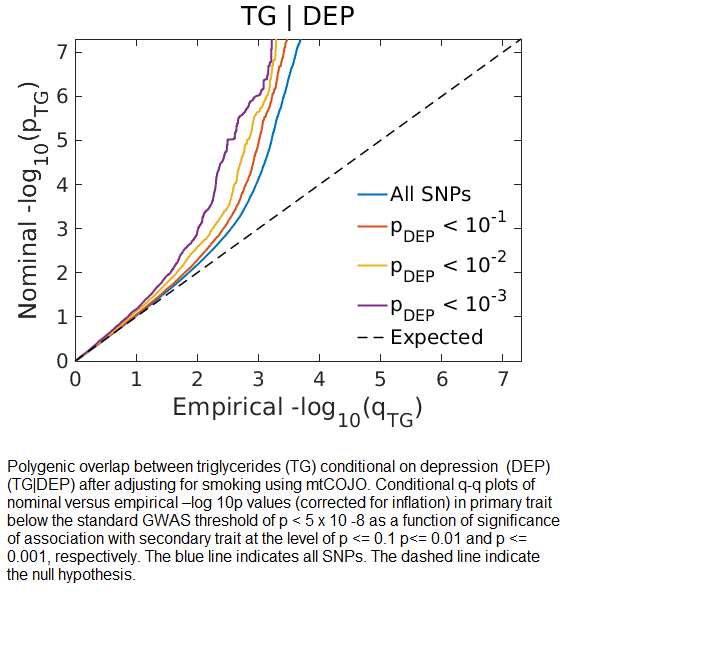

Supplement: S1 Data — (ZIP) [file pgen.1010161.s054.zip › mtCOJO/mtCOJO_TG_vs_DEP_qq.tif]

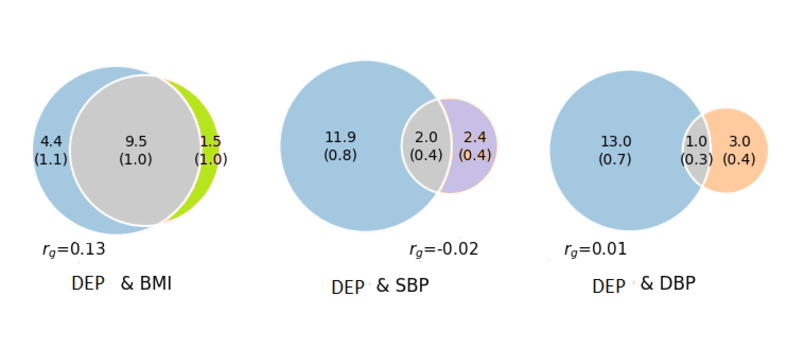

Supplement: S1 Data — (ZIP) [file pgen.1010161.s054.zip › Fig1.tif]
