# Supplementary material for: Shared genetic loci between depression and cardiometabolic traits
Source: PLoS Genet. 2022 May 13;18(5):e1010161. doi: 10.1371/journal.pgen.1010161 (PMC9170110; doi:10.1371/journal.pgen.1010161)
Supplement: S1 Table — (DOCX) [file pgen.1010161.s041.docx]

| **Sample** | **Country** | **Cases characteristics** | **Controls characteristics** | **Inclution criteria Cases** | **Exclution criteria Cases** | **Exclution criteria Controls** |
| --- | --- | --- | --- | --- | --- | --- |
| **deCODE 1** | Iceland | Inpatient records, random sample using SSQ & HADS; CIDI | Multiple studies, matched by sex, birth year, county of birth | DSM-III, ICD-9 or ICD-10 MDD (recurrent or moderate-severe single episode) | BIP, SCZ | MDD (self-report); BIP, SCZ; HADS≥7; AD use |
| **GenScotland 2,3** | UK | Random sample of patients in general practice; SCID | Drawn from same cohort. SCID | DSM-IV MDD | BIP | MDD |
| **GERA 4** | USA | Kaiser Permanente Northern California (1995-2013); EMR | Kaiser Permanente Northern California (1995-2013); EMR | ICD-9 MDD on ≥2 separate medical visits | BIP, SCZ | ICD-9 codes MDD, insomnia, stress, irritable bowel syndrome, other psych dx (BIP, NAP, SUD, PTSD, eating disorders, anxiety disorders) |
| **iPSYCH 5** | Denmark | All births 1981–2005, diagnoses from national psychiatric treatment register | Randomly selected from same birth cohort | ICD-10 MDD | BIP or manic episode | MDD, manic episode or BIP |
| **23andMe 6** | USA | Commercial direct-to-consumer genomics company, consent for research; self-report | Commercial direct-to-consumer genomics company, consent for research; self-report | MDD (diagnosed with clinical depression or depression diagnosed by a doctor) | BIP, SCZ, autism, ID, multiple personality, Parkinson's disease | As for cases plus self-reported depression, anxiety disorder, fibromyalgia, or AD use |

Abbreviations: AD=antidepressant, BIP=bipolar disorder, CIDI=Composite International Diagnostic Interview, CIDI-SF=CIDI-short form, DIGS=Diagnostic Interview for Genetic Studies, Dx=diagnosis, EMR=electronic medical record, FHx=family history, Hx=history, ID=intellectual disability, NAP=non-affective psychosis, SADS=Schedule for Affective Disorders and Schizophrenia, SCID=Structured Clinical Interview for DSM-IV, SUD=substance use disorder, Sx=symptoms, and Tx=treatment.

|  | ***Citation*** | |  |  | |
| --- | --- | --- | --- | --- | --- |
| 1Major Depressive Disorder Working Group of the PGC. A mega-analysis of genome-wide association studies for major depressive disorder. *Molecular Psychiatry* **18**, 497-511 (2013). | | | | |  |
| 2Smith BH *et al.* Cohort Profile: Generation Scotland: Scottish Family Health Study (GS:SFHS). The study, its participants and their potential for genetic research on health and illness. *Int J Epidemiol* **42**, 689-700 (2013). | | | | |  |
| 3Fernandez-Pujals AM *et al.* Epidemiology and Heritability of Major Depressive Disorder, Stratified by Age of Onset, Sex, and Illness Course in Generation Scotland: Scottish Family Health Study (GS:SFHS). *PLoS One* **10**, e0142197 (2015). | | | | |  |
| 4Banda Y *et al.* Characterizing Race/Ethnicity and Genetic Ancestry for 100,000 Subjects in the Genetic Epidemiology Research on Adult Health and Aging (GERA) Cohort. *Genetics* **200**, 1285-95 (2015). | | | | |  |
| 5 Pedersen, C.B. *et al.* The iPSYCH2012 case-cohort sample: new directions for unravelling genetic and environmental architectures of severe mental disorders. *Mol Psychiatry* **23**, 6-14 (2018) | | | | |  |
| 6Hyde CL *et al.* Identification of 15 genetic loci associated with risk of major depression in individuals of European descent. *Nat Genet* **48**, 1031-6 (2016). | | | | |  |
|  |  | | | Discovery sample only | |
|  | |  |  |  |  |
|  | |  |  |  |  |
|  | |  |  |  |  |
