# Supplementary material for: Shared genetic loci between depression and cardiometabolic traits
Source: PLoS Genet. 2022 May 13;18(5):e1010161. doi: 10.1371/journal.pgen.1010161 (PMC9170110; doi:10.1371/journal.pgen.1010161)
Supplement: S1 Text — (DOCX) [file pgen.1010161.s053.docx]

**S1 Text**

**Materials and Methods**

All PGC29 samples were of European-ancestry and structured methodological reviewed by MDD assessment experts. Cases had to meet international consensus criteria (DSM-IV, ICD-9, or ICD-10) for a lifetime diagnosis of MDD established using structured diagnostic instruments from assessments by trained interviewers, clinician-administered checklists, or medical record review. All cases in all samples met standard criteria for MDD and most were ascertained from clinical sources (19/29 samples). Most samples controls were randomly selected from the general population and screened for the absence of lifetime MDD (22/29 samples). For more details, see the original GWAS [1].

The five additional independent, European-ancestry cohorts used different methods for assessing MDD or MD. Most of these studies applied typical inclusion and exclusion criteria for both cases and controls, and included cases of self-reported depression. We excluded the UK Biobank Cohort to avoid overlap between samples.

The 23andMe, Inc. sample was based on self-reported information from more than 1,000,000 individuals (90 % participating in research), through a direct-to-consumer online genetic-testing service since 2006 [2].

Summary statistics for a combined meta-analysis of PGC29 with four of the five expanded samples (deCODE, Generation Scotland, GERA, iPSYCH) are available on the PGC web site. Results for 10,000 SNPs for the PGC29 and the five additional cohorts are also available on the PGC web page.

**Suppl. Table 1 Description of the five additional cohorts**

| **Sample** | **Country** | **Cases characteristics** | **Controls characteristics** | **Inclution criteria Cases** | **Exclution criteria Cases** | **Exclution criteria Controls** |
| --- | --- | --- | --- | --- | --- | --- |
| **deCODE [3]** | Iceland | Inpatient records, random sample using SSQ & HADS; CIDI | Multiple studies, matched by sex, birth year, county of birth | DSM-III, ICD-9 or ICD-10 MDD (recurrent or moderate-severe single episode) | BIP, SCZ | MDD (self-report); BIP, SCZ; HADS≥7; AD use |
| **GenScotland [4, 5]** | UK | Random sample of patients in general practice; SCID | Drawn from same cohort. SCID | DSM-IV MDD | BIP | MDD |
| **GERA [6]** | USA | Kaiser Permanente Northern California (1995-2013); EMR | Kaiser Permanente Northern California (1995-2013); EMR | ICD-9 MDD on ≥2 separate medical visits | BIP, SCZ | ICD-9 codes MDD, insomnia, stress, irritable bowel syndrome, other psych dx (BIP, NAP, SUD, PTSD, eating disorders, anxiety disorders) |
| **iPSYCH [7]** | Denmark | All births 1981–2005, diagnoses from national psychiatric treatment register | Randomly selected from same birth cohort | ICD-10 MDD | BIP or manic episode | MDD, manic episode or BIP |
| **23andMe [8]** | USA | Commercial direct-to-consumer genomics company, consent for research; self-report | Commercial direct-to-consumer genomics company, consent for research; self-report | MDD (diagnosed with clinical depression or depression diagnosed by a doctor) | BIP, SCZ, autism, ID, multiple personality, Parkinson's disease | As for cases plus self-reported depression, anxiety disorder, fibromyalgia, or AD use |

Abbreviations: AD=antidepressant, BIP=bipolar disorder, CIDI=Composite International Diagnostic Interview, CIDI-SF=CIDI-short form, DIGS=Diagnostic Interview for Genetic Studies, Dx=diagnosis, EMR=electronic medical record, FHx=family history, Hx=history, ID=intellectual disability, NAP=non-affective psychosis, SADS=Schedule for Affective Disorders and Schizophrenia, SCID=Structured Clinical Interview for DSM-IV, SUD=substance use disorder.

**MiXeR results**

Model fit require sufficient enrichment and a clear minimum of log-likelihood curve ("U" shape or inverted bell shape).

Enrichment could be justified from the Q-Q plot (more leftward deflection from the diagonal line indicates more enrichment). We found this for DEP & BMI, DEP & SBP and DEP & DBP, as presented in the manuscript (figure 1) and in suppl.figure1-3, but not for DEP & the other phenotypes (CAD, T2D, CRP, TG, TC, LDL and HDL) (suppl. figure 4-10).

**Replication**

We replicated our results in an independent sample, FinnGen. The replication sample for MDD comprised 17.794 cases and 156.611 controls. Cases were defined by ICD-codes for depressive episode or recurrent depressive episode within Finnish national inpatient, outpatient and cause of death registries. Controls were defined by the absence of an ICD diagnosis for depressive disorder or recurrent depressive disorder. There were no Finnish samples in the primary depression samples.

In line with previous studies, the replication was assessed using sign concordance test [9, 10].

**Suppl. Table 2**

**MtCOJO results**

We did mtCOJO analyses controlling for smoking. The Q-Q plots were similar to the original Q-Q plots.

Analyses after mtCOJO gained 1 additional conjFDR loci for depression and TG and gave 0-2 less loci for depression and all the other phenotypes. The loci were more or less the same. This means that controlling for smoking did not change the results notably.

**Suppl. Table 3**

| **Number of conjFDR loci at 0.05** | |  |
| --- | --- | --- |
| CAD/cardiometabolic trait | No of loci in the orignial analyses | No of loci after adjusting for smoking |
| CAD | 6 | 5 |
| CRP | 8 | 6 |
| T2D | 9 | 8 |
| LDL | 10 | 10 |
| HDL | 14 | 13 |
| TC | 11 | 11 |
| TG | 14 | 15 |
| SBP | 38 | 38 |
| DBP | 31 | 31 |

The results of the smoking corrected analyses (mtCOJO) for depression and SBP and DBP and the other phenotypes except for BMI as described in the main text were similar to the results of the original analyses. Of 4.4 K trait-variants influencing SBP and 4.0 K influencing DBP, 2.1K (SD 0.5 K) and 1.3K (SD 0.5K) were shared with depression, respectively. (Dice coefficient 0.23 (SD 0.06) and 0.14 (SD 0.05) for SBP and DBP, respectively). (Suppl. Fig. 2 & 3).

1. Wray NR, Ripke S, Mattheisen M, Trzaskowski M, Byrne EM, Abdellaoui A, et al. Genome-wide association analyses identify 44 risk variants and refine the genetic architecture of major depression. Nature genetics. 2018;50(5):668-81.

2. Eriksson N, Macpherson JM, Tung JY, Hon LS, Naughton B, Saxonov S, et al. Web-based, participant-driven studies yield novel genetic associations for common traits. PLoS Genet. 2010;6(6):e1000993.

3. Ripke S, Wray NR, Lewis CM, Hamilton SP, Weissman MM, Breen G, et al. A mega-analysis of genome-wide association studies for major depressive disorder. Mol Psychiatry. 2013;18(4):497-511.

4. Fernandez-Pujals AM, Adams MJ, Thomson P, McKechanie AG, Blackwood DH, Smith BH, et al. Epidemiology and Heritability of Major Depressive Disorder, Stratified by Age of Onset, Sex, and Illness Course in Generation Scotland: Scottish Family Health Study (GS:SFHS). PLoS One. 2015;10(11):e0142197.

5. Smith BH, Campbell A, Linksted P, Fitzpatrick B, Jackson C, Kerr SM, et al. Cohort Profile: Generation Scotland: Scottish Family Health Study (GS:SFHS). The study, its participants and their potential for genetic research on health and illness. Int J Epidemiol. 2013;42(3):689-700.

6. Banda Y, Kvale MN, Hoffmann TJ, Hesselson SE, Ranatunga D, Tang H, et al. Characterizing Race/Ethnicity and Genetic Ancestry for 100,000 Subjects in the Genetic Epidemiology Research on Adult Health and Aging (GERA) Cohort. Genetics. 2015;200(4):1285-95.

7. Pedersen CB, Bybjerg-Grauholm J, Pedersen MG, Grove J, Agerbo E, Bækvad-Hansen M, et al. The iPSYCH2012 case–cohort sample: new directions for unravelling genetic and environmental architectures of severe mental disorders. Molecular Psychiatry. 2018;23(1):6-14.

8. Hyde CL, Nagle MW, Tian C, Chen X, Paciga SA, Wendland JR, et al. Identification of 15 genetic loci associated with risk of major depression in individuals of European descent. Nat Genet. 2016;48(9):1031-6.

9. Lee JJ, Wedow R, Okbay A, Kong E, Maghzian O, Zacher M, et al. Gene discovery and polygenic prediction from a genome-wide association study of educational attainment in 1.1 million individuals. Nat Genet. 2018;50(8):1112-21.

10. Savage JE, Jansen PR, Stringer S, Watanabe K, Bryois J, de Leeuw CA, et al. Genome-wide association meta-analysis in 269,867 individuals identifies new genetic and functional links to intelligence. Nat Genet. 2018;50(7):912-9.
