# Supplementary material for: Shared genetic loci between depression and cardiometabolic traits
Source: PLoS Genet. 2022 May 13;18(5):e1010161. doi: 10.1371/journal.pgen.1010161 (PMC9170110; doi:10.1371/journal.pgen.1010161)
Supplement: Attachment — Submitted filename: PLOS_Genetics_Review_20211109_submitted.docx [file pgen.1010161.s056.docx]

Title: “Shared genetic loci between depression and cardiometabolic traits ” Lead Author: Kristin Torgersen

Summary: The authors aimed to identify genetic loci that are implicated in both, depression and coronary artery disease and its associated risk factors. These risk factors included HDL, LDL, BMI, CRP, systolic blood pressure, diastolic blood pressure, type-2-diabetes, total cholesterol and triglycerides. They used MiXeR and pleioFDR to quantify the overlap using GWAS summary statistics data for Depression and each of the CAD risk factors. Lastly they use FUMA to understand the functional consequences of the SNPs which show significance based on association tests. Overall, the paper aims to address and important topic, and provides an approach to investigate genomic sharing among comorbid-complex diseases. However I feel that the paper needs more work to clearly flesh out the claims made. I offer my suggestions below.

Major Points

1. The authors repeatedly mention that the genetic relationship between depression and cardiovascular phenotypes is not known. While the specific genes linking depression and CAD are not yet known, there are many studies characterizing the genetic relationship between depression and CAD^1-5^.I suggest the authors should clarify which elements of the genetic relationship remain unknown and cite existing literature. Some of these papers include mendelian randomization analyses of the same risk factors examined in the present study and thus are critical to the interpretation of findings.
2. Smoking has been associated with both depression and CAD/cardiovascular risk factors. Given the potential for smoking status to confound the analyses, I am interested in seeing how adjusting for smoking using mtCOJO analyses in each pairwise comparison changes the results. Additionally including smoking as a CAD risk factor in itself can provide additional insights.
3. Even though the introduction explains the clinical significance of these conditions well, the motivation of the study needs to be made more prominent. Specifically, the authors should comment on
   a. Why studying the genetics of these conditions is important?
   b. Why and how understanding the comorbid genetic architecture can motivate new research and clinical practices?
   c. Why has this gap in knowledge not been filled in past – What tools/methods do we have now that help in this understanding?
   d. How does the highly complex and polygenic architecture of these conditions affect the motivation of the study?
4. On page 5, the authors mention some prior literature and state “However, genetic correlation returns a single estimate of genetic overlap and require correlation of effect sizes across all SNPs between two traits…” I would disagree with the statement that global genetic correlation requires correlation of effect sizes across all SNPs between two traits. It only requires that the net correlation is different from zero, but there are many SNP effects that are uncorrelated (or anti-correlated) even when there is a detectable global genetic correlation.
5. The authors talk about “causation” throughout the manuscript with respect to the MiXeR models. I’d like the authors to clarify that this refers to the “cause of the statistical association” and not necessarily the biological cause of the conditions studied; a claim which would require additional experimental evidence.
6. In the results, authors talk about causal shared and unique variants in figure 1. I suggest providing the estimates for MiXeR for the rest of the traits in the supplementary information as this helps the reader benchmark the depression/CVD-risk factor results. I can see the authors have shown the qq plots and the Manhattan plots but I think the venn diagrams will be helpful also.
7. For Table 2, I would like the authors to restructure the table to provide more clarity. For example, the header like :
   a. “Dep Z” should be changed to “Z score of the SNP in Depression GWAS” or this should be clarified in the legend.
   b. The significance in the Dep Z column only include two significant figures
   c. The marks trailing in the Dep Z column should be removed
   d. Please clarify what “Novel in Dep” means
   e. Include the build used to determine the base pair position
   f. Add space between Nearest and Gene in the column header “NearestGene”
   g. Please clarify what the “Phenotype” means in the column header or in the legend
8. In the discussion section, the authors state that BMI is strongly influenced by behavior (e.g., depressive behaviors) and thus could be considered a behavioral trait. While true, there is also evidence from mendelian randomization studies that BMI has a causal effect on depression^6^, The authors should consider this possible direction of effect as well.

Minor Points

1. In Table 2: X should be relabeled CAD/Cardiovascular associated trait.
2. IFigure 1 can use a different color scheme ( not sure why BMI, SBP and DBP are in the same color).
3. Venn diagram labelling should be made clearer. Specifically, what is the box below the venn supposed to represent?
4. I would like the authors to explain why only 3 traits have been used in Figure 2 in the main text.
5. I would recommend the authors to add the zoom plots.
6. I would also suggest labelling the lead SNP/SNPs in the Manhattan plots

Bibliography

1. Kendler, K. S., Gardner, C. O., Fiske, A. & Gatz, M. Major depression and coronary artery disease in the Swedish twin registry: phenotypic, genetic, and environmental sources of comorbidity. *Arch. Gen. Psychiatry* **66**, 857–863 (2009).
2. Dennis, J. *et al.* Genetic risk for major depressive disorder and loneliness in sex-specific associations with coronary artery disease. *Mol. Psychiatry* (2019) doi:10.1038/s41380-019-0614-y.
3. 3Lu, Y. *et al.* Genetic Liability to Depression and Risk of Coronary Artery Disease, Myocardial Infarction, and Other Cardiovascular Outcomes. *JAHA* (2020) doi: <https://doi.org/10.1161/JAHA.120.017986>
4. Wium-Anderson, M.K., *et al.* The familial and genetic contribution to the association between depression and cardiovascular disease: a twin cohort study. *Mol. Psychiatry* **26**, pages 4245–4253 (2021)
5. Li, G. H-Y. *et al.* Evaluation of Bi-Directional Causal Association between Depression and Cardiovascular Diseases: a Mendelian Randomization Study. *Psychological Medicine* (2020) doi:10.1017/S0033291720003566.
6. Wray N.R. *et al,* Genome-wide association analyses identify 44 risk variants and refine the genetic architecture of major depression. *Nature genetics.* **50**. 668-581. (2018).
