# Supplementary material for: Shared genetic loci between depression and cardiometabolic traits
Source: PLoS Genet. 2022 May 13;18(5):e1010161. doi: 10.1371/journal.pgen.1010161 (PMC9170110; doi:10.1371/journal.pgen.1010161)
Supplement: Attachment — Submitted filename: TorgersenAccompanying letter Depression and CVD.docx [file pgen.1010161.s057.docx]

To

Jonathan Flint

Senior Editor

PLOS Genetics

Gregory Barsh

Editor-in-Chief

PLOS Genetics

January 27th, 2021

Re: Original scientific paper “Shared genetic loci between depression and cardiometabolic traits” submitted to PLOS Genetics (PGENETICS-D-21-01304) - [EMID:4f5b6b63b3a4fe87]

Dear Editors

Thank you very much for the comments from the reviewers and for inviting us to resubmit a revised version of the manuscript together with responses to the comments.

We appreciate the constructive comments and suggestions from the reviewers. Please find below a point-by-point response to the comments and enclosed the revised version of the manuscript, with changes made according to the comments.

For the convenience of the reviewers, the changes throughout the paper are marked by track changes. We hope you will find the enclosed version of the manuscript suitable for publication in *PLOS Genetics*.

Please do not hesitate to contact us if there are any questions.

Best regards

For the authors,

Kristin Stensland Torgersen MD

**Responses to the reviewer’s comments**

Responses to the comments of Reviewer 1:

Reviewer #1:
*“1. MiXeR: the authors wrote that the model fit for CAD and other CVD risk factors were not good enough to provide reliable estimates for MiXeR when testing for their shared genetic loci with depression, leaving only MiXeR results between depression and 3/10 cardiovascular phenotypes. This seems disappointing for GWAS on cardiovascular phenotypes with relatively large sample sizes (N = 200-700K). It would be great if the authors can give an explanation: what could have caused the poor model fit in all these cases? While this may already be explained in the MiXeR paper, please could the authors expand on explaining why the model didn't work specifically in their own analyses for particular traits? It could be helpful to have the model fits for each of these phenotypes in a supplementary table to demonstrate how they didn't fulfil what criteria.”*

We appreciate the reviewer’s concern about this. We have now included supplementary tables (1 &2) with model parameters for MiXeR and the MiXeR results for the other phenotypes (Suppl. figure 4-10) and this text (page 23, paragraph 3 and 4):

“Unfortunately, the model fit for MiXeR for the other phenotypes was inadequate. This may be due to the fact that all these traits (lipids, CAD, T2D and CRP) have very low polygenicities (univariate polygenicities are shown in suppl. table 2) yet highly heritable, making sampling procedure, which is used in the bivariate optimization procedure very “noisy”. Technically we could try to suppress this “noise” by increasing the number of samples (default is 20K), but computational burden will grow proportionally to the increment of samples, making this infeasible. Therefore, specific details of current implementation (e.g. random sampling procedure to find optimal parameters) limit application domain of MiXeR model to polygenic traits with reasonable heritability.

The second aspect, suggesting why straightforward increase of samples in optimization is not a good solution for this kind of low-polygenic traits is that one will expect some peculiar features of genetic architecture (e.g. few notable strong signals and fairly weak “polygenic tail” or strong tendency of strong signals to be co-localized in the genome). These peculiarities may violate assumptions of MiXeR model (which assumes that statistically “causal” variants are uniformly distributed in the genome and all effect sizes are drawn from the same normal distribution) making even seemingly stable estimates invalid.”

We have included the following text in the supplementary information (page 3 paragraph 1):

“MiXeR results

Model fit require sufficient enrichment and a clear minimum of log-likelihood curve ("U" shape or inverted bell shape).

Enrichment could be justified from the Q-Q plot (more leftward deflection from the diagonal line indicates more enrichment). We found this for DEP & BMI, DEP & SBP and DEP & DBP, as presented in the manuscript (figure 1) and in suppl.figure1-3, but not for DEP & the other phenotypes (CAD, T2D, CRP, TG, TC, LDL and HDL) (suppl. figure 4-10).”

*“2. Conditional QQ plots: the authors wrote that the conditional QQplots "show polygenic enrichment for depression with CAD and all of the CAD factors (Suppl. fig 4-21) - here do they mean all the Dep|other factor analyses or also the other|Dep analyses, or both? Suppl. fig 4-21 cover both ways, please clarify and rewrite to make this clear. Further, the authors wrote in Methods that pleiotropic enrichment "is shown by a successively leftward deflection from the null line on the conditional Q-Q plot." I don't see this in Suppl. fig 4: CAD|DEP and 12: TC|DEP, and it isn't clear to me in 8: LDL|DEP, 10: HDL|DEP; 15: DEP|TG, 16: T2D|DEP, 19: DEP|CRP, because the lines were too close together or the proportion of SNPs associated with a phenotype didn't increase as a function of the strength of the assocation with the conditional phenotype especially at more stringent p value thresholds (yellow and purple lines). How do the authors show that these successive increases in proportions of SNPs associated were significant? I think it is necessary to test for the significance of the enrichment (eg in the condFDR paper by Andreassen et al AJHG 2013) - if the authors already did this, I do not see this presented in the paper, please add explanations of how they performed this test and a supplementary table showing these results.”*

We are grateful for the possibility to clarify this. We have made changes in the text regarding this (page 16, paragraph 2):

“Conditional Q-Q plots show polygenic enrichment for DEP|CAD and DEP|all of the risk factors and for CAD|DEP and all of the risk factors|DEP. We observed a pronounced successive leftward deflection for DEP|SBP, DEP|DBP and SBP|DEP and DBP|DEP and for CRP|DEP, TG|DEP, HDL|DEP, LDL|DEP and DEP|T2D. The enrichment pattern in conditional Q-Q plot for DEP|CAD, CAD|DEP, DEP|TC and TC|DEP is less pronounced, but there is clear trend for successive leftward deflection in the “polygenic” or middle region. Also the leftward deflection was less pronounced in DEP|CRP, DEP|LDL, DEP|HDL, DEP|TC, TC|DEP and T2D|DEP. In Q-Q plot we are primarily interested in the behavior of the middle part where the polygenic component resides and occasionally observed erratic trajectories of tails are driven by a handful of variants which do not reflect trend *en masse*. ”

Q-Q plots is a hypothesis-free tool for visual assessment of enrichment and there is no well-established way to quantify observed leftward deflection. The approach used to calculate significance of enrichment in the original 2013 paper *(e.g. in the condFDR paper by Andreassen et al AJHG 2013)* has later not been used since this approach is not fully statistically valid across all phenotypes. Later work using this method is based on visual assessment of the Q-Q plots only (1-4).

*“4. CondFDR and conjFDR results: How many of the conjFDR loci were found in the condFDR analyses? Please show overlap and interpretations of the results, ie how many of the new loci detected using the condFDR approach (both ways for each phenotype pair) were shown by conjFDR to be shared effects (same or different directions of effect) that indicate shared mechanisms. These should be integrated into Table 1.*

*”*

Thanks for the comment. We have now included how many of the conjFDR loci which was also found in the condFDR analyses in table 1 as suggested be reviewer # 1, and this comment in the text (page 25, paragraph 2, sentence 3):

“33-90% of conjFDR-discovered loci were identified by condFDR. Since conjFDR is the maximum of the two condFDR values and the significance threshold was 0.05 for conjFDR and 0.01 for condFDR, this proportion gives an indication of the strength of association between the set of conjFDR lead SNPs and each phenotype.”

*“4. Replication: It would be great to see some replication of this work. The authors said the GWAS data on depression they used did not contain data from UKBiobank, in order to minimize sample overlap between DEP and other traits in their analyses - this means the authors can ask whether the condFDR/conjFDR hits replicate well in depression in UKBiobank.”*

We thank the reviewer for suggesting a replication analysis, which we agree would improve the article. However, we cannot use UK Biobank for replication since it is used in the conditional trait for CAD and the CVD risk factors so there will still be sample overlap, and conjunctional FDR requires fully independent samples. Instead, we have replicated our results in FinnGen (https://www.finngen.gitbook.io/documentation/). We have added the following text (page 13, paragraph 3 and page 14, paragraph 1):

**“**Replication of condFDR and conjFDR significant loci in an independent sample.

We tested for evidence of consistent genetic effects in an independent major depression sample from FinnGen, following a similar procedure as described in (1). The probability of replicating individual loci at genome-wide significance is low due to weak genetic effects. Therefore, we first tested for en masse sign concordance of effect direction in primary and replication samples, in line with previous literature (5-7). There was significant concordance if the lead SNP had concordant effect directions in the primary and replication depression samples. We used a one-sided exact binomial test of significance under the null hypothesis that significant concordance was randomly distributed (5-7).”

We also added this text under the results (page 18, paragraph 2, sentence2):

“We then replicated the results of the cond/conjFDR analyses in an independent dataset, FinnGen (<https://www.finngen.gitbook.io/documentation/>). Applying a significance threshold of 0.05, all the condFDR results were highly significant, and 5 out of 9 conjFDR analyses were significant. All four non-significant conjFDR analyses had a low number of shared loci discovered (<10) which reduced the statistical power of the exact binomial test. Further details and replication results are provided in the supplementary material and in the compressed zip file results_replication_FINNGENN.”

We added the following text and table in the supplementary information in (page 3, paragraph 2 and page 4, paragraph 1 and 2):

**“Replication**

We replicated our results in an independent sample, FinnGen. The replication sample for MDD comprised 17.794 cases and 156.611 controls. Cases were defined by ICD-codes for depressive episode or recurrent depressive episode within Finnish national inpatient, outpatient and cause of death registries. Controls were defined by the absence of an ICD diagnosis for depressive disorder or recurrent depressive disorder. There were no Finnish samples in the primary depression samples.

In line with previous studies, the replication was assessed using sign concordance test (6, 7).

**Suppl. Table 2**

“

*“Minor*

*1.All supplementary figures and tables need to come with some legends - without these I cannot tell what some columns of supplementary tables are referring to or how they are calculated. If these are already provided and I missed them please could the authors point out clearly where to look for them? I have not been able to find them.”*

Thank you for pointing this out. We have now added this for the figure/suppl. figures. We have also provided the suppl. tables as excel files, these are more user-friendly and provides a clearer view of the information. The pdf suppl. tables unfortunately are not possible to read properly.

*“2. Why were the three manhattan plots in Fig2 chosen instead of the other manhattan plots in supp figure 22-27? Is there any particular reason the authors wants to draw attention to these three conjFDR analyses?”*

We thank the reviewer for this comment. In (page17, paragraph 1, sentence 4),

we have added:

“In Fig. 2 the Manhattan plots for depression and CAD, SBP, LDL are shown as we consider these to be the most clinically relevant. The Manhattan plots for the other phenotypes can be found in the supplementary information.”

Responses to the comments of Reviewer 2:

Reviewer #2: *“Major Points*

*1.The authors repeatedly mention that the genetic relationship between depression and cardiovascular phenotypes is not known. While the specific genes linking depression and CAD are not yet known, there are many studies characterizing the genetic relationship between depression and CAD^1-5^.I suggest the authors should clarify which elements of the genetic relationship remain unknown and cite existing literature. Some of these papers include mendelian randomization analyses of the same risk factors examined in the present study and thus are critical to the interpretation of findings.”*

Thank you for this comment. In accordance with the reviewer’s comment, the manuscript has been revised. In (page 7, paragraph 1) we have specified this:

“While twin studies have found bidirectional associations between depression and CAD (8, 9), the specific genes linking depression and CAD are not known and the cause of this association remain unclear (1). Mendelian randomization has indicated that the genetic liability to depression is associated with higher CAD and myocardial infarction risks, partly mediated by type 2 diabetes mellitus and smoking (10). Furthermore, an increase in polygenic score for depression was associated with CAD (11). However, while mendelian randomization might point to certain causative relationships (12), it does not identify specific genes underlying the relationship.”

*1.“Smoking has been associated with both depression and CAD/cardiovascular risk factors. Given the potential for smoking status to confound the analyses, I am interested in seeing how adjusting for smoking using mtCOJO analyses in each pairwise comparison changes the results. Additionally including smoking as a CAD risk factor in itself can provide additional insights.”*

We are thankful for this very helpful suggestion. We have adjusted for smoking using mtCOJO analyses accordingly. We have added the following under Material and Methods (page 14, paragraph 2):

“We then adjusted for smoking using mtCOJO analyses and rerun all the same analyses to see how adjusting for smoking would affect the results.”

Here are the original MiXeR results for comparison (as described under the result section: The results show evidence of substantial polygenic overlap between depression and BMI. Of 13.9K trait-variants influencing depression and 11K trait-variants influencing BMI, 9.5K (SD 1.0K) were shared. The Dice coefficient was 0.76 (SD 0.08). Of 4.4K trait-variants influencing SBP and 4.0K influencing DBP, 2.0K (SD 0.4 K) and 1.0K (SD 0.3K) were shared with depression, respectively. (Dice coefficient 0.22 (SD 0.04) and 0.11 (SD 0.04) for SBP and DBP, respectively).

We have added the following under Results (page 16, paragraph1, sentence 1):

“The MiXeR results for depression and BMI were somewhat different after mtCOJO analyses correcting for smoking. Of 14.3K trait-variants influencing depression and 6.6 K trait-variants influencing BMI, 5.5K (SD 0.9 K) were shared. The Dice coefficient was 0.52 (SD 0.08) (Compressed suppl. file mtCOJOresults). The results of the smoking corrected analyses for depression and SBP and DBP were similar to the results of the original analyses (Compressed suppl. file mtCOJOresults). For more information, see the supplementary material.”

We added under Discussion (page 22 paragraph 2, sentence 2):

“The difference in the MiXeR results when we corrected for smoking using mtCOJO analyses may further support this.” following this paragraph: MiXeR analyses found that depression and BMI shared a substantial number of variants. Of 13.9K trait-variants influencing depression and 11K trait-variants influencing BMI, 9.5K (SD 1.0K) were shared. The proportion of shared genetic variants is greater than what would be expected based on the moderate genetic correlation between these traits (r_g_ = 0.13 for BMI). This may indicate shared genetic mechanisms with mixed effect directions. One possible explanation for this is that BMI is strongly influenced by behaviour, and could thus to some degree be considered a “behavioural/brain-related trait”. This may explain the large difference in polygenicity when compared to SBP and DBP which could be considered “somatic traits”, and, by extension, the substantially larger number of shared variants (9.5k vs 1/2K). It is also concordant with previous literature that identified odds ratios in the range of 1.0 to 2.0 for the association between obesity and depression. (13).

Here you can see the Venn diagrams of DEP and BMI before and after adjusting for smoking for comparison:

We have also added this section to the supplementary material (page 4, paragraph 3 and page 5 paragraph 1 and 2):

“**MtCOJO results**

We did mtCOJO analyses controlling for smoking. The Q-Q plots were similar to the original Q-Q plots.

Analyses after mtCOJO gained 1 additional conjFDR loci for depression and TG and gave 0-2 less loci for depression and all the other phenotypes. The loci were more or less the same. This means that controlling for smoking did not change the results notably.

**Suppl. Table 3**

| **Number of conjFDR loci at 0.05** | |  |
| --- | --- | --- |
| CAD/cardiometabolic trait | No of loci in the orignial analyses | No of loci after adjusting for smoking |
| CAD | 6 | 5 |
| CRP | 8 | 6 |
| T2D | 9 | 8 |
| LDL | 10 | 10 |
| HDL | 14 | 13 |
| TC | 11 | 11 |
| TG | 14 | 15 |
| SBP | 38 | 38 |
| DBP | 31 | 31 |

The results of the smoking corrected analyses (mtCOJO) for depression and SBP and DBP and the other phenotypes were similar to the results of the original analyses. Of 4.4 K trait-variants influencing SBP and 4.0 K influencing DBP, 2.1K (SD 0.5 K) and 1.3K (SD 0.5K) were shared with depression, respectively. (Dice coefficient 0.23 (SD 0.06) and 0.14 (SD 0.05) for SBP and DBP, respectively). (Suppl. figure 2 & 3).“

We also did include smoking in the initial analyses, but decided to leave it out for further analyses because the smoking GWAS seemed underpowered. This GWAS had only identified 3 loci for smoking (14).

*2.“Even though the introduction explains the clinical significance of these conditions well, the motivation of the study needs to be made more prominent. Specifically, the authors should comment on*

*a)Why studying the genetics of these conditions is important?”*

Thank you for bringing our attention to this. We agree to clarify this better. We have added the sentence (page 5, paragraph 1, last sentence.):

“It is therefore crucial to gain new knowledge about the causes of this comorbidity. Genetic discoveries may also provide important insights for drug development and risk prediction.”

*b)“Why and how understanding the comorbid genetic architecture can motivate new research and clinical practices?”*

We agree that this is very important and have added the following sentence (page 27, paragraph 1, sentence 6):

“Our finding of specific loci underlying the comorbidity of depression and CAD may motivate researchers to investigate the molecular mechanisms driving the statistical association identified. Furthermore,a more detailed understanding of the shared genetic architecture may enable improved prediction or identification of subgroups at high risk for both disorders in clinical practice.”

*c) “Why has this gap in knowledge not been filled in past – What tools/methods do we have now that help in this understanding?”*

Thank you for the opportunity to clarify this. At the end of the Introduction section (page 7, paragraph 1, sentence 4 and onwards) we have revised our description of the tools/methods previously applied and the advantages to the methods we have applied:

“It has been hypothesized that the association between depression and CAD is partly caused by genetic pleiotropy (15), but this has not been evaluated beyond genetic correlations and PRS, and the specific genes linking depression and CAD are not known. Genetic liability to depression has been associated with higher CAD and myocardial infarction risks, partly mediated by type 2 diabetes mellitus and smoking (10). While mendelian randomization might point to certain causative relationships (12), it does not identify specific genes underlying the relationship. Increase in polygenic score for depression was associated with CAD (11). Previous studies have found minimal genetic correlations (r_g_ 0.12 (*p* = 8.47 × 10^−6^) for CAD, r_g_ = 0.13, (*p* = 3.43 × 10^−11^) for BMI, r_g_ = 0.11, (*p* = .0001) for T2D, 0.0108 (p = 0.897) for HDL, -0.189 (p 0.818) for TC and 0.176 (p 0.0203) for TG (16, 17). However, Genetic correlation returns a single estimate of genetic overlap, which averages the correlation of effect sizes across all SNPs, after controlling for LD. It is therefore possible for two traits to have a genetic correlation close to 0 despite regions with strong negative and positive correlation. In brain-related traits including depression and intelligence mixed genetic effects may be present (2, 18, 19).

In contrast, the bivariate causal mixture model (MiXeR) provides an estimate of the total number of shared and unique variants influencing two traits (6). This has enabled the discovery of extensive genetic overlap between brain-related traits with non-significant genetic correlation and mixture of variants with same and opposite effect directions (4, 7-9). This is relevant since it may imply shared variance beyond genetic correlation (10). Determining genetic overlap at the individual locus level is also informative to obtain biological insights. However standard cross-GWAS techniques rely on massive multiple testing, limiting statistical power for discovery (10). In contrast, the conditional/conjunctional false discovery rate method (cond/conjFDR) leverages information from two GWAS to identify shared loci with likely true effects, and thus increase yield from existing GWAS (3, 8, 9).”

*d)“How does the highly complex and polygenic architecture of these conditions affect the motivation of the study?”*

Thank you for giving us the opportunity to expand on this. We have added this section (page 6, paragraph 3.):

“The recent decade’s revolution in genetics, with GWAS, in which several hundred thousand to more than a million single nucleotide polymorphisms (SNPs) are assayed in thousands of individuals, has now given new opportunities to assess if there is a shared genetic basis for common psychiatric diseases and CAD risk factors and has motivated us to do this study. Given the high number of traits in humans and the relative small number of genes (20,000), some genes have to affect multiple traits (genetic pleiotropy) (11).”

And (page 8, paragraph 2, sentence 2):

“I.e. given evidence that depression is associated with ~14,000 variants, it is overly simplistic to reduce its genetic relationship with CVD to a single sum score (i.e. genetic correlation). Therefore we wanted to apply mixer, which is able to uncover more complex genetic relationships which account for both same and opposite genetic effects, as we have identified in several other similar trait (1, 2, 20).”

*4.“On page 5, the authors mention some prior literature and state “However, genetic correlation returns a single estimate of genetic overlap and require correlation of effect sizes across all SNPs between two traits…” I would disagree with the statement that global genetic correlation requires correlation of effect sizes across all SNPs between two traits. It only requires that the net correlation is different from zero, but there are many SNP effects that are uncorrelated (or anti-correlated) even when there is a detectable global genetic correlation.”*

We agree that the formulation is a bit misleading and have changed it to (page 7, paragraph1, last 2 sentences):

“Genetic correlation returns a single estimate of genetic overlap which averages the correlation of effect sizes across all SNPs, after controlling for LD. It is therefore possible for two traits to have a genetic correlation close to 0 despite regions with strong negative and positive correlation. In brain-related traits including depression and intelligence mixed genetic effects may be present (3-5).”

*5.“The authors talk about “causation” throughout the manuscript with respect to the MiXeR models. I’d like the authors to clarify that this refers to the “cause of the statistical association” and not necessarily the biological cause of the conditions studied; a claim which would require additional experimental evidence.”*

Thank you for pointing this out. We agree and have gone through the manuscript to correct this. We have emphasized this (page 10, paragraph 3, sentence 3):

“The term “causal” in the bivariate mixture model refers to the cause of the statistical association and not necessarily the biological cause of the conditions studied.” in (page 8, paragraph 3, sentence 1). We have also added “Causal” refers to the *cause of the statistical association” and not necessarily the biological cause of the conditions studied*.”

*6.“In the results, authors talk about causal shared and unique variants in figure 1. I suggest providing the estimates for MiXeR for the rest of the traits in the supplementary information as this helps the reader benchmark the depression/CVD-risk factor results. I can see the authors have shown the qq plots and the Manhattan plots but I think the venn diagrams will be helpful also.”*

Thank you for pointing this out, we have now included the estimates for MiXeR for the rest of the traits in the supplementary information.

*7.“For Table 2, I would like the authors to restructure the table to provide more clarity. For example, the header like :*

a. *“Dep Z” should be changed to “Z score of the SNP in Depression GWAS” or this should be clarified in the legend.”*

Thank you, this has now been done.

b. *“The significance in the Dep Z column only include two significant figures”*

We have changed this to two significant figures.

c. *“The marks trailing in the Dep Z column should be removed”*

We have removed them.

d. *“Please clarify what “Novel in Dep” means”*

“Novel in dep” is now explained in the table legend.

e. *“Include the build used to determine the base pair position”*

We have added:

“The build used to determine the base pair position was GRCh37/Hg19” in all the relevant tables and suppl. tables.

f. *“Add space between Nearest and Gene in the column header “NearestGene””*

Thank you for pointing this out, we have corrected this.

g. *“Please clarify what the “Phenotype” means in the column header or in the legend”*

We have now explained this in the figure legend.

*8.“In the discussion section, the authors state that BMI is strongly influenced by behavior (e.g., depressive behaviors) and thus could be considered a behavioral trait. While true, there is also evidence from mendelian randomization studies that BMI has a causal effect on depression^6^, The authors should consider this possible direction of effect as well.”*

We are grateful that you brought our attention to this and have included (page 22, paragraph 2, last sentence):

“However Mendelian randomization analyses have provided evidence for a 1.12-fold increase in depression per s.d. of BMI which supports that BMI has a causal effect on depression.”

*«Minor Points*

1. *In Table 2: X should be relabeled CAD/Cardiovascular associated trait.”*

We are thankful for this comment and have changed it accordingly.

2. *“In Figure 1 can use a different color scheme (not sure why BMI, SBP and DBP are in the same color).”*

We are grateful for this comment and have now changed the colors.

3. *“Venn diagram labelling should be made clearer. Specifically, what is the box below the venn supposed to represent?”*

We have removed the boxes under the Venn diagrams to make the figure cleaner.

4. *“I would like the authors to explain why only 3 traits have been used in Figure 2 in the main text.”*

We have added (page 17, paragraph 1, sentence 4):

“ In Fig. 2 the Manhattan plots for depression and CAD, SBP, LDL are shown as we consider them to be the most clinically relevant. The Manhattan plots for the other phenotypes can be found in the supplementary information.

5. *“I would recommend the authors to add the zoom plots.”*

We have added (page 17, paragraph 2, sentence 2):

“Depression and CAD and 5 of the CVD risk factors (T2D, HDL, LDL, TC, TG and DBP) were associated with largely overlapping regions on chromosome 11 with lead SNPs being in strong LD (r2>0.7). Fatty Acid Desaturase 2 (*FADS2) gene* was the nearest gene for all these lead SNPs*.* Zoom plots centered on these lead SNPs for all the phenotypes can be viewed in suppl. table 35-40.*”*

6. *“I would also suggest labelling the lead SNP/SNPs in the Manhattan plots.”*

We think this is a good suggestion and have now added labels for the nearest genes in the Manhattan plots shown in the manuscript.

1.	Bahrami S, Hindley G, Winsvold BS, O'Connell KS, Frei O, Shadrin A, et al. Dissecting the shared genetic basis of migraine and mental disorders using novel statistical tools. Brain. 2021.

2.	Bahrami S, Shadrin A, Frei O, O’Connell KS, Bettella F, Krull F, et al. Genetic loci shared between major depression and intelligence with mixed directions of effect. Nature Human Behaviour. 2021.

3.	Cheng W, Frei O, van der Meer D, Wang Y, O'Connell KS, Chu Y, et al. Genetic Association Between Schizophrenia and Cortical Brain Surface Area and Thickness. JAMA Psychiatry. 2021;78(9):1020-30.

4.	Hindley G, Bahrami S, Steen NE, O’Connell KS, Frei O, Shadrin A, et al. Characterising the shared genetic determinants of bipolar disorder, schizophrenia and risk-taking. Translational Psychiatry. 2021;11(1):466.

5.	Consortium TSWGotPG, Ripke S, Walters JT, O’Donovan MC. Mapping genomic loci prioritises genes and implicates synaptic biology in schizophrenia. medRxiv. 2020:2020.09.12.20192922.

6.	Lee JJ, Wedow R, Okbay A, Kong E, Maghzian O, Zacher M, et al. Gene discovery and polygenic prediction from a genome-wide association study of educational attainment in 1.1 million individuals. Nat Genet. 2018;50(8):1112-21.

7.	Savage JE, Jansen PR, Stringer S, Watanabe K, Bryois J, de Leeuw CA, et al. Genome-wide association meta-analysis in 269,867 individuals identifies new genetic and functional links to intelligence. Nat Genet. 2018;50(7):912-9.

8.	Wium-Andersen MK, Villumsen MD, Wium-Andersen IK, Jørgensen MB, Hjelmborg JvB, Christensen K, et al. The familial and genetic contribution to the association between depression and cardiovascular disease: a twin cohort study. Molecular Psychiatry. 2021;26(8):4245-53.

9.	Kendler KS, Gardner CO, Fiske A, Gatz M. Major depression and coronary artery disease in the Swedish twin registry: phenotypic, genetic, and environmental sources of comorbidity. Archives of general psychiatry. 2009;66(8):857-63.

10.	Lu Y, Wang Z, Georgakis MK, Lin H, Zheng L. Genetic Liability to Depression and Risk of Coronary Artery Disease, Myocardial Infarction, and Other Cardiovascular Outcomes. Journal of the American Heart Association. 2021;10(1):e017986-e.

11.	Dennis J, Sealock J, Levinson RT, Farber-Eger E, Franco J, Fong S, et al. Genetic risk for major depressive disorder and loneliness in sex-specific associations with coronary artery disease. Molecular psychiatry. 2021;26(8):4254-64.

12.	Li GH-Y, Cheung C-L, Chung AK-K, Cheung BM-Y, Wong IC-K, Fok MLY, et al. Evaluation of bi-directional causal association between depression and cardiovascular diseases: a Mendelian randomization study. Psychological Medicine. 2020:1-12.

13.	Faith MS, Butryn M, Wadden TA, Fabricatore A, Nguyen AM, Heymsfield SB. Evidence for prospective associations among depression and obesity in population-based studies. Obes Rev. 2011;12(5):e438-53.

14.	Furberg H, Kim Y, Dackor J, Boerwinkle E, Franceschini N, Ardissino D, et al. Genome-wide meta-analyses identify multiple loci associated with smoking behavior. Nature Genetics. 2010;42(5):441-7.

15.	Wu Y, Zhu B, Chen Z, Duan J, Luo A, Yang L, et al. New Insights Into the Comorbidity of Coronary Heart Disease and Depression. Current Problems in Cardiology. 2019:100413.

16.	Wong BC, Chau CK, Ao FK, Mo CH, Wong SY, Wong YH, et al. Differential associations of depression-related phenotypes with cardiometabolic risks: Polygenic analyses and exploring shared genetic variants and pathways. Depress Anxiety. 2019;36(4):330-44.

17.	Hagenaars SP, Coleman JRI, Choi SW, Gaspar H, Adams MJ, Howard DM, et al. Genetic comorbidity between major depression and cardio-metabolic traits, stratified by age at onset of major depression. Am J Med Genet B Neuropsychiatr Genet. 2020;183(6):309-30.

18.	Smeland OB, Bahrami S, Frei O, Shadrin A, O'Connell K, Savage J, et al. Genome-wide analysis reveals extensive genetic overlap between schizophrenia, bipolar disorder, and intelligence. Mol Psychiatry. 2019.

19.	Smeland OB, Frei O, Dale AM, Andreassen OA. The polygenic architecture of schizophrenia - rethinking pathogenesis and nosology. Nat Rev Neurol. 2020.

20.	Bahrami S, Steen NE, Shadrin A, O’Connell K, Frei O, Bettella F, et al. Shared Genetic Loci Between Body Mass Index and Major Psychiatric Disorders: A Genome-wide Association Study. JAMA Psychiatry. 2020;77(5):503-12.
