# Supplementary material for: Shared genetic loci between depression and cardiometabolic traits
Source: PLoS Genet. 2022 May 13;18(5):e1010161. doi: 10.1371/journal.pgen.1010161 (PMC9170110; doi:10.1371/journal.pgen.1010161)
Supplement: Attachment — Submitted filename: Plos_Genetics_Reviews.docx [file pgen.1010161.s058.docx]

Reviewer

All my comments have been addressed. I don’t have any further comments or reviews.

(Anonymous)
